# Synthesis of novel fluorinated building blocks via halofluorination and related reactions

**DOI:** 10.3762/bjoc.16.208

**Published:** 2020-10-16

**Authors:** Attila Márió Remete, Tamás T Novák, Melinda Nonn, Matti Haukka, Ferenc Fülöp, Loránd Kiss

**Affiliations:** 1 Institute of Pharmaceutical Chemistry, University of Szeged, H-6720 Szeged, Eötvös u. 6, Hungary; 2 Interdisciplinary Excellence Centre, Institute of Pharmaceutical Chemistry, University of Szeged, H-6720 Szeged, Eötvös u. 6, Hungary; 3 MTA-SZTE Stereochemistry Research Group, Hungarian Academy of Sciences, H-6720 Szeged, Eötvös u. 6, Hungary; 4 Department of Chemistry, University of Jyväskylä, FIN-40014, Jyväskylä, Finland

**Keywords:** fluorine, fluoroselenation, functionalization, halofluorination, stereocontrol

## Abstract

A study exploring halofluorination and fluoroselenation of some cyclic olefins, such as diesters, imides, and lactams with varied functionalization patterns and different structural architectures is described. The synthetic methodologies were based on electrophilic activation through halonium ions of the ring olefin bonds, followed by nucleophilic fluorination with Deoxo-Fluor^®^. The fluorine-containing products thus obtained were subjected to elimination reactions, yielding various fluorine-containing small-molecular entities.

## Introduction

The effects of fluorine on lipophilicity, metabolism, binding, and bioavailability are often beneficial [1–9]. Fluorinated organic compounds are common amongst drugs [8–9], and the synthesis of fluorinated compounds has become a rapidly developing area [9–18]. In addition to discovering new reagents and conditions for the introduction of fluorine and fluorine-containing groups into a certain organic molecule [11–18], several known methods have been studied and improved over the past decade.

**Scheme 1:**
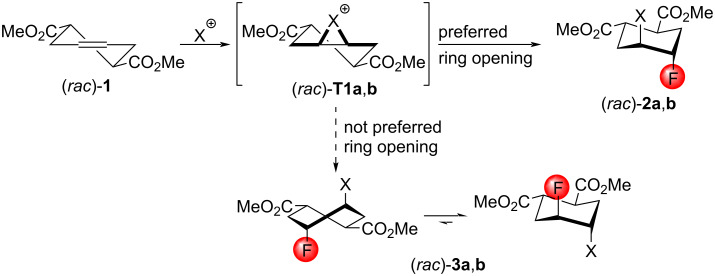
Proposed outcome of the halofluorination of (rac)-**1**. Only the main conformers of (rac)-**1** and (rac)-**T1a**,**b** are shown. (rac)-**2a**: X = Br; (rac)-**2b**: X = I.

One such long-known method is halofluorination. During this process, an alkene reacts with a halogen cation to form a halonium ion, which immediately undergoes ring opening by fluoride to form a vicinal halofluoride (see Scheme 1). The overall result is an *anti*-addition of the XF moiety (X = Cl, Br, I) across the double bond. Since many nucleophilic fluorine sources (e.g., Et_3_N⋅3HF and HF–pyridine) and halonium ion sources (e.g., *N*-halosuccinimides) are relatively cheap and easily available, halofluorination might be an economic way to obtain vicinal halofluorides that can be transformed further in various ways thanks to the different leaving group abilities of halogens [19]. Halofluorinaton-related reactions, such as fluorosulfuration, fluoroselenation, nitrofluorination, and nitriminofluorination, in contrast, are much less studied [20].

Deoxyfluorination, that is fluorine introduction accompanied by oxygen removal (subtypes: OH→F exchange, C=O→CF_2_ transformation, and COOH→CF_3_ transformation), is also an important nucleophilic fluorination method [2–5,10–11,18]. The synthesis of fluorinated compounds via deoxyfluorination [21–30] or utilizing sulfur fluoride deoxyfluorinating reagents [31–32] is a highlighted topic. It is notable that halofluorination reactions applying sulfur fluoride deoxyfluorinating reagents as fluoride sources are practically unknown [33], but analogous reactions with α-fluoroamines (another class of deoxyfluorinating reagents) were reported [20]. As a result, our main aim was to investigate the use of bis(2-methoxyethyl)aminosulfur trifluoride (Deoxo-Fluor^®^) as a fluoride source in halofluorination reactions. We also intended to study fluoroselenations with Deoxo-Fluor^®^ and phenylselenyl bromide (a previously unreported reagent combination).

## Results and Discussion

As starting compounds, we selected some functionalized (to obtain valuable building blocks), cyclic (to obtain better insight into the stereochemistry), and usually symmetrical olefins (to eliminate regioselectivity issues). The first substrate, the cyclohexene diester (*rac*)*-***1**, has a twofold rotational symmetry, that is the formation of only a single halonium ion intermediate is possible. According to the Fürst–Plattner rule (ring-opening into a chair conformation is preferred over a twist boat conformation), the formation of the products (*rac*)*-***2a**,**b** is expected (Scheme 1).

**Scheme 2:**
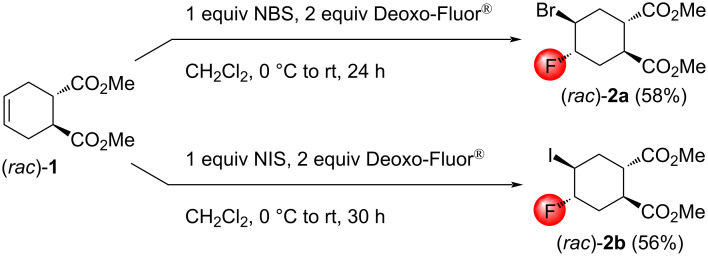
Halofluorination reactions of the *trans*-diester (*rac*)*-***1**.

Indeed, halofluorinations of (*rac*)*-***1** with NBS/Deoxo-Fluor^®^ and NIS/Deoxo-Fluor^®^ systems in anhydrous CH_2_Cl_2_ afforded the compounds (*rac*)*-***2a** and (*rac*)*-***2b** as single products. The stereochemistry of (*rac*)*-***2a** and (*rac*)*-***2b** was determined using NOESY experiments. The reactions proceeded smoothly, although the yields were moderate (Scheme 2).

**Scheme 3:**
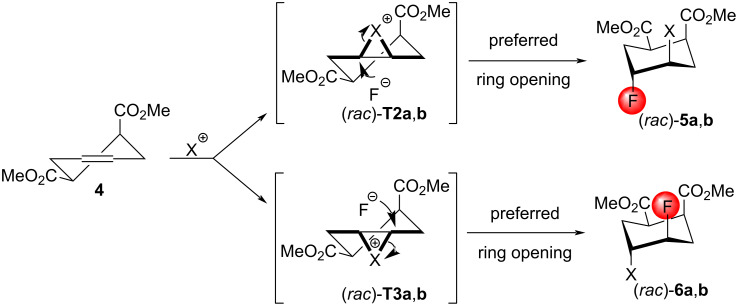
Probable outcomes of the halofluorination of **4**. Both conformers of the compounds **4**, (*rac*)*-***T2a**,**b**, and (*rac*)*-***T3a**,**b**, respectively, have an equal energy. (*rac*)*-***5a** and (*rac*)*-***6a**: X = Br; (*rac*)*-***5b** and (*rac*)*-***6b**: X = I.

Our experiments were continued with compound **4**, the *cis* isomer of the diester (*rac*)*-***1**. In this case, two different halonium ion intermediates can be formed, leading to the possible products (*rac*)*-***5a**,**b** and (*rac*)*-***6a**,**b** (Scheme 3). Since the axial ester group somewhat hinders the attack of the large halonium ion on that side of the ring, the preferred formation of (*rac*)*-***6a**,**b** was expected.

**Scheme 4:**
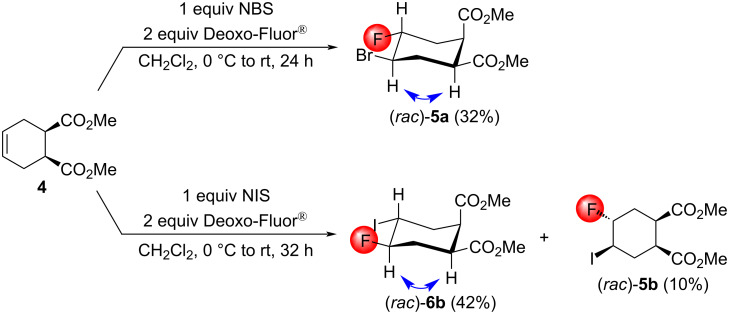
Halofluorination reactions of the *cis*-diester **4**. Important NOESY interactions are indicated by two-headed arrows.

Contrary to our expectations, the bromofluorination of **4** led to the product (*rac*)*-***5a** in 32% yield. The iodofluorination proceeded differently since the reaction was more effective and resulted in a product mixture. As originally expected, a mixture of the main product (*rac*)*-***6b** and the minor product (*rac*)*-***5b** was formed. The stereochemistry was assigned on the basis of NOESY cross-peaks (Scheme 4).

In subsequent studies, halofluorinations of some less symmetrically functionalized cyclohexenes were attempted. Upon treatment with 1 equiv NBS and 2 equiv Deoxo-Fluor^®^ (CH_2_Cl_2_, 0 °C to rt, 2.5 h), benzyl cyclohex-3-ene-1-ylcarbamate quickly produced a multicomponent mixture. Unfortunately, however, no halofluorination product could be isolated. Under similar conditions (1 equiv NBS, 2 equiv Deoxo-Fluor^®^, CH_2_Cl_2_, 0 °C to rt, 24 h), benzyl cyclohex-3-ene-1-carboxylate yielded a mixture of two isomeric bromofluorinated products (≈12:10 ratio, combined yield 66%), with the separation being unsuccessful. Methyl cyclohex-3-ene-1-carboxylate behaved similarly, resulting in an inseparable mixture (1 equiv NBS, 2 equiv Deoxo-Fluor^®^, CH_2_Cl_2_, 0 °C to rt, 27 h, 61% yield of two isomeric bromofluorinated products in a ≈11:10 ratio).

**Scheme 5:**
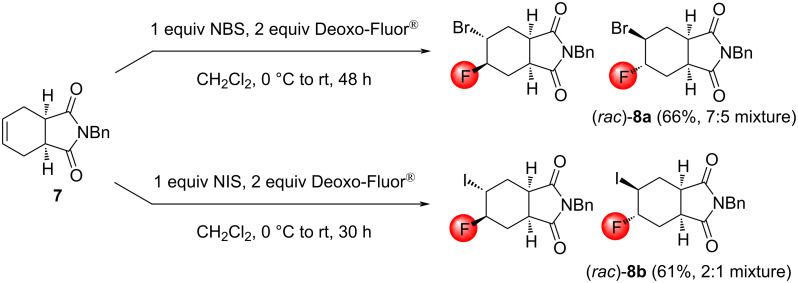
Halofluorination reactions of the *cis*-tetrahydrophthalic imide derivative **7**.

We continued our synthetic explorations with unsaturated fused ring systems. Our first choice was the easily accessible N-benzylated *cis*-tetrahydrophthalic imide **7**. Unfortunately, both bromo- and iodofluorination of **7** yielded a mixture of two isomeric halofluorinated products for which the separation failed (Scheme 5).

**Scheme 6:**
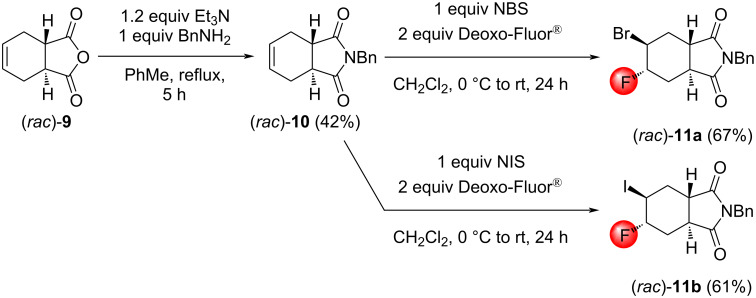
Synthesis and halofluorination of the *trans*-imide (*rac*)*-***10**.

**Figure 1:**
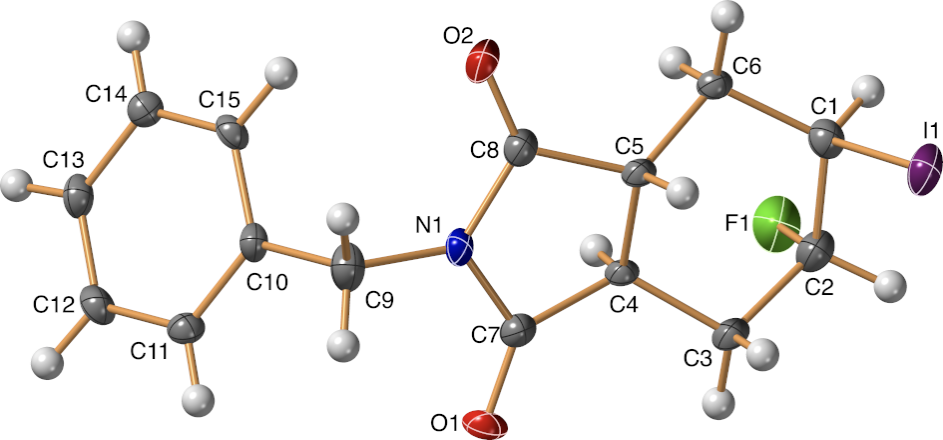
Crystal structure of (*rac*)*-***11b**.

Since halofluorinations of the *trans*-diester (*rac*)*-***1** proceeded more selectively than analogous reactions of the stereoisomeric *cis*-diester **4**, it seemed reasonable to prepare the N-benzylated *trans*-fused tetrahydrophthalic imide (*rac*)*-***10** that was previously unknown in the literature. The synthesis started from the *trans*-tetrahydrophthalic anhydride (*rac*)*-***9** [34], and the reaction of (*rac*)*-***9** with BnNH_2_ in the presence of Et_3_N in toluene under reflux [35] gave the desired product (*rac*)*-***10** in 42% yield. Halofluorinations of the compound (*rac*)*-***10** proceeded smoothly and, according to our expectations, afforded (*rac*)*-***11a** and (*rac*)*-***11b**, respectively, as single products (Scheme 6). The stereochemistry of these products was originally determined by NOESY measurements. In addition, the structure of (*rac*)*-***11b** was unequivocally established by single-crystal X-ray diffraction (Figure 1).

**Scheme 7:**
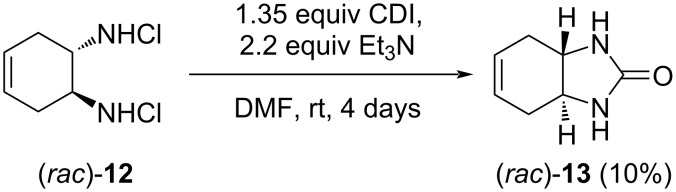
Synthesis of the cyclic carbamide (*rac*)*-***13**.

Then, the preparation of another model compound, the *trans*-annelated bicyclic carbamide derivative (*rac*)*-***13**, was attempted. This compound was also unknown in the literature. In the reaction of commercially available *trans*-4-cyclohexene-1,2-diamine dihydrochloride and 1,1’-carbonyldiimidazole (CDI), through a slightly modified literature protocol [36], the desired product was isolated in only 10% yield (Scheme 7). Increasing the reaction temperature considerably decreased the product purity. With the low yield in mind, further plans concerning compound (*rac*)*-***13** were abandoned.

**Scheme 8:**
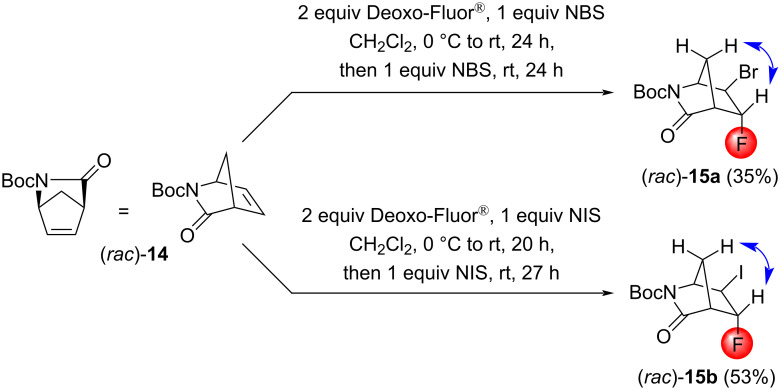
Halofluorination reactions of the γ-lactam (*rac*)*-***14**. Relevant NOESY interactions are indicated by two-headed arrows.

**Figure 2:**
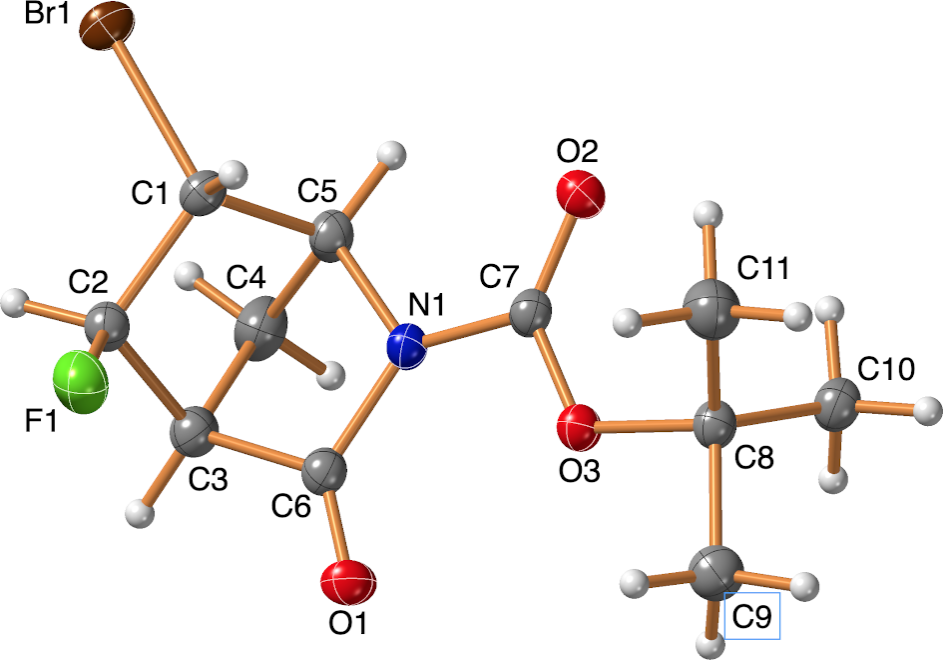
Crystal structure of the product (*rac*)*-***15a**.

**Figure 3:**
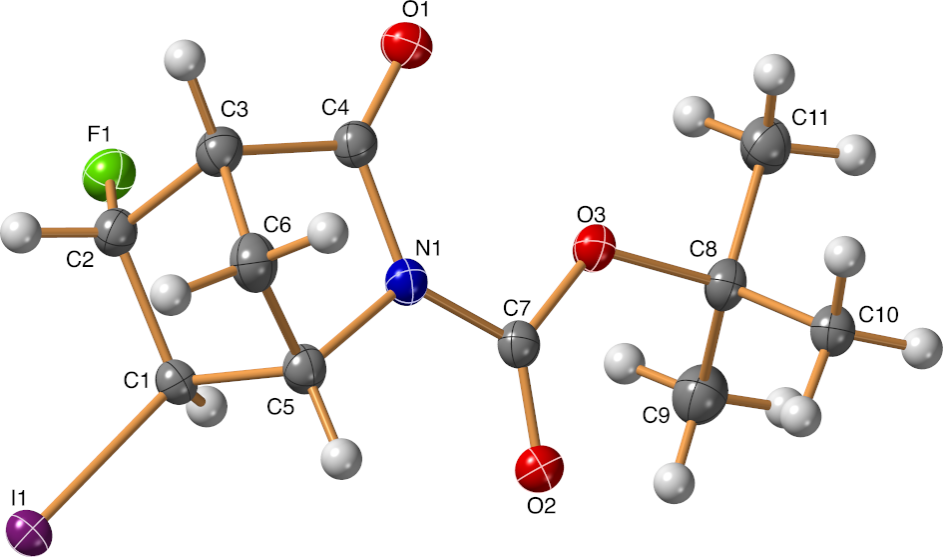
Crystal structure of the product (*rac*)*-***15b**.

We continued our studies with strained, rigid bicyclic systems. First, the *N*-Boc-protected Vince lactam (*rac*)*-***14** was subjected to halofluorination reactions. Despite the asymmetric nature of the olefin, the compounds (*rac*)*-***15a** and (*rac*)*-***15b** were formed as single products, without any regio- or stereoselectivity issues (Scheme 8). However, repeated *N*-halosuccinimide addition was necessary, and the yield of the bromofluorination reaction was still mediocre. The stereochemistry of the products (*rac*)*-***15a** and (*rac*)*-***15b** was determined by both NOESY measurements and single-crystal X-ray diffraction (Figure 2 and Figure 3).

**Scheme 9:**
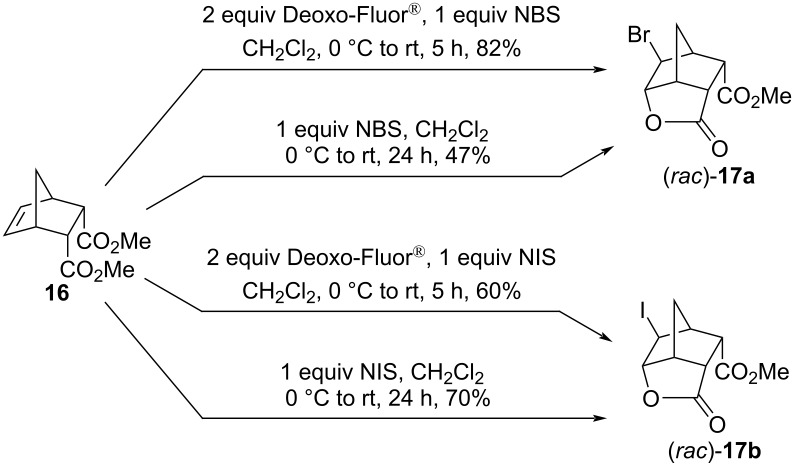
Reactions of the diester **16** with NBS or NIS in the presence or absence of Deoxo-Fluor^®^.

The next rigid bicyclic system studied was the methyl diendo norbornene dicarboxylate **16**. The treatment of **16** with NBS/Deoxo-Fluor^®^ yielded the bromolactone (*rac*)*-***17a** [37]. The reaction, when repeated only with NBS (without a fluoride source), gave the same product. Similarly, subjecting the diester **16** to NIS or NIS/Deoxo-Fluor^®^ resulted in the iodolactone (*rac*)*-***17b** (Scheme 9) [37]. Interestingly, the bromolactonization was more efficient in the presence of Deoxo-Fluor^®^, while the iodofluorination worked better in the absence of the reagent. It is also worth mentioning that the bromolactone (*rac*)*-***17a** was surprisingly stable against lactone ring-opening: the compound remained intact after 20 hours of reflux in methanol in the presence of a catalytic amount of H_2_SO_4_.

**Scheme 10:**
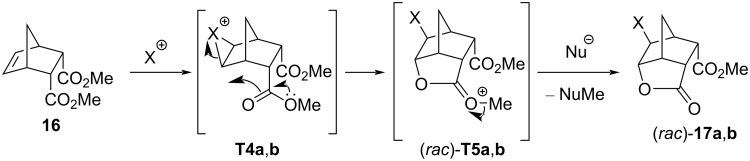
Formation of the halolactons (*rac*)*-***17a**,**b**. The initial attack of the halogen cation occurs at the sterically more accessible side of the C=C bond; (*rac*)*-***17a**: X = Br; (*rac*)*-***2a**: X = I.

Presumably, the halogen cation attacks from the less-hindered side of the carbon–carbon double bond, yielding the halonium ion **T4a**,**b** (Scheme 10). In a subsequent nucleophilic attack, the carbonyl oxygen atom, as an intramolecular nucleophile, competes successfully with the external nucleophilic fluoride ion, resulting in the intermediate (*rac*)*-***T5a**,**b**, followed by the transfer of a methyl group to a nearby nucleophile (for example, F^−^ or the succinimidate anion).

**Scheme 11:**
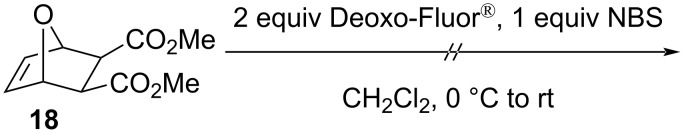
Unsuccessful halofluorination of the bicyclic diester **18**.

We assumed that changing the arrangement of the ester groups from *endo* to *exo* (increasing the distance between the C=C bond and the carbonyl oxygen atom) would render an intramolecular cyclization impossible. Therefore, we attempted the bromofluorination of the easily accessible oxabicycloheptene derivative **18**. Unfortunately, the reaction led to a complex mixture, without any halofluorinated product (Scheme 11).

**Scheme 12:**
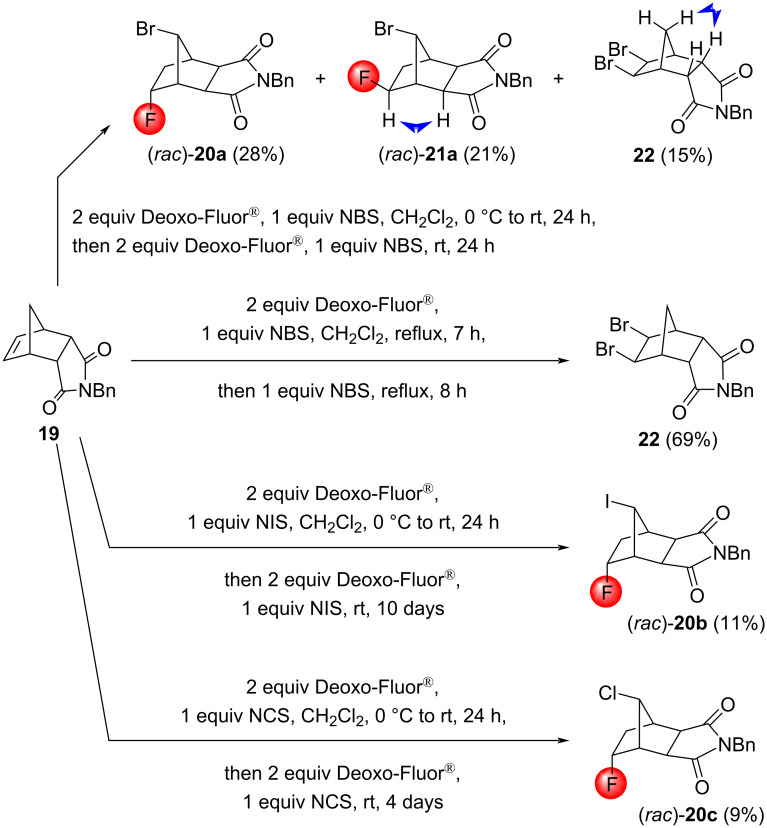
Halofluorination reactions of the rigid tricyclic imine **19**. The relevant NOESY interactions are marked with two-headed arrows.

As an alternative approach, the *N*-benzyl imide **19** was investigated under halofluorination conditions. We expected that the imide moiety of this molecule would fix the carbonyl oxygen atom in a position remote to the C=C bond to avoid cyclization. Indeed, the bromofluorination of tricyclic **19** was successful. However, the process required repeated reagent addition and involved a rearrangement, providing the isomeric products (*rac*)*-***20a** and (*rac*)*-***21a** (Scheme 12). We also observed the formation of the dibrominated compound **22**, which became the sole product when the reaction was performed under reflux conditions. Iodofluorination was much less effective: even after repeated reagent addition and a prolonged reaction time, 46% of the unreacted starting material could be retrieved, together with 11% of the iodofluorinated product (*rac*)*-***20b**. Another halofluorinated product was also formed, but the isolation of the compound in a pure form failed. Attempts to perform this iodofluorination at reflux afforded only unreacted starting material. Since iodofluorination is usually more efficient than bromofluorination [19], the decreased yield of (*rac*)*-***20b** compared to that of (*rac*)*-***20a** is possibly caused by steric hindrance around the olefin bond, which affects the electrophilic attack of the larger iodine cation to a greater extent. With this in mind, we also tried the chlorofluorination of compound **19**. Again, two chlorofluorinated products were formed, but only (*rac*)*-***20c** could be isolated in a pure form (Scheme 12). Chlorofluorination is usually inferior in terms of the yield when compared to bromo- and iodofluorination. However, since Cl^+^ is smaller and less sensitive to steric hindrance than Br^+^ or I^+^, the yield of chloro- and iodofluorination were comparable in this case. The stereochemistry of the above products was determined using NOESY measurements.

**Scheme 13:**
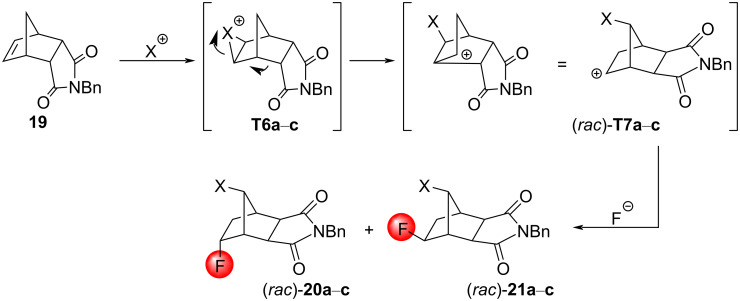
Mechanism of the halofluorination reactions of the substrate **19**. X = Br (compounds a), I (compounds b), Cl (compounds c).

The formation of two isomeric halofluorination products can be explained by the preferred formation of the halonium ions **T6a**–**c**, respectively, since the halogen cation attacks the C=C bond of the imide **19** from the less hindered side, followed by rearrangement into the intermediates (*rac*)*-***T7a**–**c**, respectively. For epoxides and bromonium ions of norbornene systems, such rearrangements are not uncommon [38–40]. Then, the carbenium ion motif of (*rac*)*-***T7a**–**c** can be attacked from both sides to give the products (*rac*)*-***20a**–**c** and (*rac*)*-***21a**–**c** (Scheme 13). The formation mechanism of the product **22**, however, is still an open question. Since the treatment of the reaction mixture at reflux conditions significantly increased the yield, the formation of **22** may follow a radical pathway.

**Scheme 14:**
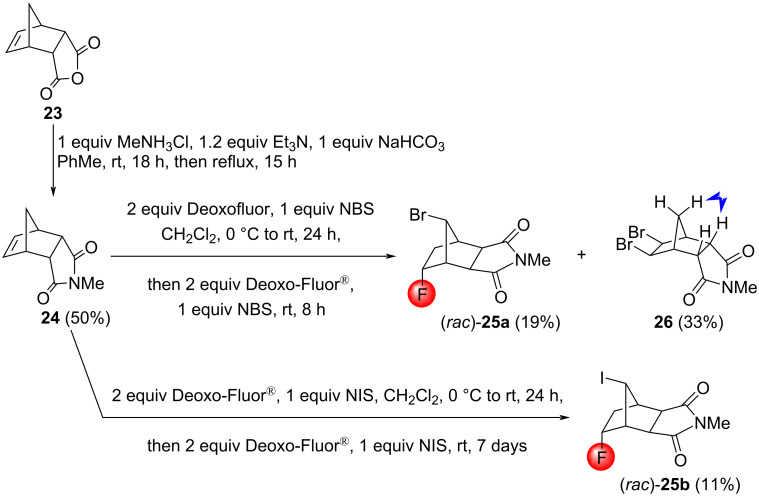
Synthesis and halofluorination of the imide **24**.

Using a modified literature protocol [35], the tricyclic imide **24** was also prepared and investigated. In halofluorination reactions, the behavior of **24** was similar to the *N*-benzyl analogue **19**. The treatment of **24** with Deoxo-Fluor^®^/NBS yielded two bromofluorinated products and the dibrominated compound **26**. Unfortunately, from the two bromofluorinated products, only (*rac*)*-***25a** could be isolated in a pure form (Scheme 14). The reaction of **24** with NIS/Deoxo-Fluor^®^ gave two iodofluorinated products, but only (*rac*)*-***25b** could be isolated in a pure form. The stereochemistry of the products (*rac*)*-***25a**,**b** was determined using NOESY measurements. It is worth noting that the iodofluorination was less effective than the bromofluorination – presumably because of steric hindrance – and 33% unreacted starting material was recovered from the iodofluorination reaction mixture.

In order to study the synthetic usefulness of the halofluorinated products, E2 hydrogen halide elimination was attempted. Since bromide and iodide are much better leaving groups than fluoride, the selective elimination of the former halogens was expected. Taking into account the stereochemical requirements of the E2 elimination (the halide group and the β-hydrogen atom should be antiperiplanar relative to each other), the limited availability of the compound (*rac*)*-***5b**, and Bredt’s rule (bridgehead alkenes are only stable in large ring systems), elimination was attempted only with (*rac*)*-***2a**,**b**, (*rac*)*-***5a**, (*rac*)*-***6b**, (*rac*)*-***8a**,**b**, and (*rac*)*-***11a**,**b**.

**Scheme 15:**
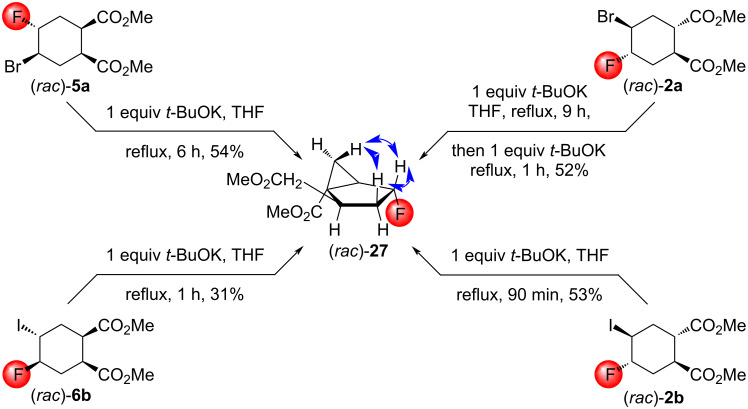
Cyclizations of halofluorinated diesters with potassium *tert*-butoxide. Relevant NOESY interactions are marked with blue two-headed arrows.

DBU in THF under reflux was insufficient to promote the elimination of the halofluorinated diesters (*rac*)*-***2a**,**b**, (*rac*)*-***5a**, and (*rac*)*-***6b**. In contrast, the treatment with *t*-BuOK in THF under reflux was effective. Unexpectedly, all four halofluorinated diesters produced the same condensed ring cyclopropane derivative (*rac*)*-***27** (Scheme 15). The stereochemistry of (*rac*)*-***27** was determined using NOESY measurements.

**Scheme 16:**
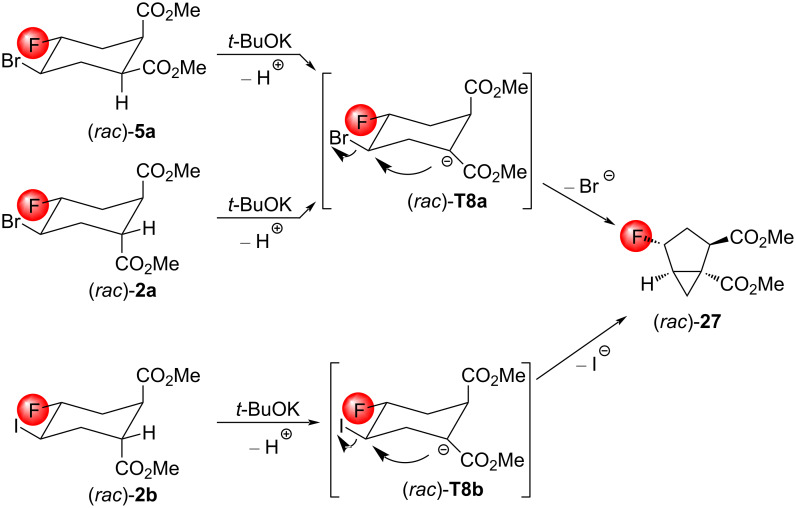
Mechanism of the reaction of the cyclopropanation of the compounds (*rac*)*-***2a**,**b** and (*rac*)*-***5a** with *t*-BuOK.

Regarding the compounds (*rac*)*-***2a**,**b** and (*rac*)*-***5a**, the mechanism is relatively straightforward. Instead of an E2 elimination, these compounds are deprotonated next to the ester group, which is closer to the CHBr unit. Then, the carbanion motif of the formed enolates (*rac*)*-***T8a** and **T8b** afford the cyclopropane-fused cyclopentane dicarboxylate (*rac*)*-***27** upon the intramolecular nucleophilic attack by the alkyl halide (Scheme 16).

**Scheme 17:**
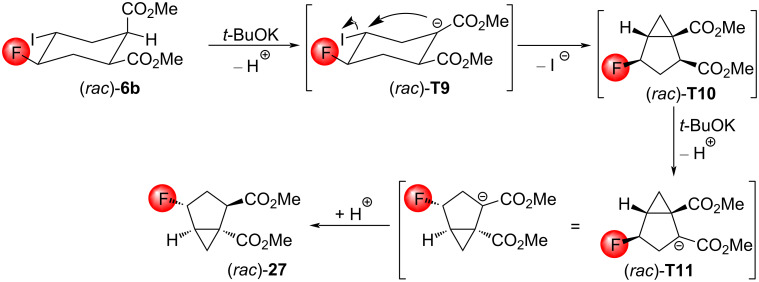
Presumed mechanism of the reaction of the compound (*rac*)*-***6b** with *t*-BuOK.

The reaction of the compound (*rac*)*-***6b** is less direct. According to the above pathway, the formation of the compound (*rac*)*-***T10** would be expected (Scheme 17). In order to generate the final product (*rac*)*-***27**, a base-catalyzed epimerization is required. The requirement of additional reaction steps may explain the lower yield (31%) of this reaction. Note, that from the other three substrates, the product (*rac*)*-***27** was obtained in a yield of 52–54%. Since the enolate (*rac*)*-***T9** is all-equatorial, and consequently, the energy is lower than that of the corresponding stereoisomers, it is more likely that the epimerization occurs after the formation of (*rac*)*-***T10**.

**Scheme 18:**
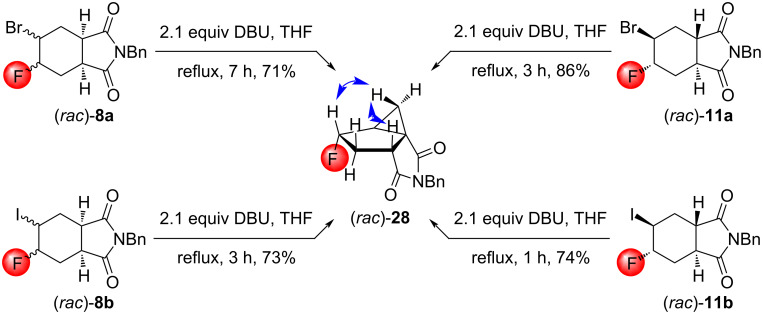
Cyclizations of halofluorinated tetrahydrophthalimides with DBU. Relevant NOESY interactions are marked with two-headed arrows.

Concerning (*rac*)*-***8a** and **8b** as well as (*rac*)*-***11a** and **11b**, the reagent preference was exactly the opposite: DBU in THF under reflux was effective, while *t*-BuOK in THF under reflux was inferior. Interestingly, the same type of tricyclic compound (*rac*)*-***28** was formed from all four substrates (Scheme 18). The stereochemistry of the product was determined using NOESY measurements.

**Scheme 19:**
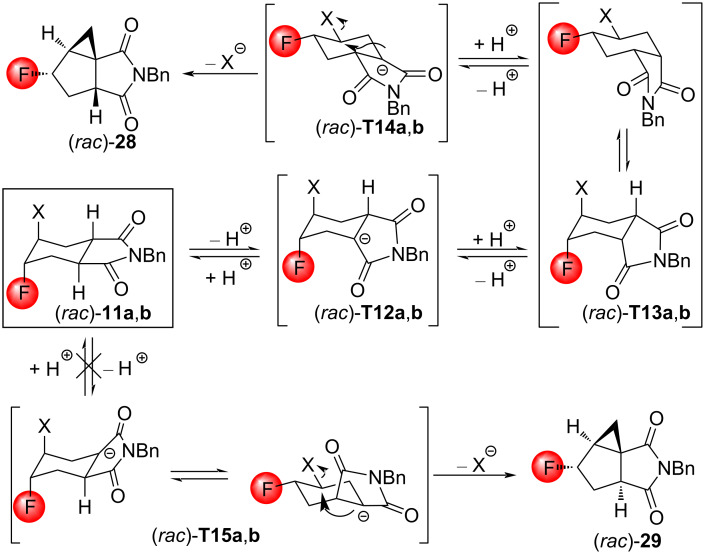
Mechanism for the formation of (*rac*)*-***28** from (*rac*)*-***11a**,**b**. Although the formation of the compound (*rac*)-**29** is theoretically also possible, this product was not observed. X = Br (compounds a), I (compounds b).

Since the structures of the substances in the mixture of (*rac*)*-***8a**,**b** are unknown, a possible mechanism for the formation can be written only from the imides (*rac*)*-***11a**,**b**. Apparently, the first step involves a base-catalyzed epimerization into a less-strained *cis*-annelated system, which undergoes ring inversion, enabling the large halogen atom to be equatorial. This is followed by a deprotonation next to the carbonyl group of the imide ring closer to the CHX moiety. Finally, an intramolecular nucleophilic attack of the carbanion on the CHX motif closes the cyclopropane ring. Of the two possible pathways, only one is observed, which starts with the removal of the sterically less-hindered proton and ends with (*rac*)*-***28**, containing *cis*-annulated cyclopentane rings. Note, that the cyclopentane rings in (*rac*)*-***29** have a more strained *trans*-annulated attachment (Scheme 19).

After finishing the study of the halofluorinations with NBS/Deoxo-Fluor^®^ and NIS/Deoxo-Fluor^®^ systems, our attention shifted to the utilization of Deoxo-Fluor^®^ in fluoroselenations. PhSeBr was selected to provide the phenylselenyl cation serving as the required electrophilic selenium species. This reagent combination was previously unknown in the literature. Preliminary experiments with the diester **4** demonstrated that the reaction is much more efficient in anhydrous CH_3_CN than in anhydrous CH_2_Cl_2_; therefore, the reactions were conducted in this medium at room temperature.

**Scheme 20:**
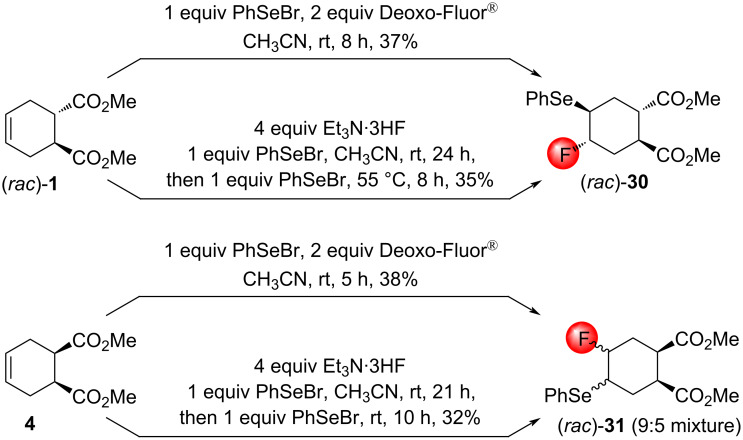
Fluoroselenations of the cyclohexenedicarboxylates (*rac*)*-***1** and **4**.

Similar to halofluorinations, the treatment of the diester (*rac*)**-1** with PhSeBr/Deoxo-Fluor^®^ gave a single product (Scheme 20). The stereochemistry of the cyclohexane (*rac*)*-***30** was determined using NOESY analysis. From the diester **4**, however, a mixture of two fluoroselenides was formed, and the separation failed. The use of PhSeBr/Et_3_N⋅3HF for the fluoroselenation – another reagent combination that was previously unknown in the literature – gave similar results, although these reactions required twice as much PhSeBr (Scheme 20).

Using the above conditions (1 equiv PhSeBr, 2 equiv Deoxo-Fluor^®^, CH_3_CN, rt), the *N*-benzylated *cis*-tetrahydrophthalic imide **7** gave a multicomponent mixture, and no fluoroselenated product was isolated. From the stereoisomeric imide (*rac*)*-***10** and the bicyclic lactam (*rac*)*-***14**, the formation of fluoroselenated products in low amounts could be detected, but the isolation in a pure form failed.

**Scheme 21:**
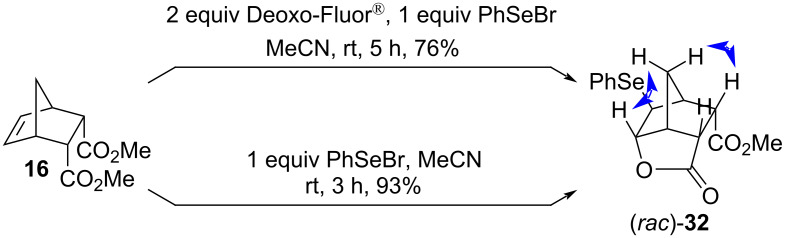
PhSe^+^-induced lactonization of the diester **16**. Relevant NOESY interactions are marked with two-headed arrows.

The treatment of the diester **16** with PhSeBr caused lactonization regardless of the presence or absence of Deoxo-Fluor^®^. The yield was better when phenylselenyl bromide was used alone (Scheme 21). The stereochemistry of the product (*rac*)*-***32** was determined using NOESY data.

The tricyclic imides **19** and **24** failed to react with PhSeBr/Deoxo-Fluor^®^, and only unreacted starting compounds could be recovered from the reaction mixtures. Since these imides showed a decreased reactivity towards the larger I^+^ cation in comparison to the smaller Br^+^ cation (see Scheme 12 and Scheme 14), the absolute lack of reactivity with the even larger PhSe^+^ cation is not surprising.

To study the synthetic utility of the obtained fluoroselenides, oxidative elimination of the phenylselenyl group was attempted. In the case of (*rac*)*-***32**, this reaction would lead to a bridgehead olefin, violating Bredt’s rule. Consequently, only (*rac*)*-***30** and (*rac*)*-***31** were used as starting compounds. On the basis of literature data [20,41–42], we expected the preferred formation of allylic fluorides over fluoroalkenes. Note, that fluoroselenation and subsequent oxidative deselenation can be performed efficiently as a one-pot procedure to transform an olefin directly into an allylic fluoride [41–42].

**Scheme 22:**
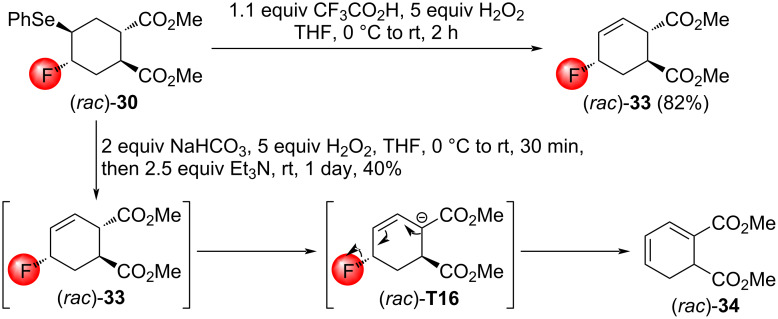
Oxidation of the fluoroselenide (*rac*)*-***30** under acidic and basic conditions.

The oxidation of (*rac*)*-***30** with MCPBA in CH_2_Cl_2_ was ineffective at room temperature and led to decomposition upon reflux. In contrast, the treatment with trifluoroperacetic acid formed in situ in THF resulted in the expected allylic fluoride (*rac*)*-***33**. Oxidative treatment under basic conditions, however, yielded the highly unsaturated diester (*rac*)*-***34**, presumably via a base-promoted E1cB elimination of (*rac*)*-***33** formed primarily (Scheme 22). A possible driving force of this reaction is the extended conjugation.

**Scheme 23:**
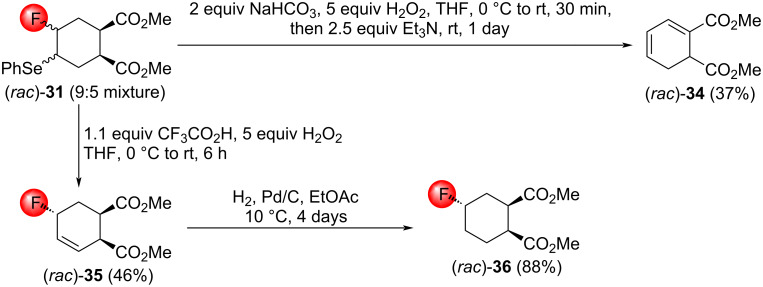
Oxidation of the fluoroselenide mixture (*rac*)*-***31** under acidic and basic conditions.

Interestingly, the treatment of a mixture of (*rac*)*-***31** with H_2_O_2_/CF_3_COOH resulted in single allylic fluoride, (*rac*)*-***35** (Scheme 23). Unfortunately, the determination of the stereochemistry via NOESY was unsuccessful. The reason is that cyclohexenes have a half-chair conformation, and the hydrogen atoms to undergo important NOESY interactions are simply too far away from each other. To solve this problem, the olefin bond of (*rac*)*-***35** was hydrogenated to a cyclohexane, affording the fluorinated dimethyl cyclohexanedicarboxylate (*rac*)*-***36**. The change of the conformation from a half-chair to an ordinary chair enabled the determination of the stereochemistry of (*rac*)*-***36** by NOESY measurements. Since the saturation of (*rac*)*-***35** did not affect the configuration of the CHF motif, that structure was uncovered, too. The oxidation of (*rac*)*-***31** under basic conditions delivered the conjugated diene (*rac*)*-***34**, possibly through a deselenation/E1cB elimination sequence similar to the one shown in Scheme 22.

## Conclusion

In conclusion, novel fluorine-containing, functionalized small-molecular scaffolds have been accessed through halofluorination or selenofluorination protocols. Deoxo-Fluor^®^ proved to be a suitable fluoride source in the halofluorination and fluoroselenation reactions. Bromo- and iodofluorinations were the most useful for symmetric olefins. Regarding cyclohexene derivatives, the extent of selectivity can be predicted by considering the differences of steric shielding on the two sides of the double bond and applying the Fürst–Plattner rule to the halonium ion intermediates. The presence of electron-withdrawing carbonyl groups with α-hydrogen atoms enabled E2 elimination of the halofluorinated products and subsequent cyclization into fused-ring cyclopropanes.

Two new fluoroselenation protocols using PhSeBr/Deoxo-Fluor^®^ and PhSeBr/Et_3_N⋅3HF, respectively, have been described, but the substrate scope of this reaction was more limited. The transformation of the obtained fluoroselenides into allyl fluorides via oxidation succeeded only in an acidic environment. Presumably, the presence of ester groups enabled the base-promoted E1cB elimination, driven by the extension of the conjugation. Further studies of other functionalized cyclic olefin substrates possessing varied architectural structures, and fine-tuning the reaction conditions of other halofluorination and fluoroselenation reactions are currently being studied in our research group.

## Supporting Information

File 1 Characterization and NMR data of the new compounds.

## References

[1] Purser S, Moore P R, Swallow S, Gouverneur V (2008). Chem Soc Rev.

[2] Ojima I (2009). Fluorine in Medicinal Chemistry and Chemical Biology.

[3] Wang J, Sánchez-Roselló M, Aceña J L, del Pozo C, Sorochinsky A E, Fustero S, Soloshonok V A, Liu H (2014). Chem Rev.

[4] Zhou Y, Wang J, Gu Z, Wang S, Zhu W, Aceña J L, Soloshonok V A, Izawa K, Liu H (2016). Chem Rev.

[5] O'Hagan D, Al-Maharik D (2011). Aldrichimica Acta.

[6] Böhm H-J, Banner D, Bendels S, Kansy M, Kuhn B, Müller K, Obst-Sander U, Stahl M (2004). ChemBioChem.

[7] Müller K, Faeh C, Diederich F (2007). Science.

[8] O’Hagan D (2010). J Fluorine Chem.

[9] Hagmann W K (2008). J Med Chem.

[10] Kirsch P (2004). Modern Fluoroorganic Chemistry: Synthesis, Reactivity, Applications.

[11] Liang T, Neumann C N, Ritter T (2013). Angew Chem, Int Ed.

[12] Fustero S, Sedgwick D M, Román R, Barrio P (2018). Chem Commun.

[13] Bykova T, Al-Maharik N, Slawin A M Z, Bühl M, Lebl T, O'Hagan D (2018). Chem – Eur J.

[14] Zhu W, Wang J, Wang S, Gu Z, Aceña J L, Izawa K, Liu H, Soloshonok V A (2014). J Fluorine Chem.

[15] Ishida S, Sheppard T, Nishikata T (2018). Tetrahedron Lett.

[16] Jaroschik F (2018). Chem – Eur J.

[17] Yerien D E, Bonesi S, Postigo A (2016). Org Biomol Chem.

[18] Dykstra K D, Ichiishi N, Krska S W, Richardson P F, Haufe G, Leroux F G (2019). Emerging fluorination methods in organic chemistry relevant for life science application. Fluorine in Life Sciences: Pharmaceuticals, Medicinal Diagnostics, and Agrochemicals.

[19] Marciniak B, Walkowiak-Kulikowska J, Koroniak H (2017). J Fluorine Chem.

[20] Haufe G, Percy J M (2006). Synthesis by addition to alkenes. Fluorine.

[21] Kiss L, Forró E, Fustero S, Fülöp F (2011). Org Biomol Chem.

[22] Kiss L, Forró E, Fustero S, Fülöp F (2011). Eur J Org Chem.

[23] Kiss L, Nonn M, Sillanpää R, Fustero S, Fülöp F (2013). Beilstein J Org Chem.

[24] Kiss L, Nonn M, Forró E, Sillanpää R, Fustero S, Fülöp F (2014). Eur J Org Chem.

[25] Nonn M, Kiss L, Hänninen M M, Sillanpää R, Fülöp F (2012). Chem Biodiversity.

[26] Kiss L, Nonn M, Sillanpää R, Haukka M, Fustero S, Fülöp F (2016). Chem – Asian J.

[27] Kiss L, Remete A M, Nonn M, Fustero S, Sillanpää R, Fülöp F (2016). Tetrahedron.

[28] Kiss L, Petrovszki Á, Vass C, Nonn M, Sillanpää R, Haukka M, Fustero S, Fülöp F (2017). ChemistrySelect.

[29] Remete A M, Nonn M, Fustero S, Haukka M, Fülöp F, Kiss L (2017). Beilstein J Org Chem.

[30] Remete A M, Nonn M, Fustero S, Haukka M, Fülöp F, Kiss L (2018). Eur J Org Chem.

[31] Nonn M, Kiss L, Haukka M, Fustero S, Fülöp F (2015). Org Lett.

[32] Remete A M, Nonn M, Fustero S, Fülöp F, Kiss L (2016). Molecules.

[33] Bohlmann R (1994). Tetrahedron Lett.

[34] Bernáth G, Stájer G, Szabó A E, Fölöp F, Sohár P (1985). Tetrahedron.

[35] Camm K D, Martinez Castro N, Liu Y, Czechura P, Snelgrove J L, Fogg D E (2007). J Am Chem Soc.

[36] Adams J L, Meek T D, Mong S-M, Johnson R K, Metcalf B W (1988). J Med Chem.

[37] Windmon N, Dragojlovic V (2008). Beilstein J Org Chem.

[38] Gültekin D D, Taşkesenligil Y, Daştan A, Balci M (2008). Tetrahedron.

[39] Faith W C, Booth C A, Foxman B M, Snider B B (1985). J Org Chem.

[40] Crandall J K (1964). J Org Chem.

[41] Bloom S, Knippel J L, Holl M G, Barber R, Lectka T (2014). Tetrahedron Lett.

[42] Guo R, Huang J, Zhao X (2018). ACS Catal.

