# Supplementary material for: Synthesis of novel fluorinated building blocks via halofluorination and related reactions
Source: Beilstein J Org Chem. 2020 Oct 16;16:2562–75. doi: 10.3762/bjoc.16.208 (PMC7590628; doi:10.3762/bjoc.16.208)

BEILSTEIN JOURNAL OF ORGANIC CHEMISTRY

Supporting Information

for

Synthesis of novel fluorinated building blocks via halofluorination and related reactions

Attila Márió Remete, Tamás T. Novák, Melinda Nonn, Matti Haukka, Ferenc Fülöp and Loránd Kiss

Beilstein J. Org. Chem. **2020,** *16,* 2562–2575. doi:10.3762/bjoc.16.208

Characterization and NMR data of the new compounds

License and Terms: This is a supporting information file under the terms of the Creative Commons Attribution License (https://creativecommons.org/licenses/by/4.0). Please note that the reuse, redistribution and reproduction in particular requires that the authors and source are credited.
The license is subject to the *Beilstein Journal of Organic Chemistry* terms and conditions: (https://www.beilstein-journals.org/bjoc)

Contents:

Experimental S2

Characterization data S7

X-ray structure determination S24

Copies of ^{1}H and ^{13}C NMR spectra S26

---------------Experimental---------------

General information:

Chemicals were purchased from Sigma-Aldrich. Solvents were used as received from the suppliers. Melting points were determined with a Kofler apparatus. Elemental analyses were carried out with a Perkin-Elmer CHNS-2400 Ser II elemental analyzer. Silica gel 60 F_{254} was purchased from Merck. NMR spectra were acquired at room temperature on a Bruker Avance Neo 500 spectrometer with 11.75 T magnetic field strength (^{1}H frequency 500.20 MHz, ^{19}F frequency 470.66 MHz, ^{13}C frequency 125.78 MHz) in $CDCl_3$ or DMSO-d_6 solution, using the deuterium signal of the solvent to lock the field. The ^{1}H and ^{13}C chemical shifts are given relative to TMS and ^{19}F to $CFCl_3$ (0.00 ppm).

General procedures for the halofluorination:

Method A: A solution of 1.00 mmol of the starting olefin in 10 mL anhydrous CH_2Cl_2 was cooled to 0 °C. Then, 2 equiv Deoxo-Fluor® and 1 equiv *N*-halosuccinimide were added. The reaction mixture was allowed to warm up to rt and stirred for the time given in the Schemes. Then, it was diluted with 20 mL CH_2Cl_2 and washed with 3×10 mL saturated aqueous NaCl solution. The organic layer was dried (Na_2SO_4) and concentrated. The crude product was purified by column chromatography on silica gel (*n*-hexane/EtOAc or *n*-hexane/acetone).

Method B: A solution of 1.00 mmol of the starting olefin in 10 mL anhydrous CH_2Cl_2 was cooled to 0 °C. Then, 2 equiv Deoxo-Fluor® and 1 equiv *N*-halosuccinimide were added. The reaction mixture was allowed to warm up to rt and stirred for the time given in the Schemes. Then, 2 equiv Deoxo-Fluor® and 1 equiv *N*-halosuccinimide were added again, and stirring was continued at rt for the time given in the Schemes. Then, the mixture was diluted with 20 mL CH_2Cl_2 and washed with 3×10 mL saturated aqueous NaCl solution. The organic layer was dried (Na_2SO_4) and concentrated. The crude product was purified by column chromatography on silica gel (*n*-hexane/EtOAc or *n*-hexane/acetone).

Method C: Same as *Method B*, except that during the second reagent addition step, only *N*-halosuccinimide (1 equiv) was added.

Synthesis of the *trans* imide (*rac*)-10:

The synthesis of the compound (*rac*)-**10** was analogous to that of the imide **19** except using a shorter reaction time (5 h.) and purification by column chromatography on silica gel (*n*-hexane/EtOAc 9:1) to yield 42% of the product (*rac*)-**10** as a white solid.

Synthesis of the cyclic carbamide (*rac*)-13:

To a solution of 0.30 g (1.62 mmol) *trans*-cyclohex-4-ene-1,2-diamine hydrochloride ((*rac*)-**12**) and 2.2 equiv Et_3N in 8 mL DMF, the solution of 1.35 equiv CDI in 7 mL DMF was added dropwise. The resulting mixture was stirred at rt for 4 days. Then, 1 mL water was added to decompose unreacted CDI, followed by concentration of the solution. The crude product was purified by column chromatography on silica gel (CH_2Cl_2/MeOH 95:5) to obtain the product (*rac*)**-13** in 10% yield as a white solid.

Alternative preparation of the halolactons (*rac*)-17a,b:

Performed analogously to *General procedures for halofluorination, Method A* except without the addition of Deoxo-Fluor®.

Preparation of the *N*-methyl imide 19:

Compound **19** was synthesized according to Reference RS1.

Bromofluorination of the *N*-benzyl imide 19 under reflux:

To a solution of 1.00 mmol of the starting olefin in 14 mL anhydrous CH_2Cl_2, 2 equiv Deoxo-Fluor® and 1 equiv *N*-bromosuccinimide were added. The reaction mixture was kept at reflux temperature for 7 h. Then, 1 equiv NBS was added again, and the mixture was heated further under reflux for an additional 7 h. Then, the mixture was diluted with 20 mL CH_2Cl_2 and washed with 3×10 mL saturated aqueous NaCl solution. The organic layer was dried (Na_2SO_4) and concentrated. The crude product was purified by column chromatography on silica gel with gradient elution (*n*-hexane/acetone 15:1→13:1→10:1) to yield 69% of the product **22** as a pale yellowish brown solid.

Preparation of the *N*-methyl imide 24:

Compound **24** was synthesized by a slightly modified version of the method used to obtain the imide **19**. 3.00 g (18.27 mmol) of the tricyclic anhydride **23** was dissolved in 80 mL toluene. Then, 1 equiv $MeNH_3Cl$, 1.2 equiv Et_3N, and 1 equiv $NaHCO_3$ were added. Since $MeNH_2$ is

volatile, the mixture was stirred at rt for 18 h to allow the half-amide intermediate to form. Then, the mixture was treated at reflux temperature for 15 h to facilitate ring-closing. After cooling, it was washed with 3×40 mL 10% aqueous HCl solution, and then with 3×30 mL saturated aqueous NaCl solution. The organic layer was dried (Na_2SO_4), slightly concentrated, and titurated with hexane to obtain the product **24** as a white solid (yield of 50%).

Synthesis of the bicyclic cyclopropane (*rac*)-27:

To a solution of 0.30 mmol of the starting compound in 15 mL THF, 1 equiv *t*-BuOK was added. The reaction mixture was heated under reflux for the time given in the Scheme. Then, for the compound (*rac*)-**2a** only, another equivalent of *t*-BuOK was added, and reflux treatment was continued for 1 h. After cooling, the reaction mixture was diluted with 25 mL EtOAc and washed with 2×15 mL water. The organic layer was dried (Na_2SO_4) and concentrated. The crude product was purified by column chromatography on silica gel. The eluent depended on the starting compound ((*rac*)-**2a**: *n*-hexane/acetone 18:1 → 15:1, (*rac*)-**2b**: *n*-hexane/acetone 18:1 → 17:1, (*rac*)-**5a**: *n*-hexane/EtOAc 8:1 → 6:1, (*rac*)-**6b**: *n*-hexane/EtOAc 7:1 → 5:1).

Synthesis of the tricyclic cyclopropane (*rac*)-28:

To a solution of 0.29 mmol of the starting compound in 10 mL THF, 2.1 equiv DBU was added. The reaction mixture was kept at reflux temperature for the time given in the Scheme. Then, it was cooled, diluted with 25 mL EtOAc, and washed with 3×15 mL water. The organic layer was dried (Na_2SO_4) and concentrated. The crude product was purified by column chromatography on silica gel. The eluent depended on the starting compound ((*rac*)-**8a**: *n*-hexane/EtOAc 5:3, (*rac*)-**8b**: *n*-hexane/acetone 6:1, (*rac*)-**11a**: *n*-hexane/EtOAc 3:1 → 1:1, (*rac*)-**11b**: *n*-hexane/EtOAc 3:2).

General procedure for the fluoroselenation:

To a solution of 1.00 mmol of the starting olefin in 10 mL anhydrous MeCN, 2 equiv Deoxo-Fluor® and 1 equiv PhSeBr were added. The reaction mixture was stirred at rt for the time given in the Scheme. Then, it was diluted with 20 mL EtOAc and washed with 2×10 mL of a saturated aqueous $NaHCO_3$ solution. The organic layer was dried (Na_2SO_4) and concentrated. The crude product was purified by column chromatography on silica gel (*n*-hexane/EtOAc or *n*-hexane/acetone).

Alternative procedure for the fluoroselenation of the diester (*rac*)-1:

To a solution of 1.00 mmol of dimethyl *trans*-cyclohex-4-ene-1,2-dicarboxylate ((*rac*)-**1**) in 10 mL anhydrous MeCN, 4 equiv $Et_3N \times 3HF$ and 1 equiv PhSeBr were added. The reaction mixture was stirred at rt for 24 h. After adding 1 equivalent of PhSeBr again, the mixture was stirred at 55 °C for further 8 h. After diluting with 20 mL EtOAc, it was washed with 2×10 mL of a saturated aqueous $NaHCO_3$ solution. The organic layer was dried (Na_2SO_4) and concentrated. The crude product was purified by column chromatography on silica gel (*n*-hexane/acetone 18:1) to obtain 35% of the product (*rac*)-**30** as a pale yellow oil.

Alternative procedure for the fluoroselenation of the diester 4:

To a solution of 1.00 mmol of dimethyl *cis*-cyclohex-4-ene-1,2-dicarboxylate (**4**) in 10 mL anhydrous MeCN, 4 equiv $Et_3N \times 3HF$ and 1 equiv PhSeBr were added. The reaction mixture was stirred at rt for 21 h. Then, after the addition of another equivalent of PhSeBr, the mixture was stirred at rt for further 10 h. Next, the mixture was diluted with 20 mL EtOAc and washed with 2×10 mL saturated aqueous $NaHCO_3$ solution. The organic layer was dried (Na_2SO_4) and concentrated. The crude product was purified by column chromatography on silica gel (*n*-hexane/acetone 17:1) to obtain 32% of the product (*rac*)-**31** as a pale brown oil. Notably, the purification of this mixture was easier than the purification of reaction mixtures obtained via the general fluoroselenation procedure.

Alternative preparation of the lactone (*rac*)-32:

To a solution of 1.00 mmol of the starting compound **16** in 10 mL anhydrous MeCN, 1 equiv PhSeBr was added, and the reaction mixture was stirred at rt for the time given in the Scheme. Then, it was diluted with 20 mL EtOAc and washed with 2×10 mL of a saturated aqueous $NaHCO_3$ solution. The organic layer was dried (Na_2SO_4) and concentrated. The crude product was purified by column chromatography on silica gel (*n*-hexane/acetone 6:1) to obtain 93% of the product (*rac*)-**32** as a yellowish solid.

Oxidative elimination of the phenylselenyl group under acidic conditions:

0.32 mmol of the starting compound ((*rac*)-**30** or (*rac*)-**31**) was dissolved in 8 mL THF. After cooling to 0 °C, 5 equiv H_2O_2 (as a 30 wt % aqueous solution) and 1.1 equiv trifluoroacetic acid were added, and the mixture was allowed to warm up to rt upon stirring for the time given in the Scheme. Then, it was diluted with 30 mL EtOAc and washed with 2×10 mL of a saturated aqueous $NaHCO_3$ solution. The organic layer was dried (Na_2SO_4) and concentrated. The crude

product was purified by column chromatography on silica gel ((*rac*)-**30**: *n*-hexane/EtOAc 6:1, (*rac*)-**31**: *n*-hexane/EtOAc 9:1).

Oxidative elimination of the phenylselenyl group under basic conditions:

0.33 mmol of the starting compound ((*rac*)-**30** or (*rac*)-**31**) was dissolved in 8 mL THF. After cooling to 0 °C, 2 equiv $NaHCO_3$ and 5 equiv H_2O_2 (as 30 wt % aqueous solution) were added and the mixture was stirred at 0 °C for 30 min. Then 2.5 equiv Et_3N was added and the mixture was allowed to warm up to rt while it was stirred for a day. Then, it was diluted with 30 mL EtOAc and washed with 2×10 mL water. The organic layer was dried (Na_2SO_4) and concentrated. The crude product was purified by column chromatography on silica gel (*n*-hexane/EtOAc 9:1 → 7:1).

Hydrogenation of (*rac*)-35:

To a solution of 0.25 mmol (*rac*)-**35** in 5 mL EtOAc, 15 mg Pd/C (10 wt %) was added, and the reaction mixture was stirred at 10 °C under a hydrogen atmosphere (1 bar) for 4 days. The reaction mixture was then filtered on a fritted glass filter covered with Celite®. The filtrate was dried (Na_2SO_4) and concentrated. The crude product was purified by column chromatography on silica gel (*n*-hexane/EtOAc 5:1) to obtain 88% of the product (*rac*)-**36** as a colorless oil.

---------------**Characterization data**---------------

(1*S,2*S**,4*S**,5*S**)-Dimethyl 4-bromo-5-fluorocyclohexane-1,2-dicarboxylate;** (*rac*)-**2a**

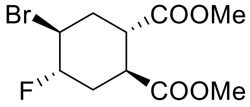

Prepared according to *General procedures for halofluorination, Method A* (eluent for column chromatography: *n*-hexane/acetone 17:1). Colorless oil; yield 58%.

R_f = 0.43 (*n*-hexane/acetone 7:1).

^{1}H-NMR (500 MHz, $CDCl_3$, TMS): δ (ppm) = 2.19-2.41 (m, 4H, H-3 and H-6), 3.00-3.10 (m, 1H, H-1), 3.12-3.21 (m, 1H, H-2), 3.71 (s, 3H, OCH_3), 3.72 (s, 3H, OCH_3), 4.34-4.41 (m, 1H, H-4), 4.81-4.95 (m, 1H, H-5).

^{19}F NMR (471 MHz, $CDCl_3$): δ (ppm) = -169.97.

^{13}C NMR (126 MHz, $CDCl_3$, TMS): δ (ppm) = 27.7 and 27.8 (^2J=20.87 Hz), 31.0, 39.0 and 39.0 (^3J=1.35 Hz), 39.2, 45.8 and 46.0 (^2J=26.75 Hz), 52.2, 52.2, 88.1 and 89.5 (^1J=177.10 Hz), 174.0, 174.0.

MS (ESI) m/z = 297 [M+1], 299 [M+3].

HRMS calcd. for $C_{10}H_{15}BrFO_4^+$ ($[M+H]^+$): 297.0132 (^{79}Br), 299.0112 (^{81}Br). Found: 297.0134 (^{79}Br), 299.0111 (^{81}Br).

(1*S,2*S**,4*S**,5*S**)-Dimethyl 4-fluoro-5-iodocyclohexane-1,2-dicarboxylate;** (*rac*)-**2b**

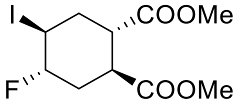

Prepared according to *General procedures for halofluorination, Method A* (eluent for column chromatography: *n*-hexane/acetone 17:1). Yellow oil; yield 56%. R_f = 0.35 (*n*-hexane/acetone 7:1).

^{1}H-NMR (500 MHz, $CDCl_3$, TMS): δ (ppm) = 2.20-2.33 (m, 3H, H-6 and H-3), 2.38-2.55 (m, 1H, H-3), 3.05-3.17 (m, 2H, H-1 and H-2), 3.71 (s, 3H, OCH_3), 3.72 (s, 3H, OCH_3), 4.48-4.55 (m, 1H, H-5), 4.88-5.02 (m, 1H, H-4).

^{19}F NMR (471 MHz, $CDCl_3$): δ (ppm) = -163.4.

^{13}C NMR (126 MHz, $CDCl_3$, TMS): δ (ppm) = 24.6 and 24.8 (^2J=23.07 Hz), 28.0 and 28.1 (^2J=20.29 Hz), 32.3, 39.3 and 39.3 (^3J=1.50 Hz), 40.6, 52.2, 52.2, 89.4 and 90.8 (^1J=178.00 Hz), 173.9, 174.0.

MS (ESI) m/z = 345 [M+1]

HRMS calcd. for $C_{10}H_{14}FINaO_4^+$ ($[M+Na]^+$): 366.9813, found: 366.9810. Calcd. for $C_{10}H_{15}FIO_4^+$ ($[M+H]^+$): 344.9994, found: 344.9994.

(1*R,2*S**,4*R**,5*R**)-Dimethyl 4-bromo-5-fluorocyclohexane-1,2-dicarboxylate;** (*rac*)-**5a**

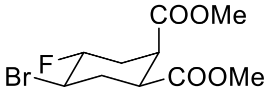

Prepared according to *General procedures for halofluorination, Method A* (eluent for column chromatography: *n*-hexane/EtOAc 5:1). White solid; yield 32%. R_f = 0.31 (*n*-hexane/EtOAc 4:1), mp. 49-58 °C.

^{1}H-NMR (500 MHz, $CDCl_3$, TMS): δ (ppm) = 1.90-2.03 (m, 1H, H-6), 2.45-2.56 (m, 1H, H-3), 2.61-2.73 (m, 2H, H-3 and H-6), 2.74-2.85 (m, 1H, H-2), 3.18-3.28 (m, 1H, H-1), 3.71 (s, 3H, OCH_3), 3.72 (s, 3H, OCH_3), 3.97-4.07 (m, 1H, H-4), 4.63-4.81 (m, 1H, H-5).

^{19}F NMR (476 MHz, $CDCl_3$): δ (ppm) = -173.35.

^{13}C NMR (126 MHz, $CDCl_3$, TMS): δ (ppm) = 29.7, 32.4, 39.5, 41.8, 48.3, 52.2, 52.3, 90.4 and 91.8 (^1J=179.19 Hz), 171.9, 172.5.

HRMS calcd. for $C_{10}H_{15}BrFO_4^+$ ($[M+H]^+$): 297.0132 (^{79}Br), 299.0112 (^{81}Br). Found: 297.0134 (^{79}Br), 299.0110 (^{81}Br).

(1*R,2*S**,4*R**,5*R**)-Dimethyl 4-fluoro-5-iodocyclohexane-1,2-dicarboxylate;** (*rac*)-**5b**

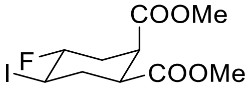

Prepared according to *General procedures for halofluorination, Method A* (eluent gradient for column chromatography: *n*-hexane/EtOAc 7:1→5:1→4:1). White solid; yield 10%. R_f = 0.36 (*n*-hexane/EtOAc 4:1), mp. 73-80 °C.

^{1}H-NMR (500 MHz, $CDCl_3$, TMS): δ (ppm) = 1.82-1.94 (m, 1H, H-3), 2.54-2.78 (m, 4H, H-1, H-3, H-6), 3.30-3.37 (m, 1H, H-2), 3.71 (s, 3H, OCH_3), 3.72 (s, 3H, OCH_3), 3.99-4.08 (m, 1H, H-5), 4.62-4.80 (m, 1H, H-4).

^{19}F NMR (476 MHz, $CDCl_3$): δ (ppm) = -166.18.

^{13}C NMR (126 MHz, $CDCl_3$, TMS): δ (ppm) = 25.4, 29.7, 34.7, 40.3, 43.5, 52.3, 52.3, 91.5 and 93.0 (^1J=179.57 Hz), 171.4, 172.5.

MS (ESI) m/z = 345 [M+1]

HRMS calcd. for $C_{10}H_{15}FIO_4^+$ ($[M+H]^+$): 344.9994. Found: 344.9994.

(1*S,2*R**,4*R**,5*R**)-Dimethyl 4-fluoro-5-iodocyclohexane-1,2-dicarboxylate;** (*rac*)**-6b**

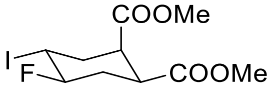

Prepared according to *General procedures for halofluorination, Method A* (eluent gradient for column chromatography: *n*-hexane/EtOAc 7:1→5:1→4:1). Yellow oil; yield 42%. R_f = 0.40 (*n*-hexane/EtOAc 3:1).

^{1}H-NMR (500 MHz, $CDCl_3$, TMS): δ (ppm) = 2.21-2.30 (m, 1H, H-6), 2.31-2.43 (m, 1H, H-3), 2.50-2.63 (m, 1H, H-3), 2.67-2.78 (m, 1H, H-6), 2.91-2.98 (m, 1H, H-2), 2.98-3.05 (m, 1H, H-1), 3.69 (s, 1H, OCH_3), 3.72 (s, 1H, OCH_3), 4.28-4.39 (m, 1H, H-5), 4.61-4.78 (m, 1H, H-4).

^{19}F NMR (476 MHz, $CDCl_3$): δ (ppm) = -162.40.

^{13}C NMR (126 MHz, $CDCl_3$, TMS): δ (ppm) = 26.0 and 26.1 (^2J=21.36 Hz), 29.2 and 29.4 (^2J=20.94 Hz), 33.5, 39.7, 40.9, 52.1, 52.2, 91.5 and 92.9 (^1J=179.80 Hz), 172.4, 172.5.

MS (ESI) m/z = 345 [M+1]

HRMS calcd. for $C_{10}H_{14}FINaO_4^+$ ($[M+Na]^+$): 366.9813, found: 366.9810. Calcd. for $C_{10}H_{15}FIO_4^+$ ($[M+H]^+$): 344.9994, found: 344.9994.

(3a*R,7a*S**)-2-Benzyl-5-bromo-6-fluorohexahydro-1*H*-isoindole-1,3(2*H*)-dione;** (*rac*)**-8a**

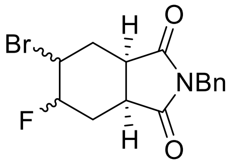

Prepared according to *General procedures for halofluorination, Method A* (eluent gradient for column chromatography: *n*-hexane/acetone 6:1→5:1). White solid; yield 66% (7:5 mixture of 2 diastereomers).

(3aS*,7aR*)-2-Benzyl-5-fluoro-6-iodohexahydro-1*H*-isoindole-1,3(2*H*)-dione; (*rac*)**-8b**

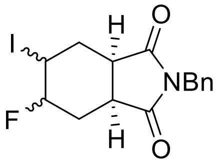

Prepared according to *General procedures for halofluorination, Method A* (eluent for column chromatography: *n*-hexane/acetone 9:1). White solid; yield 61% (2:1 mixture of 2 diastereomers).

(3a*S,7a*S**)-2-Benzyl-3a,4,7,7a-tetrahydro-1*H*-isoindole-1,3(2*H*)-dione;** (*rac*)-**10**

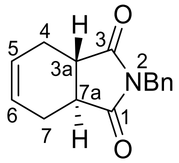

White solid; yield 42%. R_f = 0.47 (*n*-hexane/EtOAc 4:1), mp. 79-83 °C.

^{1}H-NMR (500 MHz, $CDCl_3$, TMS): δ (ppm) = 2.25-2.36 (m, 2H, H-4 and H-7), 2.51-2.63 (m, 4H, H-3a, H-4, H-7 and H-7a), 4.58-4.68 (m, 2H, benzylic CH_2), 5.76-5.82 (m, 2H, H-5 and H-6), 7.27-7.39 (m, 5H, Ph).

^{13}C NMR (126 MHz, $CDCl_3$, TMS): δ (ppm) = 25.4, 41.8, 43.4, 126.7, 127.9, 128.7, 128.8, 136.2, 176.5.

MS (ESI) m/z = 242 [M+1]

HRMS calcd. for $C_{15}H_{16}NO_2^+$ ($[M+H]^+$): 242.1176, found: 242.1172.

(3a*S,5*S**,6*S**,7a*S**)-2-Benzyl-5-bromo-6-fluorohexahydro-1*H*-isoindole-1,3(2*H*)-dione;** (*rac*)-**11a**

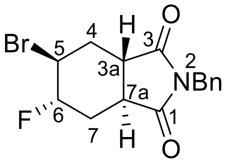

Prepared according to *General procedures for halofluorination, Method A* (eluent gradient for column chromatography: *n*-hexane/EtOAc 10:1→9:1). White solid; yield 67%. R_f = 0.53 (*n*-hexane/EtOAc 4:1), mp. 71-78 °C.

^{1}H-NMR (500 MHz, $CDCl_3$, TMS): δ (ppm) = 2.21-2.38 (m, 2H, H-4 and H-7), 2.48-2.59 (m, 2H, H-4 and H-7), 2.74-2.82 (m, 1H, H-7a), 2.97-3.05 (m, 1H, H-3a), 4.48-4.54 (m, 1H, H-5), 4.57-4.68 (m, 2H, benzylic CH_2), 4.94-5.07 (m, 1H, H-6), 7.27-7.39 (m, 5H, Ph).

^{19}F NMR (471 MHz, $CDCl_3$): δ (ppm) = -167.14

^{13}C NMR (126 MHz, $CDCl_3$, TMS): δ (ppm) = 25.5 and 25.6 (^2J=19.40 Hz), 29.0, 40.9 and 40.9 (^3J=1.40 Hz), 41.0, 42.0, 46.4 and 46.7 (^2J=29.39 Hz), 88.9 and 90.3 (^1J=178.35 Hz), 128.0, 128.7, 128.8, 135.9, 175.2, 175.3.

MS (ESI) m/z = 340 [M+1], 342 [M+3]

HRMS calcd. for $C_{15}H_{16}BrFNO_2^+$ ($[M+H]^+$): 340.0343 (^{79}Br), 342.0322 (^{81}Br). Found: 340.0344 (^{79}Br), 342.0322 (^{81}Br).

(3a*S,5*S**,6*S**,7a*S**)-2-Benzyl-5-fluoro-6-iodohexahydro-1*H*-isoindole-1,3(2*H*)-dione; (*rac*)-11b**

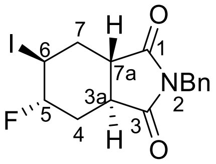

Prepared according to *General procedures for halofluorination, Method A* (eluent for column chromatography: *n*-hexane/EtOAc 10:1). White solid; yield 61%. R_f = 0.45 (*n*-hexane/EtOAc 4:1), mp. 129-132 °C.

^{1}H-NMR (500 MHz, $CDCl_3$, TMS): δ (ppm) = 2.13-2.22 (m, 1H, H-7), 2.38-2.59 (m, 3H, H-4 and H-7), 2.75-2.82 (m, 1H, H-3a), 2.95-3.03 (m, 1H, H-7a), 4.58-4.70 (m, 3H, H-6 and benzylic CH_2), 5.01-5.14 (m, 1H, H-5), 7.26-7.39 (m, 5H, Ph).

^{19}F NMR (471 MHz, $CDCl_3$): δ (ppm) = -159.06.

^{13}C NMR (126 MHz, $CDCl_3$, TMS): δ (ppm) = 25.5 and 25.7 (^2J=19.65 Hz), 25.6 and 25.8 (^2J=25.18 Hz), 29.9, 41.2 and 41.2 (^3J=1.32 Hz), 42.0, 42.4, 90.3 and 91.7 (^1J=179.85 Hz), 128.0, 128.7, 128.8, 135.9, 175.2, 175.3.

MS (ESI) m/z = 388 [M+1]

HRMS calcd. for $C_{15}H_{16}FINO_2^+$ ($[M+H]^+$): 388.0204, found: 388.0203.

(3a*S,7a*S**)-3a,4,7,7a-Tetrahydro-1*H*-benzo[d]imidazol-2(3*H*)-one; (*rac*)-13**

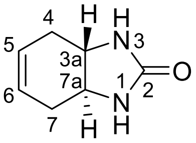

White solid; yield 10%. R_f = 0.73 (CH_2Cl_2/MeOH 9:1), mp. 222-224 °C

^{1}H-NMR (500 MHz, $CDCl_3$, TMS): δ (ppm) = 2.12-2.24 (m, 2H, H-4 and H-7), 2.34-2.46 (m, 2H, H-4 and H-7), 3.34-3.44 (m, 2H, H-3a and H-7a), 4.79 (s, 2H, NH), 5.65-5.71 (m, 2H, H-5 and H-6).

^{13}C NMR (126 MHz, $CDCl_3$, TMS): δ (ppm) = 30.9, 56.8, 125.1, 164.8.

HRMS calcd. for $C_7H_{11}N_2O^+$ ($[M+H]^+$): 139.0866, found: 139.0862.

tert-Butyl (1_S_*,4_S_*,5_R_*,6_R_*)-6-bromo-5-fluoro-3-oxo-2-azabicyclo[2.2.1]heptane-2-carboxylate; (_rac_)-15a

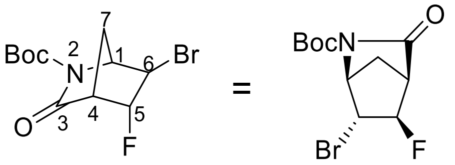

Prepared according to *General procedures for halofluorination, Method C* (eluent for column chromatography: *n*-hexane/EtOAc 6:1→4:1; a subsequent crystallization was also necessary to obtain pure product). White solid; yield 35%. R_f = 0.35 (*n*-hexane/EtOAc 4:1), mp. 124-128 °C.

^{1}H-NMR (500 MHz, $CDCl_3$, TMS): δ (ppm) = 1.54 (s, 9H, Boc), 2.20-2.27 (m, 1H, H-7), 2.28-2.35 (m, 1H, H-7), 3.19-3.24 (m, 1H, H-4), 4.04-4.12 (m, 1H, H-6), 4.54-4.58 (m, 1H, H-1), 5.42-5.57 (m, 1H, H-5).

^{19}F NMR (471 MHz, $CDCl_3$): δ (ppm) = -177.25.

^{13}C NMR (126 MHz, $CDCl_3$, TMS): δ (ppm) = 28.0, 34.1 and 34.2 (^{3}J=4.13 Hz), 48.0 and 48.2 (^{2}J=25.67 Hz), 51.9 and 52.0 (^{2}J=19.55 Hz), 63.2 and 63.2 (^{3}J=1.43 Hz), 84.2, 97.8 and 99.4 (^{1}J=200.94 Hz), 148.7, 168.3 and 168.3 (^{3}J=5.47 Hz).

MS (ESI) m/z = 330 [M+Na], 332 [M+2+Na]

HRMS calcd. for $C_{11}H_{15}BrFNNaO_3^+$ ([M+Na]$^+$): 330.0112 (^{79}Br), 332.0091 (^{81}Br). Found: 330.0112 (^{79}Br), 332.0090 (^{81}Br).

tert-Butyl (1_S_*,4_S_*,5_R_*,6_R_*)-5-fluoro-6-iodo-3-oxo-2-azabicyclo[2.2.1]heptane-2-carboxylate; (_rac_)-15b

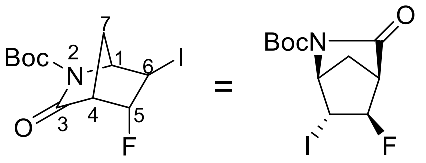

Prepared according to *General procedures for halofluorination, Method C* (eluent for column chromatography: *n*-hexane/EtOAc 4:1). White solid; yield 61%. R_f = 0.31 (*n*-hexane/EtOAc 4:1), mp. 109-117 °C.

^{1}H-NMR (500 MHz, $CDCl_3$, TMS): δ (ppm) = 1.54 (s, 9H, Boc), 2.24-2.31 (m, 1H, H-7), 2.37-2.43 (m, 1H, H-7), 3.13-3.18 (m, 1H, H-4), 4.01-4.09 (m, 1H, H-6), 4.57-4.61 (m, 1H, H-1), 5.57-5.72 (m, 1H, H-5).

^{19}F NMR (471 MHz, $CDCl_3$): δ (ppm) = -173.88.

^{13}C NMR (126 MHz, $CDCl_3$, TMS): δ (ppm) = 22.0 and 22.2 (^{2}J=23.98 Hz), 28.0, 34.7 and 34.8 (^{3}J=3.88 Hz), 52.9 and 53.1 (^{2}J=18.80 Hz), 64.7, 84.1, 99.2 and 100.8 (^{1}J=201.32 Hz), 148.7, 168.0 and 168.0 (^{3}J=5.65 Hz).
MS (ESI) m/z = 378 [M+Na]
HRMS calcd. for $C_{11}H_{15}FINNaO_3^+$ ($[M+Na]^+$): 377.9973, found: 377.9972.

(3*R,3a*R**,5*S**,6*S**,6a*S**,7*R**)-Methyl 6-bromo-2-oxohexahydro-2*H*-3,5-methanocyclopenta[b]furan-7-carboxylate;** (*rac*)-**17a**[RS2]

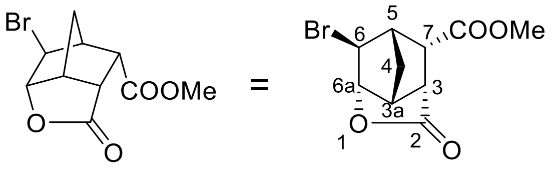

Prepared according to *General procedures for halofluorination, Method A* (yield: 82%) or *Alternative preparation of halolactons* (*rac*)-***17a,b*** (yield: 47%), in both cases the eluent for column chromatography was *n*-hexane/EtOAc 3:1. White solid, R_f = 0.44 (*n*-hexane/EtOAc 3:2), mp. 68-74 °C.
^{1}H-NMR (400 MHz, $CDCl_3$, TMS): δ (ppm) = 1.75-1.81 (m, 1H, CH_2), 2.39-2.46 (m, 1H, CH_2), 2.83 (dd, J=10.81 Hz, J=4.72 Hz, 1H, H-3), 2.87-2.91 (m, 1H, H-5), 3.16 (dd, J=10.81 Hz, J=3.35 Hz, 1H, H-7), 3.31-3.38 (m, 1H, H-3a), 3.74 (s, 3H, OCH_3), 4.58-4.63 (m, 1H, H-6), 4.97-5.03 (m, 1H, H-6a).
^{13}C NMR (100 MHz, $CDCl_3$, TMS): δ (ppm) = 36.1, 41.0, 48.1, 48.5, 49.1, 49.7, 52.8, 87.8, 170.9, 176.5.
MS (ESI) m/z = 275 [M+1], 277 [M+3]
HRMS calcd. for $C_{10}H_{12}BrO_4^+$ ($[M+H]^+$): 274.9913 (^{79}Br), 276.9893 (^{81}Br). Found: 274.9920 (^{79}Br), 276.9897 (^{81}Br).

(3*R,3a*R**,5*S**,6*S**,6a*S**,7*R**)-Methyl 6-iodo-2-oxohexahydro-2*H*-3,5-methanocyclopenta[b]furan-7-carboxylate;** (*rac*)-**17b**[RS2]

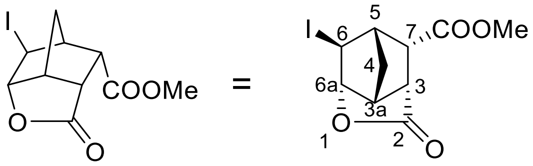

Prepared according to *General procedures for halofluorination, Method A* (yield: 67%) or *Alternative preparation of halolactons* (*rac*)-***17a,b*** (yield: 78%), in both cases the eluent

gradient for column chromatography was *n*-hexane/EtOAc 4:1→2:1. White solid, R_f = 0.29 (*n*-hexane/EtOAc 2:1), mp. 93-94 °C.

^{1}H-NMR (500 MHz, $CDCl_3$, TMS): δ (ppm) = 1.86-1.92 (m, 1H, CH_2), 2.45-2.52 (m, 1H, CH_2), 2.83 (dd, *J*=10.77 Hz, *J*=4.67 Hz, 1H, H-3), 2.91-2.95 (m, 1H, H-5), 3.10 (dd, *J*=10.80 Hz, *J*=3.23 Hz, 1H, H-7), 3.28-3.33 (m, 1H, H-3a), 3.74 (s, 3H, OCH_3), 4.64-4.68 (m, 1H, H-6), 5.18-5.22 (m, 1H, H-6a).

^{13}C NMR (126 MHz, $CDCl_3$, TMS): δ (ppm) = 24.84, 37.62, 40.30, 48.39, 48.70, 49.36, 52.45, 88.75, 170.61, 176.29.

MS (ESI) m/z = 323 [M+1]

HRMS calcd. for $C_{10}H_{12}IO_4^+$ ($[M+H]^+$): 322.9775, found: 322.9777.

(3a*R*,4*S*,7*R*,7a*S*)-2-Benzyl-3a,4,7,7a-tetrahydro-1*H*-4,7-methanoisoindole-1,3(2*H*)-dione; 19

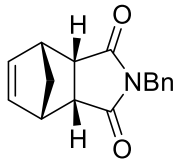

White solid. R_f = 0.45 (*n*-hexane/acetone 3:1), mp. 81-86 °C.

NMR data is identical to the one reported by Camm, K. D.; Castro, N. M.; Liu, Y.; Czechura, P.; Snelgrove, J. L.; Fogg, D. E. *J. Am. Chem. Soc.* **2007**, *129*, 4168–4169.

HRMS calcd. for $C_{16}H_{16}NO_2^+$ ($[M+H]^+$): 254.1176, found: 254.1176.

(3a*R,4*R**,5*R**,7*R**,7a*S**,8*R**)-2-Benzyl-8-bromo-5-fluorohexahydro-1*H*-4,7-methanoisoindole-1,3(2*H*)-dione;** (*rac*)-**20a**

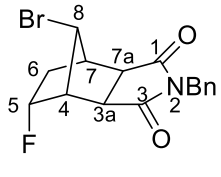

Prepared according to *General procedures for halofluorination, Method B* (eluent gradient for column chromatography: *n*-hexane/acetone 14:1→10:1→8:1). White solid; yield 28%. R_f = 0.50 (*n*-hexane/acetone 3:1), mp. 120-134 °C.

^{1}H-NMR (500 MHz, $CDCl_3$, TMS): δ (ppm) = 1.43-1.56 (m, 1H, H-6), 2.62-2.75 (m, 1H, H-6), 2.80-2.86 (m, 1H, H-7), 2.95 (d, *J*=8.11 Hz, 1H, H-7a), 3.09-3.16 (m, 1H, H-4), 3.33 (d, *J*=8.11 Hz, 1H, H-3a), 3.56-3.62 (m, 1H, H-8), 4.66 (s, 2H, benzylic CH_2), 5.40-5.58 (m, 1H, H-5), 7.27-7.40 (m, 5H, Ph).

^{19}F NMR (471 MHz, $CDCl_3$): δ (ppm) = -197.00.

^{13}C NMR (126 MHz, $CDCl_3$, TMS): δ (ppm) = 34.0 and 34.2 (^{2}J=22.27 Hz), 39.5 and 39.6 (^{3}J=13.23 Hz), 42.9, 45.4, 46.6, 49.2 and 49.4 (^{2}J=26.82 Hz), 49.4 and 49.5 (^{3}J=12.98 Hz), 91.3 and 92.8 (^{1}J=188.15 Hz), 128.3, 128.8, 128.9, 135.5, 176.4, 177.0.

HRMS calcd. for $C_{16}H_{16}BrFNO_2^+$ ($[M+H]^+$): 352.0343 (^{79}Br), 354.0322 (^{81}Br). Found: 352.0342 (^{79}Br), 354.0320 (^{81}Br).

(3a*R,4*R**,5*S**,7*R**,7a*S**,8*R**)-2-Benzyl-8-bromo-5-fluorohexahydro-1*H*-4,7-methanoisoindole-1,3(2*H*)-dione;** (*rac*)-**21a**

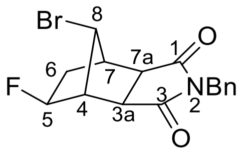

Prepared according to *General procedures for halofluorination, Method B* (eluent gradient for column chromatography: *n*-hexane/acetone 14:1→10:1→8:1). White solid; yield 21%. R_f = 0.33 (*n*-hexane/acetone 3:1), mp. 120-137 °C.

^{1}H-NMR (500 MHz, $CDCl_3$, TMS): δ (ppm) = 2.02-2.12 (m, 1H, H-6), 2.42-2.57 (m, 2H, H-6 and H-3a), 2.66 (d, *J*=8.02 Hz, 1H, H-7a), 2.91 (d, *J*=4.05 Hz, 1H, H-7), 3.14 (d, *J*=8.27 Hz, 1H, H-4), 3.53-3.57 (m, 1H, H-8), 4.64 (s, 2H, benzylic CH_2), 4.70-4.85 (m, 1H, H-5), 7.28-7.38 (m, 5H, Ph).

^{19}F NMR (471 MHz, $CDCl_3$): δ (ppm) = -164.09.

^{13}C NMR (126 MHz, $CDCl_3$, TMS): δ (ppm) = 36.9 and 37.1 (^{2}J=21.51 Hz), 43.0, 43.3 and 43.4 (^{3}J=9.49 Hz), 45.3, 46.0, 46.1 and 46.1 (^{3}J=1.59 Hz), 49.8 and 50.0 (^{2}J=20.91 Hz), 92.8 and 94.3 (^{1}J=193.63 Hz), 128.4, 128.8, 128.9, 135.3, 175.2, 175.8.

HRMS calcd. for $C_{16}H_{16}BrFNO_2^+$ ($[M+H]^+$): 352.0343 (^{79}Br), 354.0322 (^{81}Br). Found: 352.0341 (^{79}Br), 354.0321 (^{81}Br).

(3a*S,4*R**,5*R**,6*S**,7*S**,7a*R**)-2-Benzyl-5,6-dibromohexahydro-1*H*-4,7-methanoisoindole-1,3(2*H*)-dione; 22**

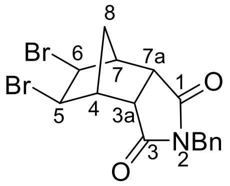

Prepared according to *General procedures for halofluorination, Method B* (yield: 15%, eluent gradient for column chromatography: *n*-hexane/acetone 14:1→10:1→8:1) or *Attempted*

*bromofluorination of N-benzyl imide **19** at reflux* (yield: 69%). Pale yellowish brown solid, R_f = 0.40 (*n*-hexane/acetone 3:1), mp. 199-204 °C.

^{1}H-NMR (500 MHz, $CDCl_3$, TMS): δ (ppm) = 1.67-1.73 (m, 1H, H-8), 2.46-2.52 (m, 1H, H-8), 3.05-3.11 (m, 2H, H-4 and H-7), 3.14-3.20 (m, 2H, H-3a and H-7a), 3.77-3.82 (m, 2H, H-5 and H-6), 4.63 (s, 2H, benzylic CH_2), 7.30-7.38 (m, 3H, CH-Ar), 7.38-7.44 (m, 2H, CH-Ar).

^{13}C NMR (126 MHz, $CDCl_3$, TMS): δ (ppm) = 37.7, 42.6, 47.4, 50.8, 51.2, 128.6, 128.9, 129.0, 135.6, 175.4.

HRMS calcd. for $C_{16}H_{16}Br_2NO_2^+$ ($[M+H]^+$): 411.9542 (2×^{79}Br), 413.9522 (^{79}Br + ^{81}Br), 415.9501 (2×^{81}Br). Found: 411.9537 (2×^{79}Br), 413.9517 (^{79}Br + ^{81}Br), 415.9495 (2×^{81}Br).

(3a*R,4*R**,5*R**,7*R**,7a*S**,8*R**)-2-Benzyl-5-fluoro-8-iodohexahydro-1*H*-4,7-methanoisoindole-1,3(2*H*)-dione; (*rac*)-20b**

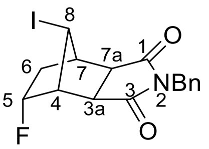

Prepared according to *General procedures for halofluorination, Method B* (eluent gradient for column chromatography: *n*-hexane/acetone 15:1→10:1, a subsequent crystallization was also necessary to obtain pure product). White solid; yield 11%. R_f = 0.57 (*n*-hexane/acetone 3:1), mp. 102-115 °C.

^{1}H-NMR (500 MHz, $CDCl_3$, TMS): δ (ppm) = 1.43-1.54 (m, 1H, H-6), 2.62-2.73 (m, 1H, H-6), 2.78-2.83 (m, 1H, H-7), 2.90 (d, *J*=8.02 Hz, 1H, H-7a), 3.08-3.13 (m, 1H, H-4), 3.25 (d, *J*=8.02 Hz, 1H, H-3a), 3.42-3.47 (m, 1H, H-8), 4.66 (s, 2H, benzylic CH_2), 5.38-5.55 (m, 1H, H-5), 7.27-7.38 (m, 5H, Ph).

^{19}F NMR (471 MHz, $CDCl_3$): δ (ppm) = -196.11.

^{13}C NMR (126 MHz, $CDCl_3$, TMS): δ (ppm) = 21.9 and 21.9 (^3J=3.73 Hz), 35.0 and 35.2 (^2J=22.70 Hz), 39.6 and 39.7 (^3J=13.09 Hz), 42.9, 46.0, 46.8, 50.3 and 50.4 (^2J=17.74 Hz), 92.4 and 93.9 (^1J=188.50 Hz), 128.2, 128.7, 128.8, 135.5, 176.5, 177.0.

HRMS calcd. for $C_{16}H_{16}FINO_2^+$ ($[M+H]^+$): 400.0204, found: 400.0202.

(3a*R,4*R**,5*R**,7*R**,7a*S**,8*R**)-2-Benzyl-8-chloro-5-fluorohexahydro-1*H*-4,7-methanoisoindole-1,3(2*H*)-dione;** (*rac*)-**20c**

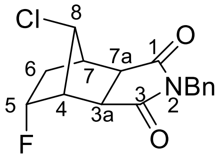

Prepared according to *General procedures for halofluorination, Method B* (eluent for column chromatography: *n*-hexane/acetone 14:1). White solid; yield 9%. R_f = 0.48 (*n*-hexane/acetone 3:1), mp. 136-146 °C.

^{1}H-NMR (500 MHz, $CDCl_3$, TMS): δ (ppm) = 1.44-1.56 (m, 1H, H-6), 2.59-2.71 (m, 1H, H-6), 2.77-2.82 (m, 1H, H-7), 2.93 (d, *J*=8.14 Hz, 1H, H-7a), 3.06-3.11 (m, 1H, H-4), 3.33 (dd, *J*=8.13 Hz, *J*=0.59 Hz, 1H, H-3a), 3.62-3.66 (m, 1H, H-8), 4.66 (s, 2H, benzylic CH_2), 5.39-5.56 (m, 1H, H-5), 7.27-7.35 (m, 3H, Ph), 7.35-7.40 (m, 2H, Ph).

^{19}F NMR (471 MHz, $CDCl_3$): δ (ppm) = -197.73.

^{13}C NMR (126 MHz, $CDCl_3$, TMS): δ (ppm) = 33.4 and 33.6 (^2J=22.23 Hz), 39.2 and 39.3 (^3J=13.07 Hz), 42.9, 44.9, 46.7, 48.9 and 49.0 (^2J=18.14 Hz), 60.4 and 60.5 (^3J=4.36 Hz), 90.8 and 92.3 (^1J=187.61 Hz), 128.3, 128.8, 128.9, 135.5, 176.4, 176.9.

MS (ESI) m/z = 308 [M+1]

HRMS calcd. for $C_{16}H_{16}ClFNO_2^+$ ($[M+H]^+$): 308.0848 (^{35}Cl), 310.0819 (^{37}Cl). Found: 308.0847 (^{35}Cl), 310.0817 (^{37}Cl).

(3a*R*,4*S*,7*R*,7a*S*)-2-Methyl-3a,4,7,7a-tetrahydro-1*H*-4,7-methanoisoindole-1,3(2*H*)-dione; 24

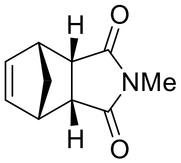

White solid; yield 50%. R_f = 0.37 (*n*-hexane/acetone 3:1), mp. 97-104 °C.

NMR data is identical to the one reported here: Chang, A. B.; Lin, T.-P.; Thompson, N. B.; Luo, S.-X.; Liberman-Martin, A. L.; Chen, H.-Y.; Lee, B.; Grubbs, R. H. *J. Am. Chem. Soc.* **2017**, *139*, 17683–17693.

MS (ESI) m/z = 178 [M+1]

HRMS calcd. for $C_{10}H_{12}NO_2^+$ ($[M+H]^+$): 178.0863, found: 178.0864.

(3a*R,4*R**,5*R**,7*R**,7a*S**,8*R**)-8-Bromo-5-fluoro-2-methylhexahydro-1*H*-4,7-methanoisoindole-1,3(2*H*)-dione; (*rac*)-25a**

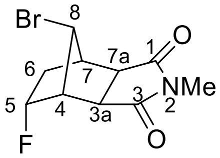

Prepared according to *General procedures for halofluorination, Method B* (eluent gradient for column chromatography: *n*-hexane/acetone 14:1→12:1). White solid; yield 19%. R_f = 0.42 (*n*-hexane/acetone 3:1), mp. 61-63 °C.

^{1}H-NMR (500 MHz, $CDCl_3$, TMS): δ (ppm) = 1.46-1.60 (m, 1H, H-6), 2.63-2.78 (m, 1H, H-6), 2.80-2.88 (m, 1H, H-7), 2.96 (d, *J*=8.01 Hz, 1H, H-7a), 3.03 (s, 3H, CH_3), 3.10-3.18 (m, 1H, H-4), 3.34 (d, *J*=8.02 Hz, 1H, H-3a), 3.72 (s, 1H, H-8), 5.35-5.66 (m, 1H, H-5).

^{19}F NMR (471 MHz, $CDCl_3$): δ (ppm) = -197.02.

^{13}C NMR (126 MHz, $CDCl_3$, TMS): δ (ppm) = 25.2, 34.0 and 34.2 (^2J=22.55 Hz), 39.6 and 39.7 (^3J=13.54 Hz), 45.3, 46.7, 49.1 and 49.2 (^2J=18.19 Hz), 49.7 and 49.7 (^3J=4.16 Hz), 91.3 and 92.8 (^1J=188.17 Hz), 176.8, 177.3.

HRMS calcd. for $C_{10}H_{12}BrFNO_2^+$ ([M+H]$^+$): 276.0030 (^{79}Br), 278.0009 (^{81}Br). Found: 276.0030 (^{79}Br), 278.0010 (^{81}Br).

(3a*S,4*R**,5*R**,6*S**,7*S**,7a*R**)-5,6-Dibromo-2-methylhexahydro-1*H*-4,7-methanoisoindole-1,3(2*H*)-dione; 26**

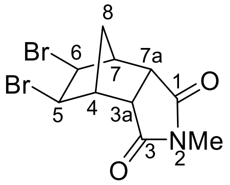

Prepared according to *General procedures for halofluorination, Method B* (eluent gradient for column chromatography: *n*-hexane/acetone 14:1→12:1). Pale yellowish brown solid; yield 33%. R_f = 0.31 (*n*-hexane/acetone 3:1), mp. 251-252 °C.

^{1}H-NMR (500 MHz, $CDCl_3$, TMS): δ (ppm) = 1.72-1.79 (m, 1H, H-8), 2.53-2.60 (m, 1H, H-8), 2.99 (s, 3H, N-CH_3), 3.09-3.16 (m, 2H, H-4 and H-7), 3.17-3.25 (m, 2H, H-3a and H-7a), 4.08-4.13 (m, 2H, H-5 and H-6).

^{13}C NMR (126 MHz, $CDCl_3$, TMS): δ (ppm) = 24.8, 37.7, 47.8, 50.4, 51.6, 175.7.

HRMS calcd. for $C_{10}H_{12}Br_2NO_2^+$ ([M+H]$^+$): 335.9230 (2×^{79}Br), 337.9209 (^{79}Br + ^{81}Br), 339.9189 (2×^{81}Br). Found: 335.9225 (2×^{79}Br), 337.9205 (^{79}Br + ^{81}Br), 339.9184 (2×^{81}Br).

(3a*R,4*R**,5*R**,7*R**,7a*S**,8*R**)-5-Fluoro-8-iodo-2-methylhexahydro-1*H*-4,7-methanoisoindole-1,3(2*H*)-dione;** (*rac*)-**25b**

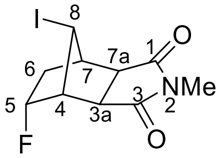

Prepared according to *General procedures for halofluorination, Method B* (eluent for column chromatography: *n*-hexane/acetone 14:1). White solid; yield 11%. R_f = 0.48 (*n*-hexane/acetone 3:1), mp. 108-110 °C.

^{1}H-NMR (500 MHz, $CDCl_3$, TMS): δ (ppm) = 1.52 (ddt, *J*=27.13 Hz, *J*=14.53 Hz, *J*=2.65 Hz, 1H, H-6), 2.64-2.77 (m, 1H, H-6), 2.79-2.85 (m, 1H, H-7), 2.93 (d, *J*=8.00 Hz, 1H, H-7a), 3.03 (s, 3H, CH_3), 3.09-3.16 (m, 1H, H-4), 3.27 (d, *J*=8.00 Hz, 1H, H-3a), 3.52-3.60 (m, 1H, H-8), 5.40-5.59 (m, 1H, H-5).

^{19}F NMR (471 MHz, $CDCl_3$): δ (ppm) = -196.06.

^{13}C NMR (126 MHz, $CDCl_3$, TMS): δ (ppm) = 22.4 and 22.4 (^3J=3.73 Hz), 25.3, 35.0 and 35.2 (^2J=22.27 Hz), 39.7 and 39.8 (^3J=13.51 Hz), 46.1, 46.6, 50.1 and 50.2 (^2J=17.84 Hz), 92.5 and 94.0 (^1J=188.53 Hz), 177.0, 177.5.

HRMS calcd. for $C_{10}H_{12}FINO_2^+$ ($[M+H]^+$): 323.9891, found: 323.9890.

Dimethyl (1*S,2*R**,4*R**,5*R**)-4-fluorobicyclo[3.1.0]hexane-1,2-dicarboxylate;** (*rac*)-**27**

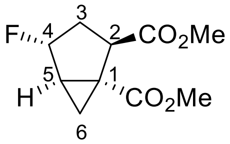

Colorless oil; yields: 52% from (*rac*)-**2a** (eluent gradient for column chromatography: *n*-hexane/acetone 18:1→15:1), 53% from (*rac*)-**2b** (eluent gradient for column chromatography: *n*-hexane/acetone 18:1→17:1), 54% from (*rac*)-**5a** (eluent gradient for column chromatography: *n*-hexane/EtOAc 8:1→6:1), 31% from (*rac*)-**6b** (eluent gradient for column chromatography: *n*-hexane/EtOAc 7:1→5:1). R_f = 0.36 (*n*-hexane/EtOAc 4:1).

^{1}H-NMR (500 MHz, D_6-DMSO, TMS): δ (ppm) = 1.08-1.17 (m, 1H, H-6), 1.46-1.56 (m, 1H, H-6), 1.69-1.87 (m, 1H, H-3), 2.07-2.25 (m, 2H, H-3 and H-5), 3.55-3.67 (m, 7H, 2×OCH_3 and H-2), 5.02-5.20 (m, 1H, H-4).

^{19}F NMR (471 MHz, $CDCl_3$): δ (ppm) = -169.24.

^{13}C NMR (126 MHz, D_6-DMSO, TMS): δ (ppm) = 14.4 and 14.5 (^3J=9.63 Hz), 32.4, 33.6 and 33.7 (^2J=19.66 Hz), 33.8 and 34.0 (^2J=27.88 Hz), 42.6, 52.2, 52.4, 94.4 and 95.7 (^1J=170.35 Hz), 172.2, 173.3.

MS (ESI) m/z = 217 [M+1]

HRMS calcd. for $C_{10}H_{14}FO_4^+$ ($[M+H]^+$): 217.0871, found: 217.0868.

(3a*S,5*R**,5a*R**,6a*S**)-2-Benzyl-5-fluorotetrahydrocyclopropa[1,5]cyclopenta[1,2-c]pyrrole-1,3(2*H*,3a*H*)-dione;** (±)-**28**

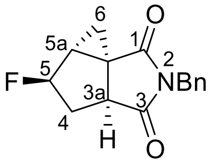

White solid; yields: 71% from (*rac*)-**8a** (eluent for column chromatography: *n*-hexane/EtOAc 5:3), 73% from (*rac*)-**8b** (eluent for column chromatography: *n*-hexane/acetone 6:1), 86% from (*rac*)-**11a** (eluent gradient for column chromatography: *n*-hexane/EtOAc 3:1→1:1), 74% from (*rac*)-**11b** (eluent for column chromatography: *n*-hexane/EtOAc 3:2). R_f = 0.40 (*n*-hexane/EtOAc 3:2), mp. 56-67 °C.

^1H-NMR (500 MHz, D_6-DMSO, TMS): δ (ppm) = 1.27-1.34 (m, 1H, H-6), 1.82-1.90 (m, 1H, H-6), 1.99-2.22 (m, 2H, H-4 and H-5a), 2.30-2.43 (m, 1H, H-4), 3.36-3.41 (m, 1H, H-3a), 4.56-4.65 (m, 2H, benzylic CH_2), 5.12-5.27 (m, 1H, H-5), 7.17-7.22 (m, 2H, Ph), 7.22-7.28 (m, 1H, Ph), 7.28-7.34 (m, 2H, Ph).

^{19}F NMR (471 MHz, D_6-DMSO): δ (ppm) = -160.94.

^{13}C NMR (126 MHz, $CDCl_3$, TMS): δ (ppm) = 13.3 and 13.4 (^3J=10.02 Hz), 33.8 and 34.0 (^2J=21.20 Hz), 35.3 and 35.6 (^2J=31.03 Hz), 35.6, 42.3, 46.1, 95.0 and 96.4 (^1J=172.58 Hz), 127.7, 128.1, 128.6, 135.9, 175.3, 176.9.

MS (ESI) m/z = 260 [M+1]

HRMS calcd. for $C_{15}H_{15}FNO_2^+$ ($[M+H]^+$): 260.1081, found: 260.1083.

(1*S,2*S**,4*S**,5*S**)-Dimethyl 4-fluoro-5-(phenylselanyl)cyclohexane-1,2-dicarboxylate; (*rac*)-30**

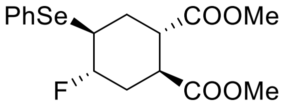

Prepared according to *General procedure for fluoroselenation* (yield: 37%) or *Alternative procedure for fluoroselenation of diester* **(±)-*1*** (yield: 35%), in both cases the eluent for column chromatography was *n*-hexane/acetone 18:1. Yellow oil, R_f = 0.45 (*n*-hexane/EtOAc 4:1).

^{1}H-NMR (500 MHz, $CDCl_3$, TMS): δ (ppm) = 2.06-2.37 (m, 4H, 2×CH_2), 2.97-3.10 (m, 2H, H-1 and H-2), 3.61-3.68 (m, 1H, H-5), 3.70 (s, 3H, OCH_3), 3.71 (s, 3H, OCH_3), 4.79-4.95 (m, 1H, H-4), 7.28-7.37 (m, 3H CH-Ar), 7.54-7.60 (m, 2H, CH-Ar).

^{19}F NMR (471 MHz, $CDCl_3$): δ (ppm) = -170.31.

^{13}C NMR (126 MHz, $CDCl_3$, TMS): δ (ppm) = 28.9, 29.0 and 29.2 (^{2}J=21.43 Hz), 39.2, 40.4, 42.2 and 42.3 (^{2}J=19.31 Hz), 52.1, 88.5 and 90.0 (^{1}J=178.14 Hz), 128.2, 128.3, 129.5, 134.5, 174.3, 174.4

HRMS calcd. for $C_{16}H_{19}FNaO_4Se^+$ ($[M+Na]^+$): 397.0325 (^{80}Se), 395.0333 (^{78}Se). Found: 397.0322 (^{80}Se), 395.0332 (^{78}Se).

(1*R,2*S**)-Dimethyl 4-fluoro-5-(phenylselanyl)cyclohexane-1,2-dicarboxylate; (*rac*)-31**

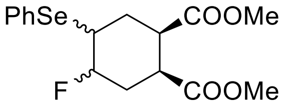

Prepared according to *General procedure for fluoroselenation* (yield: 38%) or *Alternative procedure for fluoroselenation of diester **4*** (yield: 32%), in both cases the eluent gradient for column chromatography was *n*-hexane/acetone 19:1→15:1). Pale brown oil (9:5 mixture of 2 diastereomers).

(3*R,3a*R**,5*S**,6*S**,6a*S**,7*R**)-Methyl 2-oxo-6-(phenylselanyl)hexahydro-2*H*-3,5-methanocyclopenta[*b*]furan-7-carboxylate; (*rac*)-32**

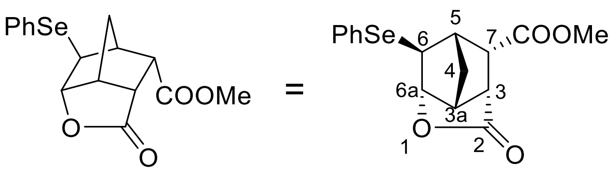

Prepared according to *General procedure for fluoroselenation* (yield: 76%, eluent gradient for column chromatography was *n*-hexane/acetone 7:1→6:1) or *Alternative preparation of lacton* **(±)-*32*** (yield: 93%). Yellowish solid, R_f = 0.29 (*n*-hexane/EtOAc 2:1), mp. 73-74 °C.

^{1}H-NMR (500 MHz, $CDCl_3$, TMS): δ (ppm) = 1.73 (d, *J*=11.54 Hz, 1H, CH_2), 2.30 (d, *J*=11.56 Hz, 1H, CH_2), 2.72-2.77 (m, 1H, H-5), 2.84 (dd, *J*=10.70 Hz, *J*=4.53 Hz, 1H, H-3), 3.14 (dd, *J*=10.76 Hz, *J*=2.98 Hz, 1H, H-7), 3.32-3.38 (m, 1H, H-3a), 3.72 (s, 3H, OCH_3), 4.06-4.10 (m, 1H, H-6), 4.79-4.84 (m, 1H, H-6a), 7.27-7.34 (m, 3H, CH-Ar), 7.47-7.53 (m, 2H, CH-Ar).
^{13}C NMR (126 MHz, $CDCl_3$, TMS): δ (ppm) = 36.60, 41.22, 44.01, 45.23, 48.18, 49.26, 52.16, 86.14, 127.49, 128.74, 129.45, 132.59, 170.68, 176.78.
MS (ESI) m/z = 351 {[^{78}Se]M+1}, 353 {[^{80}Se]M+1}.
HRMS calcd. for $C_{16}H_{17}O_4Se^+$ ([M+H]$^+$): 351.0294 (^{78}Se), 353.0287 (^{80}Se). Found: 351.0298 (^{78}Se), 353.0286 (^{80}Se).

(1*S,2*S**,5*S**)-Dimethyl 5-fluorocyclohex-3-ene-1,2-dicarboxylate;** (*rac*)-**33**

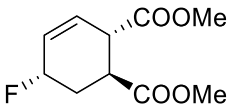

Pale yellow oil; yield 82%. R_f = 0.35 (*n*-hexane/EtOAc 6:1).
^{1}H-NMR (500 MHz, $CDCl_3$, TMS): δ (ppm) = 1.86 (dddd, *J*=36.08 Hz, *J*=14.52 Hz, *J*=12.85 Hz, *J*=3.77 Hz, 1H, H-6), 2.36-2.46 (m, 1H, H-6), 3.17 (ddd, *J*=12.83 Hz, *J*=10.17 Hz, *J*=3.37 Hz, 1H, H-1), 3.47-3.56 (m, 1H, H-2), 3.74 (s, 3H, OCH_3), 3.74 (s, 3H, OCH_3), 4.90-5.05 (m, 1H, H-5), 5.93-6.03 (m, 1H, H-4), 6.14 (ddd, *J*=10.07 Hz, *J*=4.03 Hz, *J*=2.53 Hz, 1H, H-3).
^{19}F NMR (471 MHz, $CDCl_3$): δ (ppm) = -165.61.
^{13}C NMR (126 MHz, $CDCl_3$, TMS): δ (ppm) = 30.9 and 31.1 (^{2}J=22.10 Hz), 36.8, 44.1 and 44.1 (^{3}J=3.51 Hz), 52.2, 52.5, 81.7 and 83.0 (^{1}J=165.92 Hz), 125.3 and 125.4 (^{2}J=16.64 Hz), 130.6 and 130.7 (^{3}J=9.52 Hz), 171.9, 174.6.
HRMS calcd. for $C_{10}H_{13}FNaO_4^+$ ([M+Na]$^+$): 239.0690, found: 239.0688.

(±)-Dimethyl cyclohexa-2,4-diene-1,2-dicarboxylate; (*rac*)-**34**

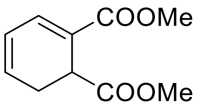

Colorless oil; yields: 40% from (*rac*)-**30**, 37% from (*rac*)-**31**. R_f = 0.45 (*n*-hexane/EtOAc 4:1)
^{1}H-NMR (500 MHz, $CDCl_3$, TMS): δ (ppm) = 2.47-2.58 (m, 1H, H-6), 2.86-2.97 (m, 1H, H-6), 3.64-3.70 (m, 4H, OCH_3 and H-1), 3.78 (s, 3H, OCH_3), 6.08-6.15 (m, 2H, H-4 and H-5), 7.12-7.16 (m, 1H, H-3).
^{13}C NMR (126 MHz, $CDCl_3$, TMS): δ (ppm) = 26.43, 36.64, 51.89, 52.27, 123.72, 125.07, 132.12, 134.10, 167.10, 173.53.

HRMS calcd. for $C_{10}H_{13}O_4^+$ ($[M+H]^+$): 197.0808, found: 197.0802.

(1*S,2*R**,5*S**)-Dimethyl 5-fluorocyclohex-3-ene-1,2-dicarboxylate;** (*rac*)**-35**

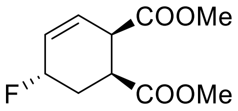

Pale yellow oil; yield 46%. R_f = 0.37 (*n*-hexane/EtOAc 6:1).

^{1}H-NMR (500 MHz, D_6-DMSO, TMS): δ (ppm) = 2.10-2.32 (m, 2H, H-6), 2.93-3.06 (m, 1H, H-1), 3.60 (s, 3H, OCH_3), 3.61 (s, 3H, OCH_3), 3.64-3.72 (m, 1H, H-2), 5.01-5.17 (m, 1H, H-5), 5.97-6.04 (m, 1H, H-4), 6.09-6.16 (m, 1H, H-3).

^{19}F NMR (471 MHz, $CDCl_3$): δ (ppm) = -167.25.

^{13}C NMR (126 MHz, $CDCl_3$, TMS): δ (ppm) = 28.0 and 28.1 (^2J=21.62 Hz), 36.5, 42.1 and 42.1 (^4J=3.00 Hz), 52.0, 52.1, 83.0 and 84.3 (^1J=163.57 Hz), 126.6 and 126.7 (^2J=16.26 Hz), 130.0 and 130.1 (^3J=9.90 Hz), 171.0 and 171.1 (^4J=6.56 Hz), 173.1.

MS (ESI) m/z = 217 [M+1]

HRMS calcd. for $C_{10}H_{13}FNaO_4^+$ ($[M+Na]^+$): 239.0690, found: 239.0687.

Dimethyl (1*R,2*S**,4*R**)-4-fluorocyclohexane-1,2-dicarboxylate;** (*rac*)**-36**

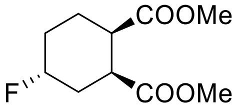

Colorless oil; yield 88%. R_f = 0.48 (*n*-hexane/EtOAc 4:1).

^{1}H-NMR (500 MHz, $CDCl_3$, TMS): δ (ppm) = 1.58-1.75 (m, 1H, H-5), 1.77-1.88 (m, 1H, H-5), 1.95-2.07 (m, 2H, H-6), 2.11-2.27 (m, 2H, H-3), 2.96-3.02 (m, 1H, H-2), 3.07-3.13 (m, 1H, H-1), 3.68 (s, 3H, OCH_3), 3.69 (s, 3H, OCH_3), 4.80-4.95 (m, 1H, H-4).

^{19}F NMR (471 MHz, $CDCl_3$): δ (ppm) = -183.34.

^{13}C NMR (126 MHz, $CDCl_3$, TMS): δ (ppm) = 22.0 and 22.0 (^3J=4.11 Hz). 27.6 and 27.7 (^2J=20.85 Hz), 30.0 and 30.1 (^2J=20.61 Hz), 38.6 and 38.7 (^3J=3.31 Hz), 41.1, 51.7, 51.8, 87.6 and 89.0 (^1J=168.86 Hz), 173.5, 173.7.

MS (ESI) m/z = 219 [M+1]

HRMS calcd. for $C_{10}H_{16}FO_4^+$ ($[M+H]^+$): 219.1027, found: 219.1023.

---------------X-ray structure determination---------------

The crystals of (*rac*)-**11b**, (*rac*)-**15a**, and (*rac*)-**15b**, respectively, were immersed in cryo-oil, mounted in a loop, and measured in the temperature range 120 K–170 K. The X-ray diffraction data were collected on a Rigaku Oxford Diffraction Supernova ((*rac*)-**11b** and (*rac*)-**15b**) or on a Bruker Kappa Apex II ((*rac*)-**15a**) diffractometer using Cu Kα ((*rac*)-**11b** and (*rac*)-**15b**]) or Mo Kα ((*rac*)-**15a**) radiation. The CrysAlisPro[RS3] ((*rac*)-**11b**, (*rac*)-**15b**)) or the Denzo-Scalepac[RS4] ((*rac*)-**15a**) software packages were used for cell refinements and data reductions. A multiscan ((*rac*)-**11a**), numerical ((*rac*)-**15a**) or spherical ((*rac*)-**15b**) absorption correction (SADABS[RS5] ((*rac*)-**11b** and (*rac*)-**15a**) or CrysAlisPro[RS3] ((*rac*)-**15b**))) was applied to all data. The structures were solved by intrinsic phasing method using the SHELXT[*R54*] software. Structural refinements were carried out using the SHELX[RS6] software. The higher residual electron density near the atom I1 in (*rac*)-**11b** is most likely due to a slight disorder. No disorder model was used for the final structure refinement process. Hydrogen atoms were positioned geometrically and constrained to ride on their parent atoms, with C–H = 0.985–1.00 Å and U_{iso} = 1.2–1.5 U_{eq} (parent atom). The crystallographic details are summarized in Table XS1.

Table XS1. Crystal Data.

	(*rac*)-**11a**	(*rac*)-**15a**	(*rac*)-**15b**
CCDC	2011186	2011187	2011188
empirical formula	$C_{15}H_{15}FINO_2$	$C_{11}H_{15}BrFNO_3$	$C_{11}H_{15}FINO_3$
fw	387.18	308.15	355.14
temp (K)	120(2)	170(2)	120(2)
λ (Å)	1.54184	0.71073	1.54184
cryst syst	Monoclinic	Monoclinic	Monoclinic
space group	$P2_1/c$	$P2_1/n$	$P2_1/n$
a (Å)	11.8964(3)	9.1284(3)	9.15590(10)
b (Å)	11.5192(2)	7.8888(2)	7.95180(10)
c (Å)	10.9595(3)	17.2125(6)	17.6478(2)
β (deg)	109.641(2)	101.0830(10)	102.1100(10)
V (Å^3)	1414.47(6)	1216.39(7)	1256.27(3)
Z	4	4	4
ρ_{calc} (mg/m^3)	1.818	1.683	1.878
μ(Mo Kα) (mm^{-1})	17.911	3.389	20.149
no. reflns.	15693	18426	29791
unique reflns.	2552	3544	2649
GOOF (F^2)	1.046	1.086	1.107
R_{int}	0.0299	0.0328	0.0581
$R1^a$ ($I \geq 2\sigma$)	0.0387	0.0305	0.0253
$wR2^b$ ($I \geq 2\sigma$)	0.0882	0.0624	0.0688

$^a R1 = \Sigma||F_o| - |F_c||/\Sigma|F_o|$. $^b wR2 = [\Sigma[w(F_o^2 - F_c^2)^2]/ \Sigma[w(F_o^2)^2]]^{1/2}$.

References

RS1. Camm, K. D.; Castro, N. M.; Liu, Y.; Czechura, P.; Snelgrove, J. L.; Fogg, D. E. *J. Am. Chem. Soc.* **2007**, *129*, 4168–4169.

RS2. Windmon, N.; Dragojlovic, V *Beilstein J. Org. Chem.* **2008**, *4(29):* doi:10.3762/bjoc.4.29.

RS3. Otwinowski, Z.; Minor, W. Processing of X-ray Diffraction Data Collected in Oscillation Mode, Academic Press, New York, pp. 307-326, 1997. In *Methods in Enzymology, Volume 276, Macromolecular Crystallography, Part A*, Carter, C. W., Sweet, J., Eds.; Academic Press: New York, USA, 1997; pp 307-326.

RS4. Rikagu Oxford Diffraction, *CrysAlisPro*, Agilent Technologies inc., 2013, Yarnton, Oxfordshire, England.

RS5. Sheldrick, G. M. *SADABS - Bruker Nonius scaling and absorption correction* -, Bruker AXS, Inc.: Madison, Wisconsin, USA, 2012.

RS6. Sheldrick, G. M. *Acta Cryst.* **2015**, *C71*, 3-8.

---------------Copies of ^{1}H and ^{13}C NMR spectra---------------

(1*S**,2*S**,4*S**,5*S**)-Dimethyl 4-bromo-5-fluorocyclohexane-1,2-dicarboxylate; (*rac*)-2a

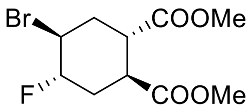

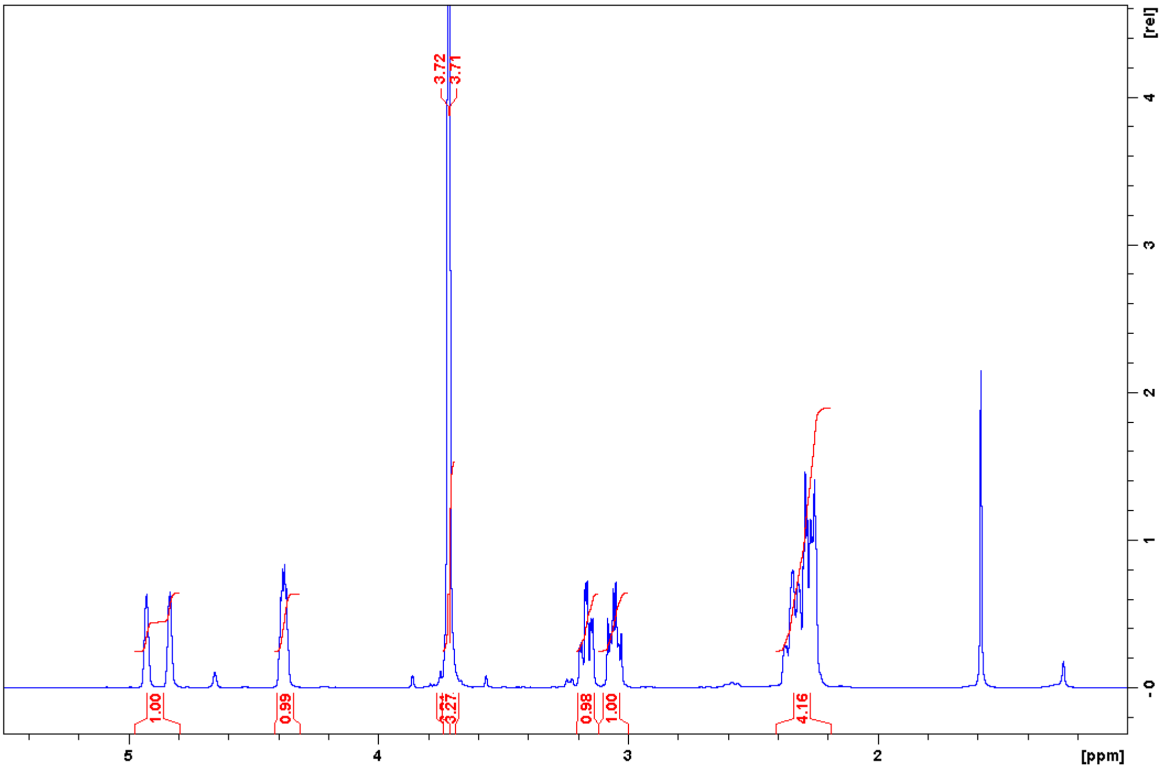

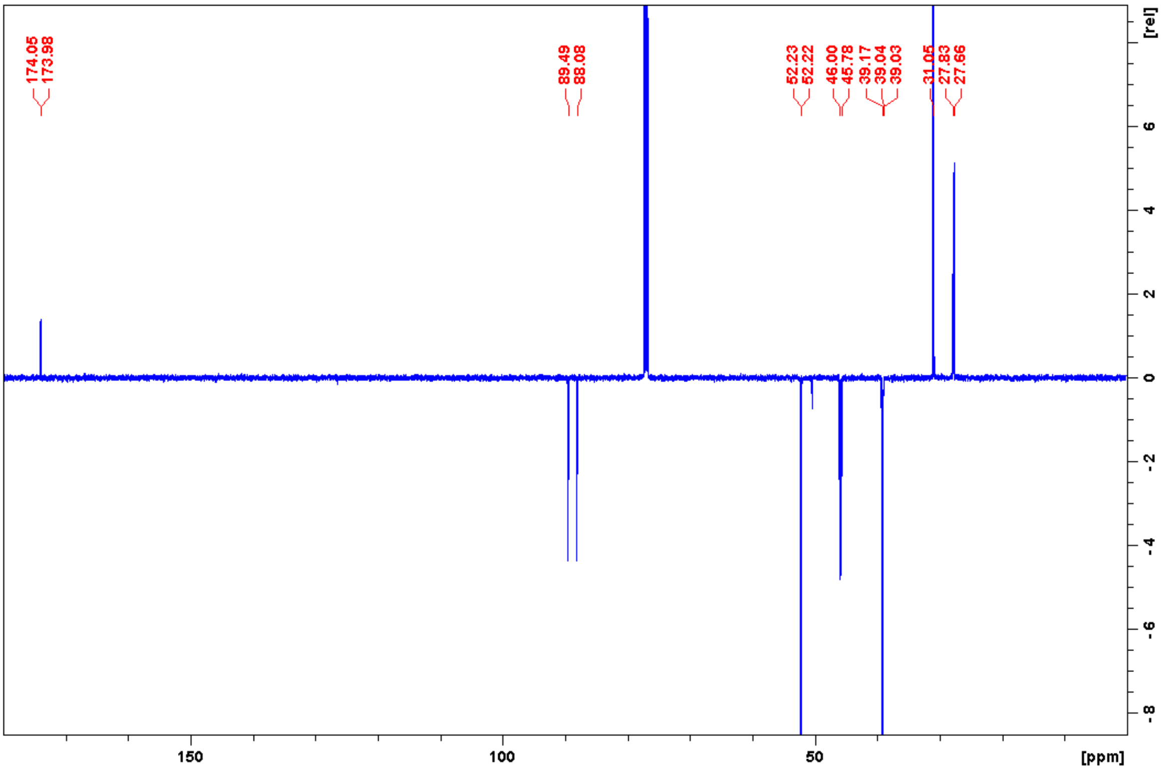

(1*S,2*S**,4*S**,5*S**)-Dimethyl 4-fluoro-5-iodocyclohexane-1,2-dicarboxylate; (*rac*)-2b**

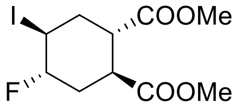

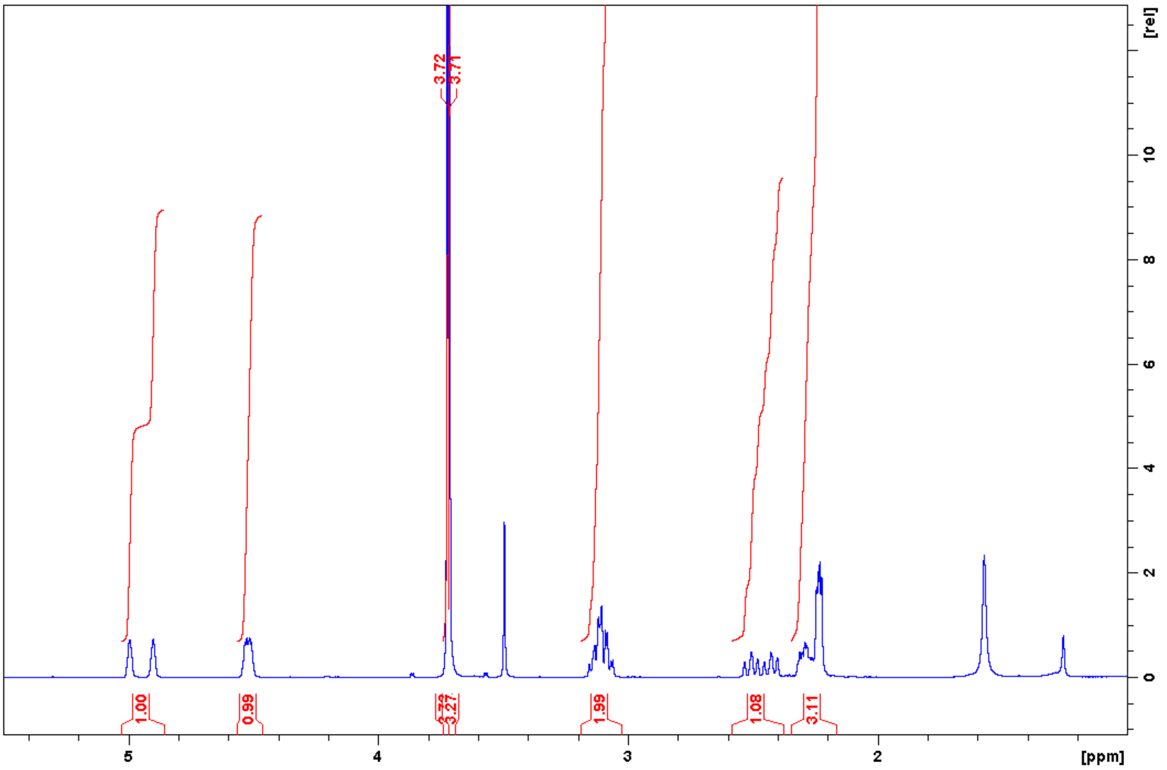

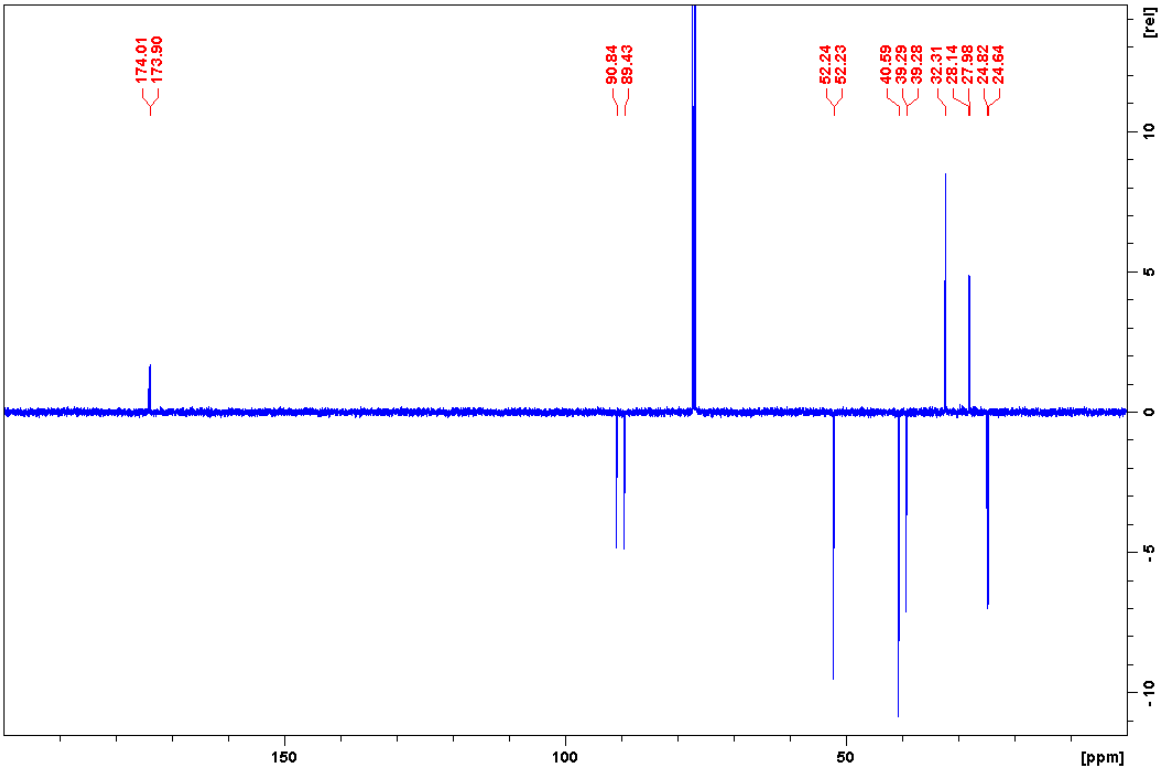

(1*R,2*S**,4*R**,5*R**)-Dimethyl 4-bromo-5-fluorocyclohexane-1,2-dicarboxylate; (*rac*)-5a**

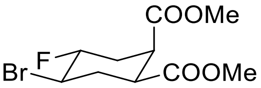

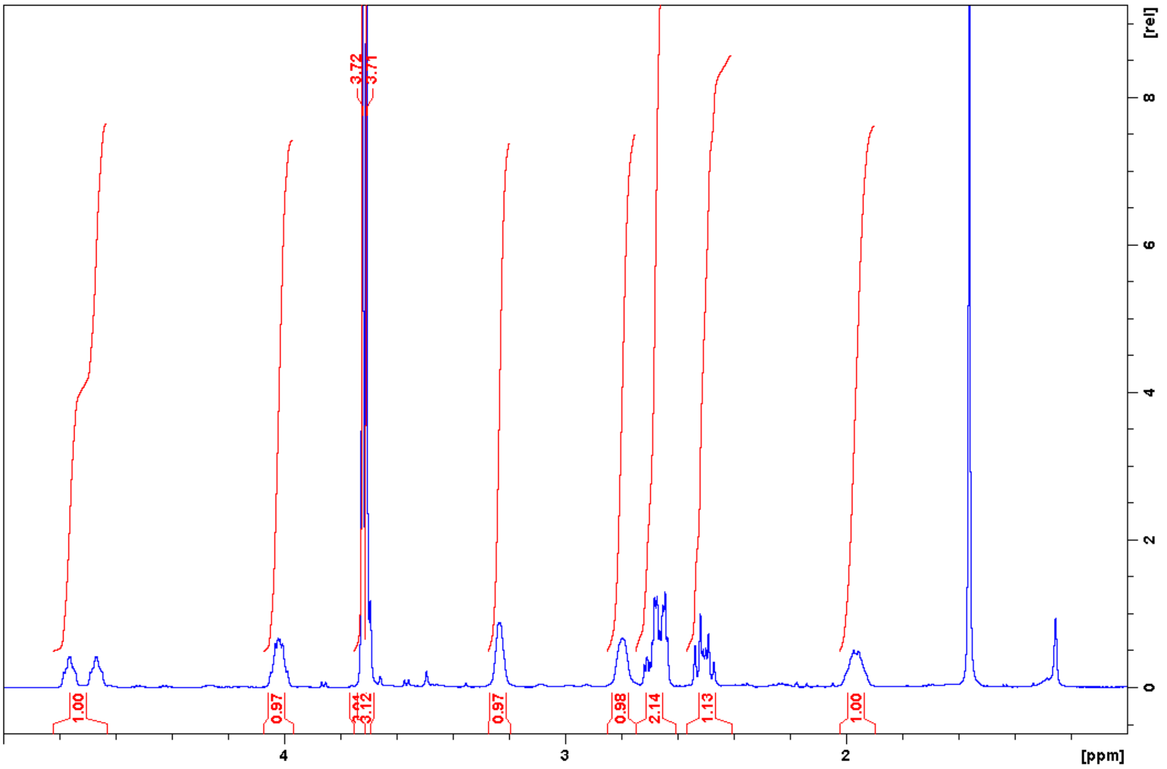

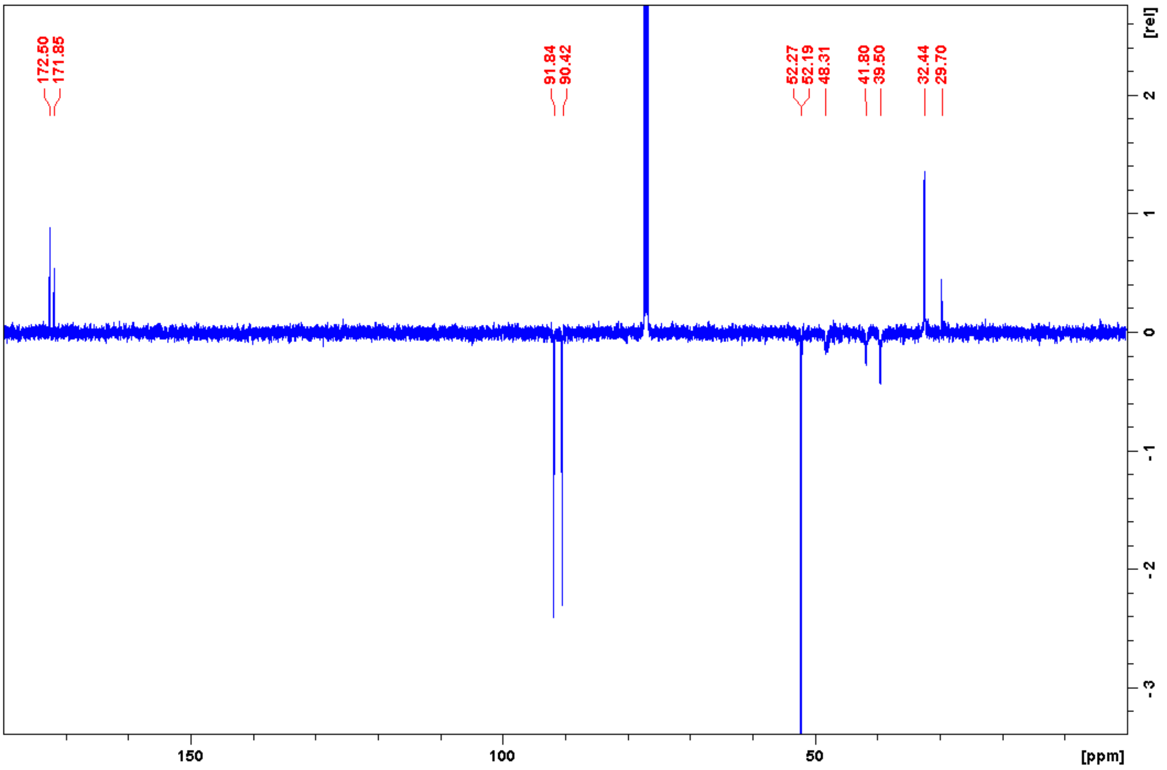

(1*S,2*R**,4*R**,5*R**)-Dimethyl 4-fluoro-5-iodocyclohexane-1,2-dicarboxylate; (*rac*)-6b**

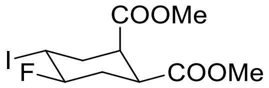

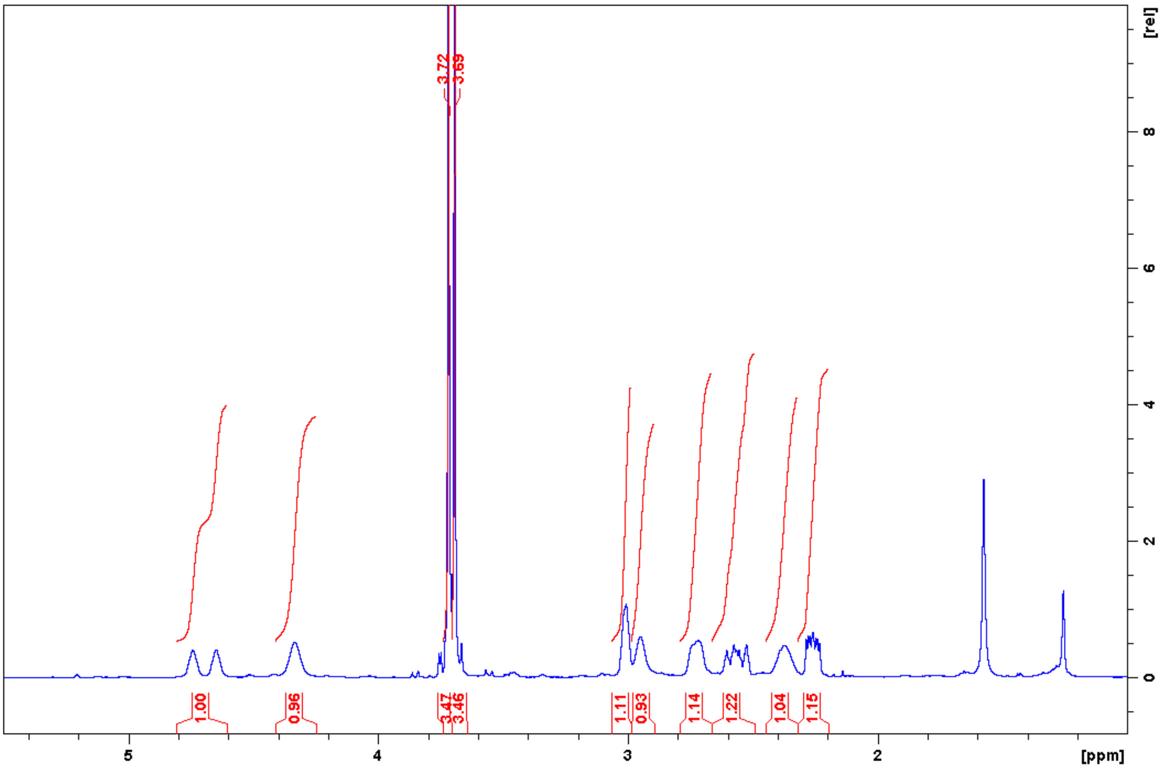

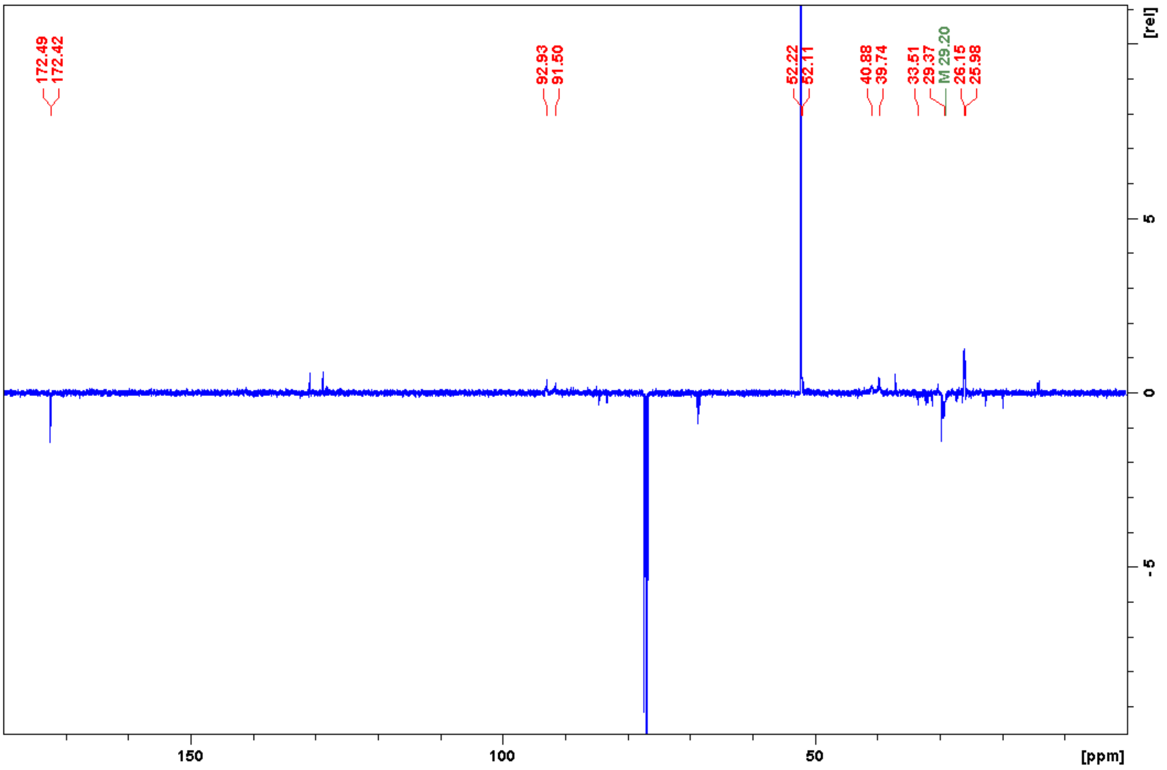

(1*R,2*S**,4*R**,5*R**)-Dimethyl 4-fluoro-5-iodocyclohexane-1,2-dicarboxylate; (*rac*)-5b**

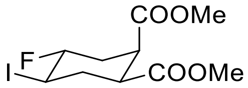

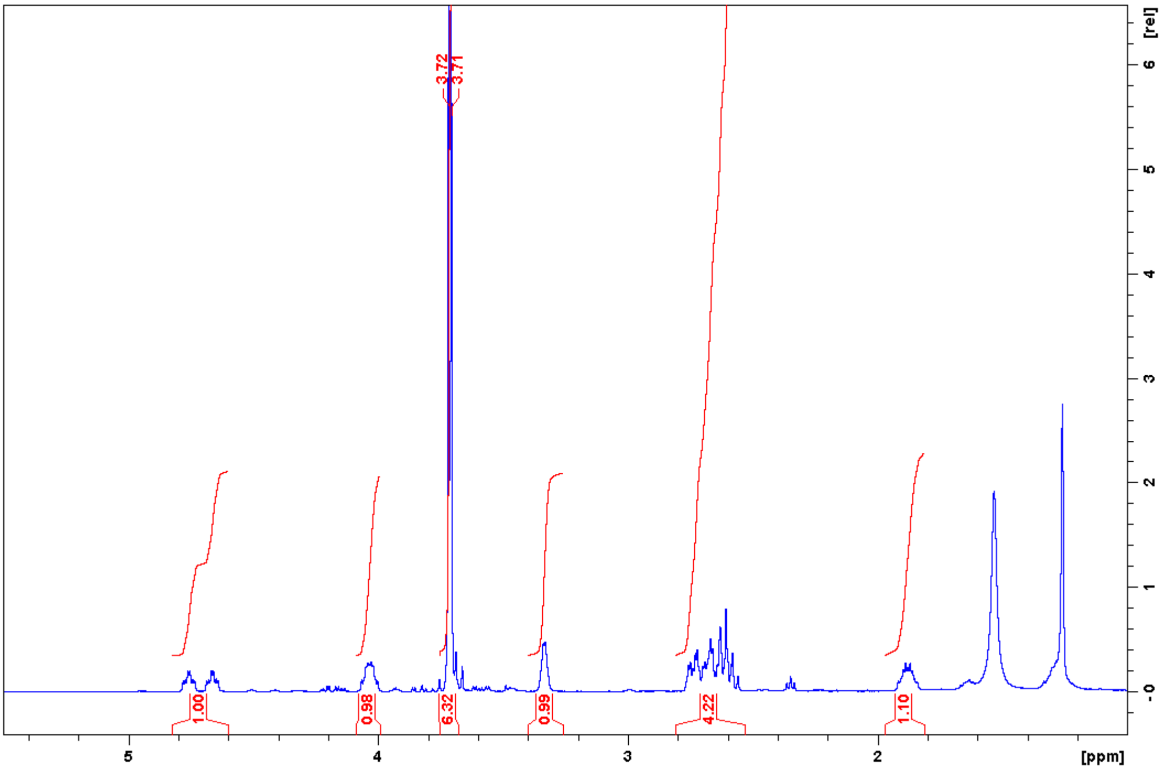

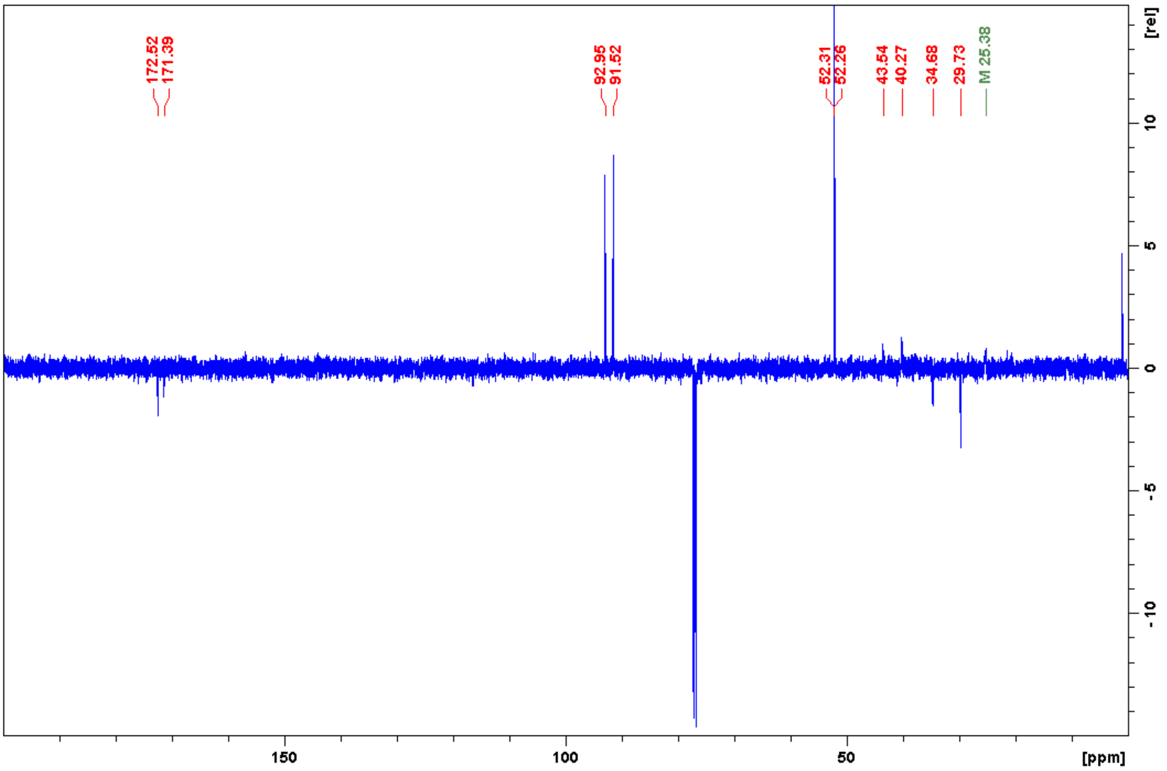

(3a*R,7a*S**)-2-Benzyl-5-bromo-6-fluorohexahydro-1*H*-isoindole-1,3(2*H*)-dione; (*rac*)-8a**

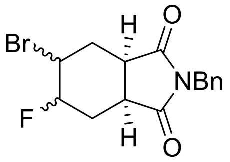

(7:5 mixture of 2 diastereomers)

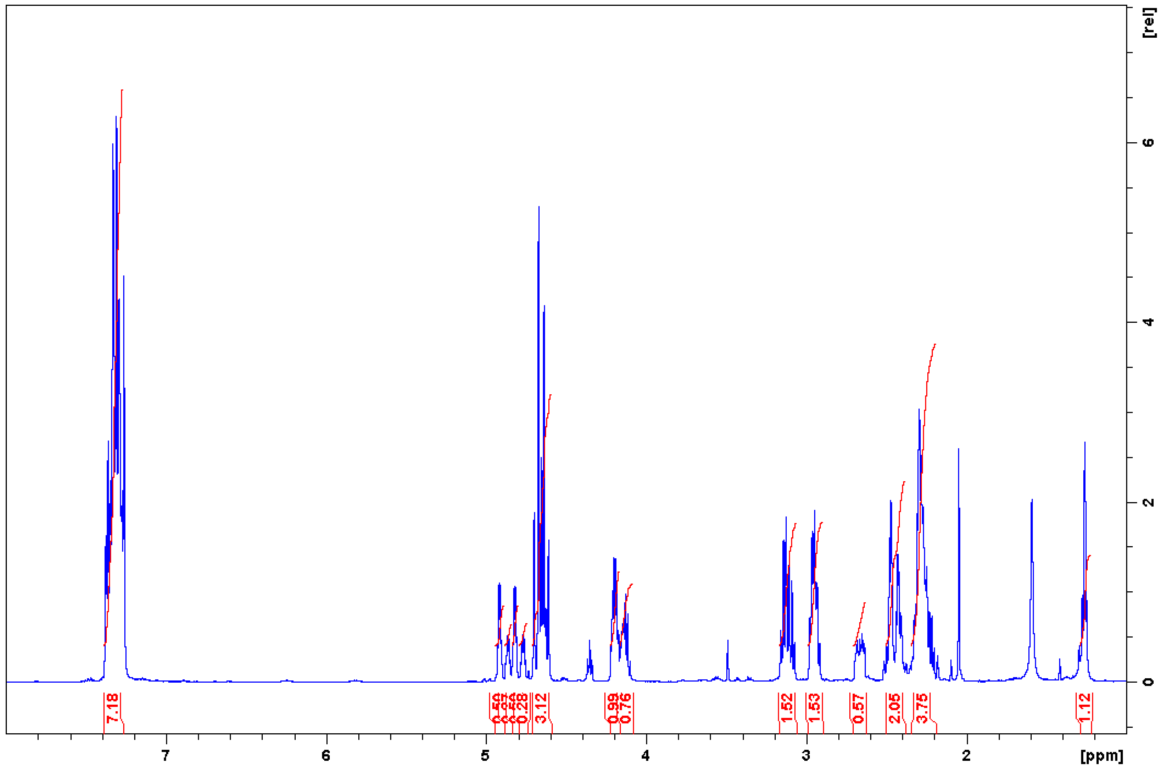

(3aS*,7aR*)-2-Benzyl-5-fluoro-6-iodohexahydro-1*H*-isoindole-1,3(2*H*)-dione; (*rac*)-8b

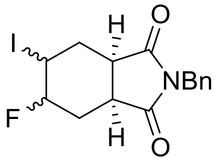

(2:1 mixture of 2 diastereomers)

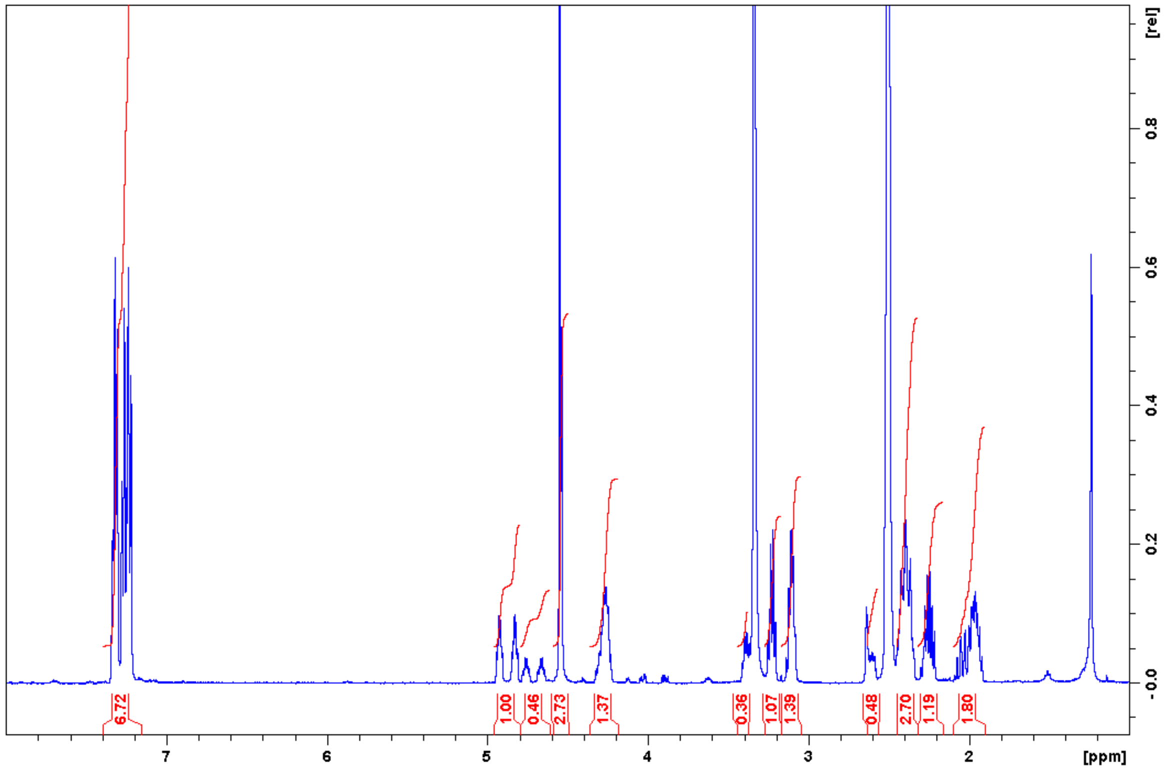

(3a*S,7a*S**)-2-Benzyl-3a,4,7,7a-tetrahydro-1*H*-isoindole-1,3(2*H*)-dione; (*rac*)-10**

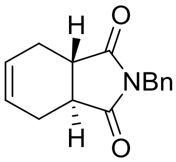

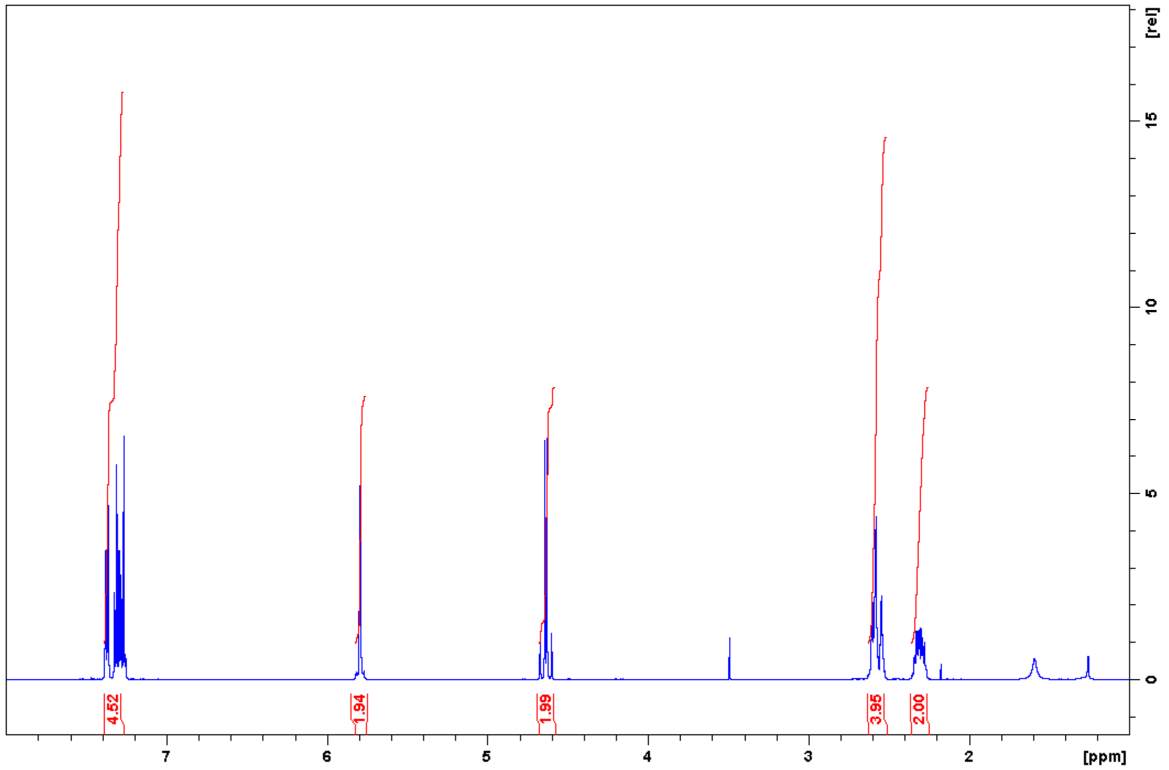

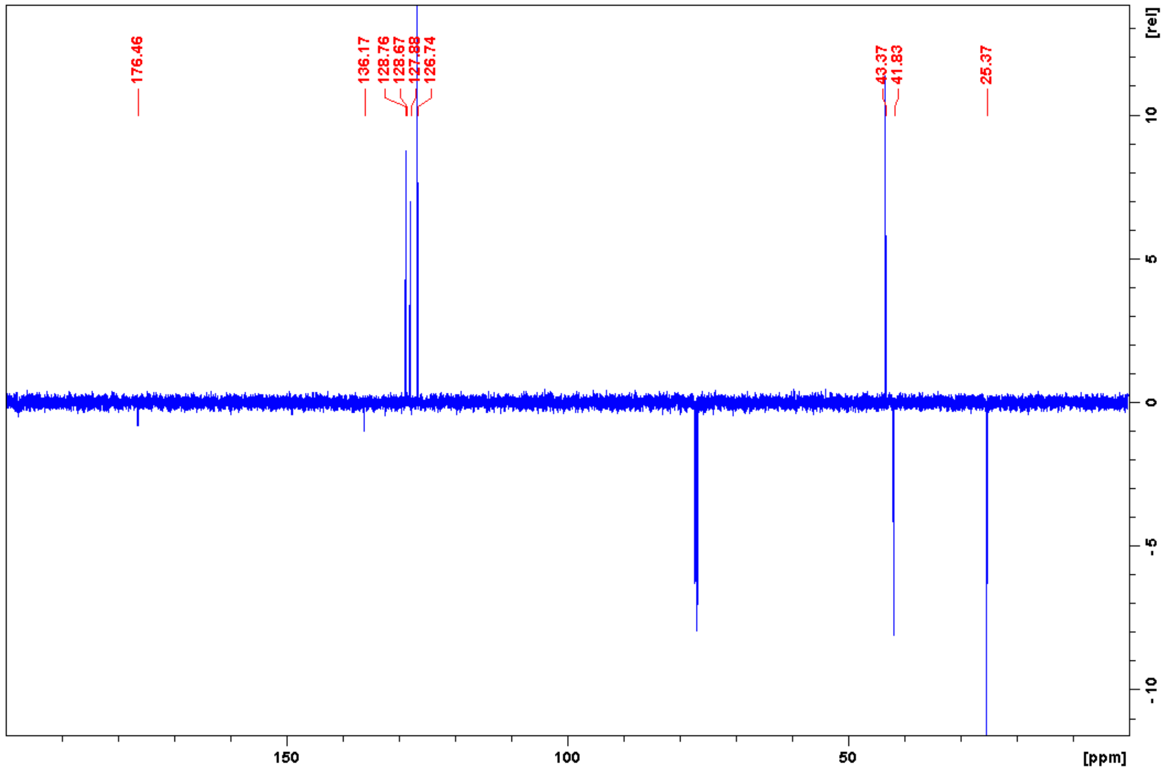

(3a*S,5*S**,6*S**,7a*S**)-2-Benzyl-5-bromo-6-fluorohexahydro-1*H*-isoindole-1,3(2*H*)-dione; (*rac*)-11a**

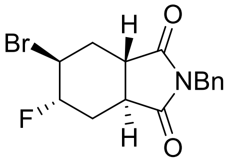

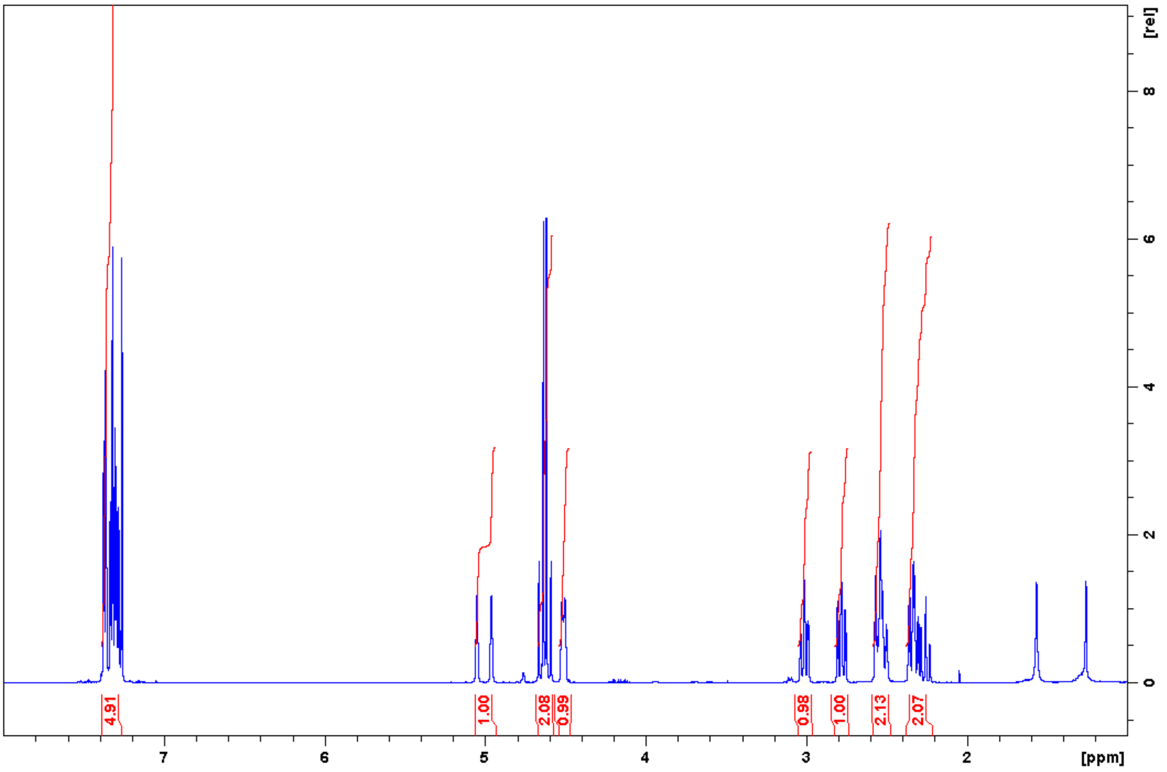

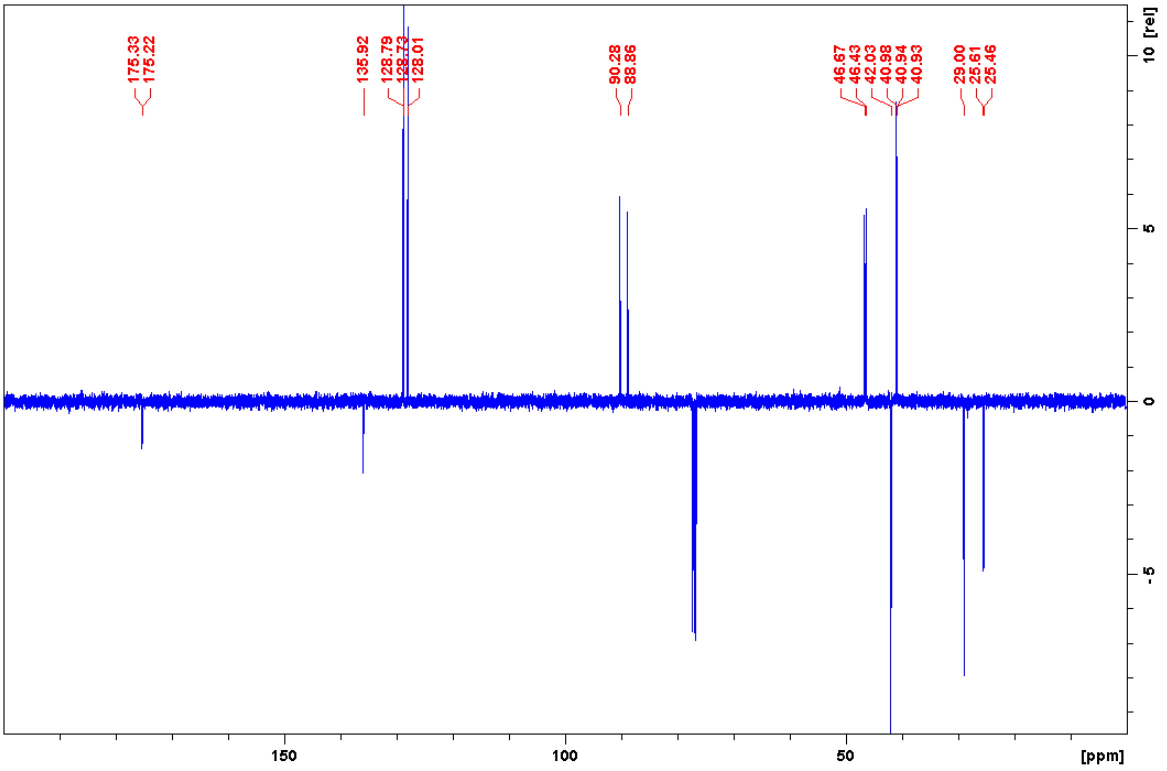

(3a*S,5*S**,6*S**,7a*S**)-2-Benzyl-5-fluoro-6-iodohexahydro-1*H*-isoindole-1,3(2*H*)-dione; (*rac*)-11b**

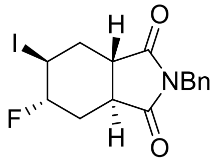

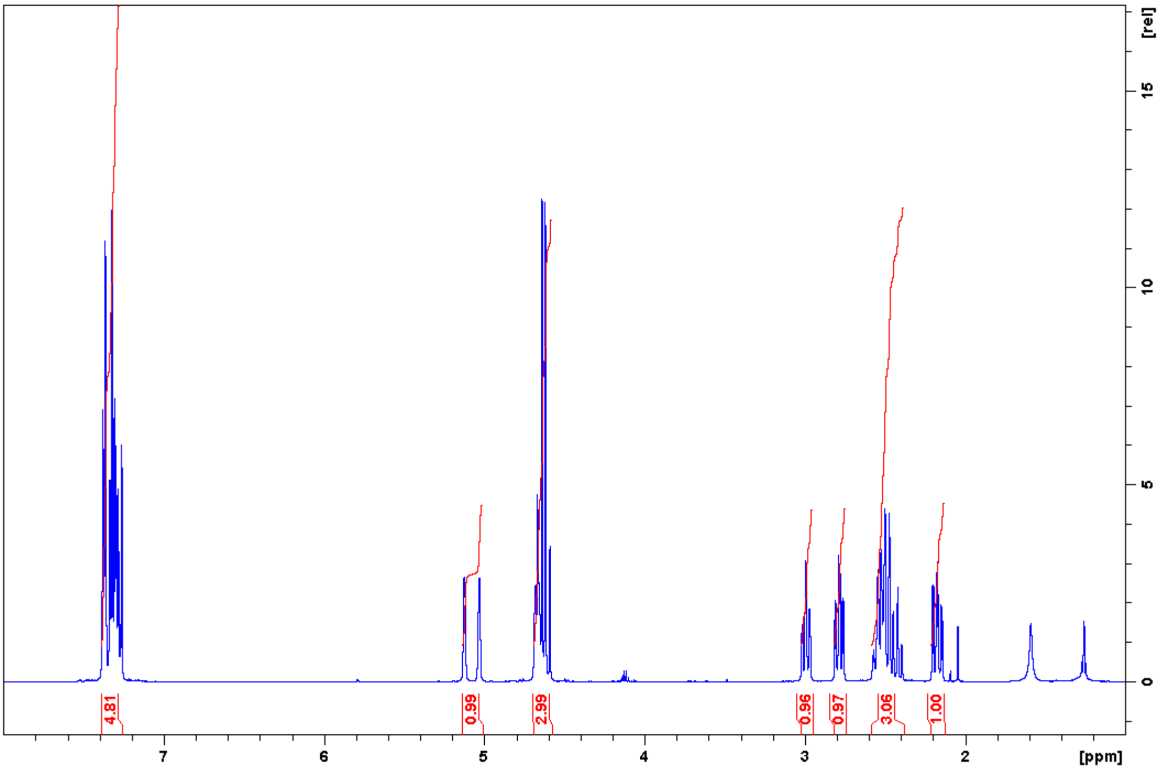

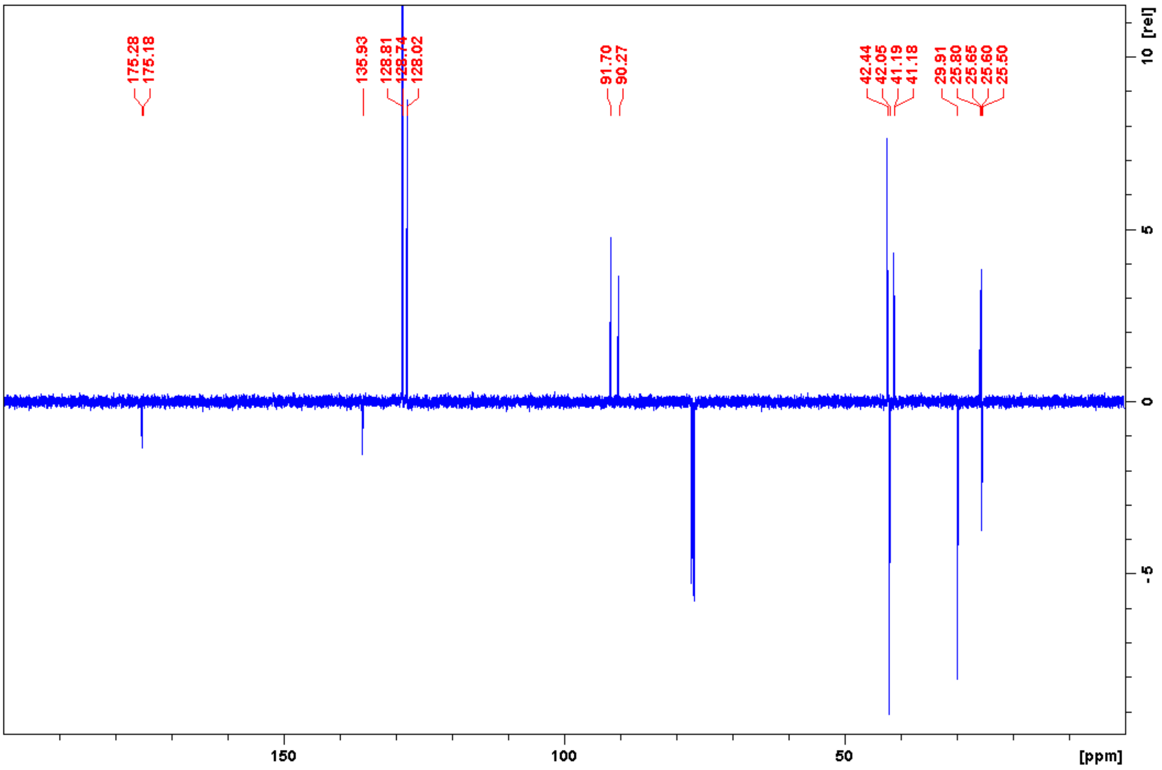

(3a*S,7a*S**)-3a,4,7,7a-Tetrahydro-1*H*-benzo[d]imidazol-2(3H)-one; (*rac*)-13**

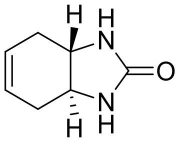

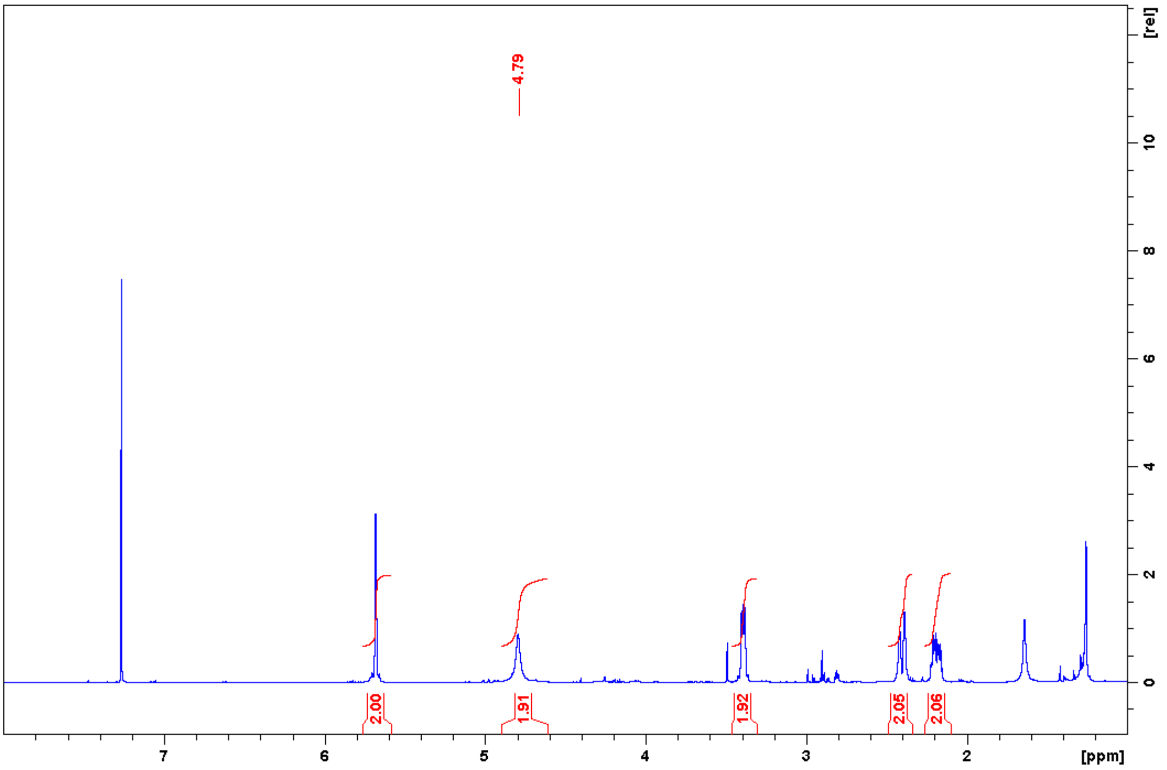

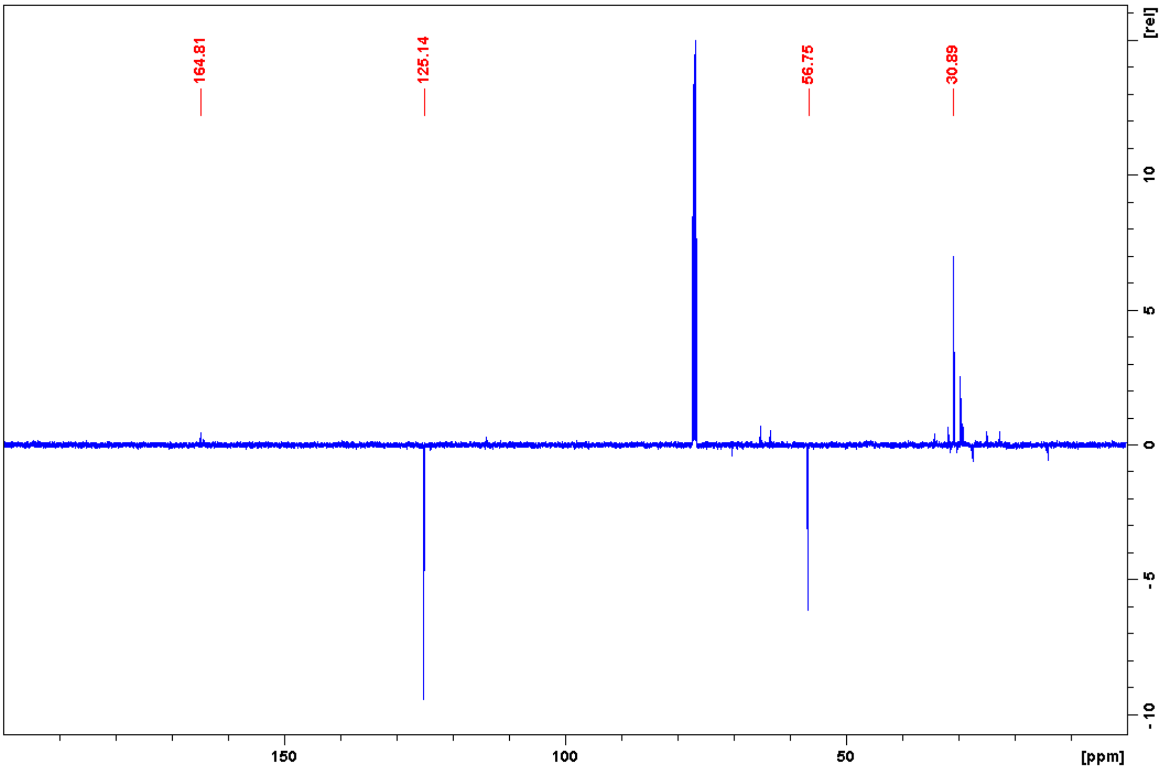

***tert*-Butyl (1*S**,4*S**,5*R**,6*R**)-6-bromo-5-fluoro-3-oxo-2-azabicyclo[2.2.1]heptane-2-carboxylate; (*rac*)-15a**

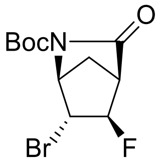

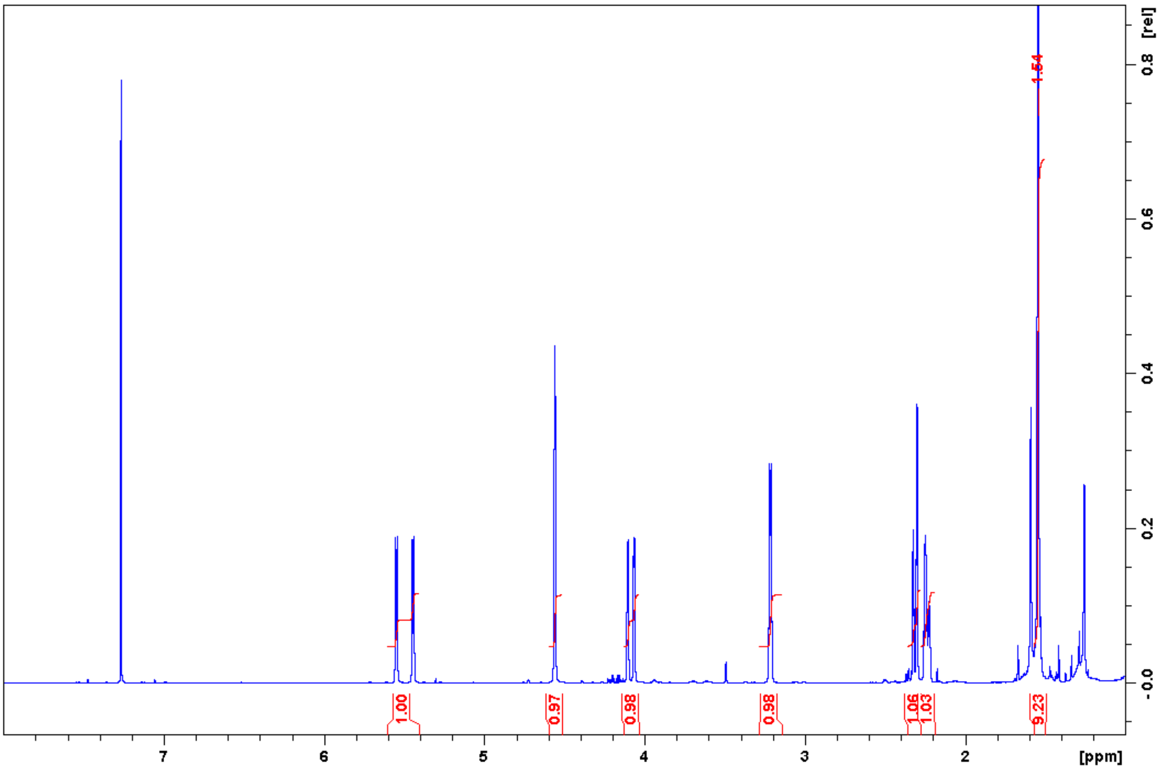

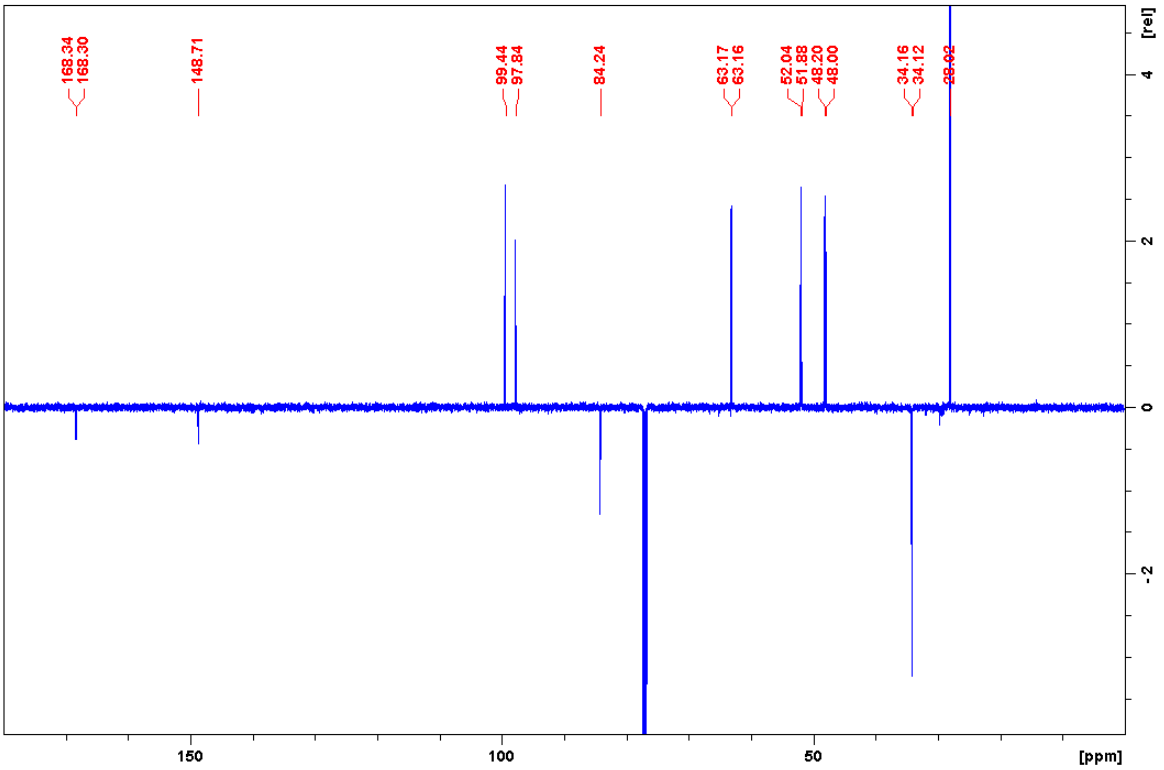

***tert*-Butyl (1*S**,4*S**,5*R**,6*R**)-5-fluoro-6-iodo-3-oxo-2-azabicyclo[2.2.1]heptane-2-carboxylate; (*rac*)-15b**

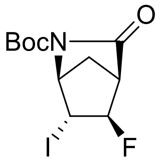

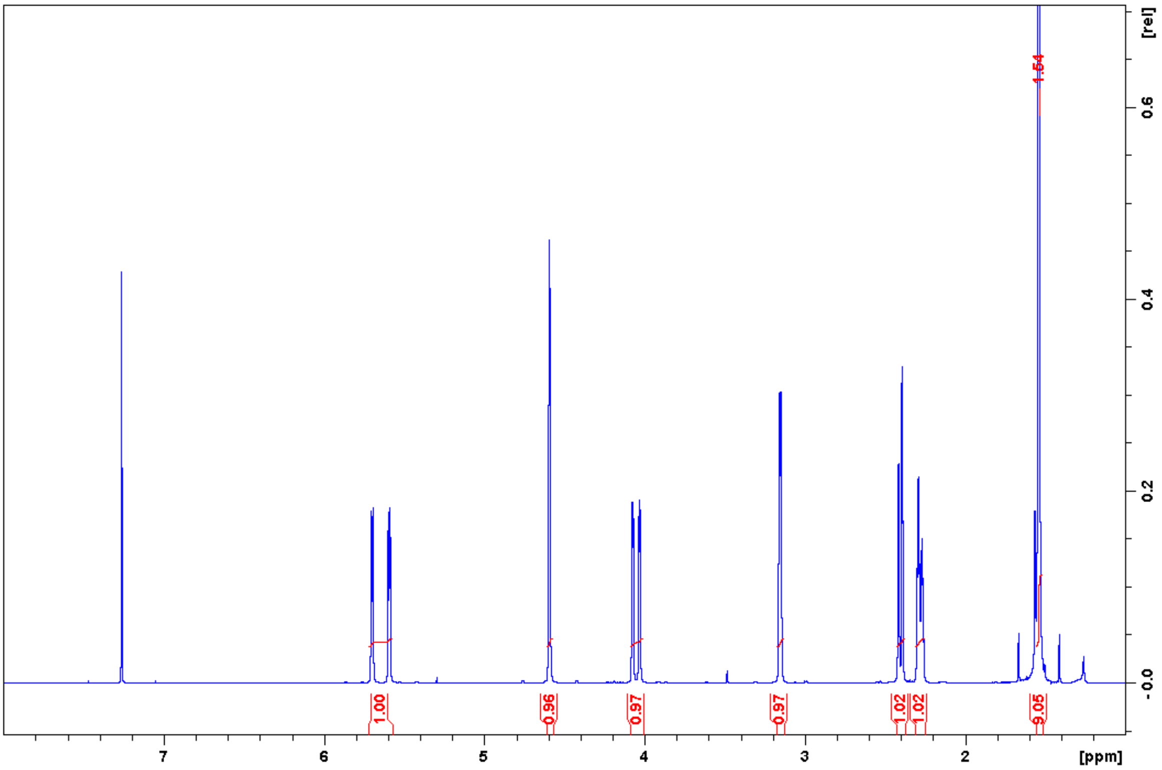

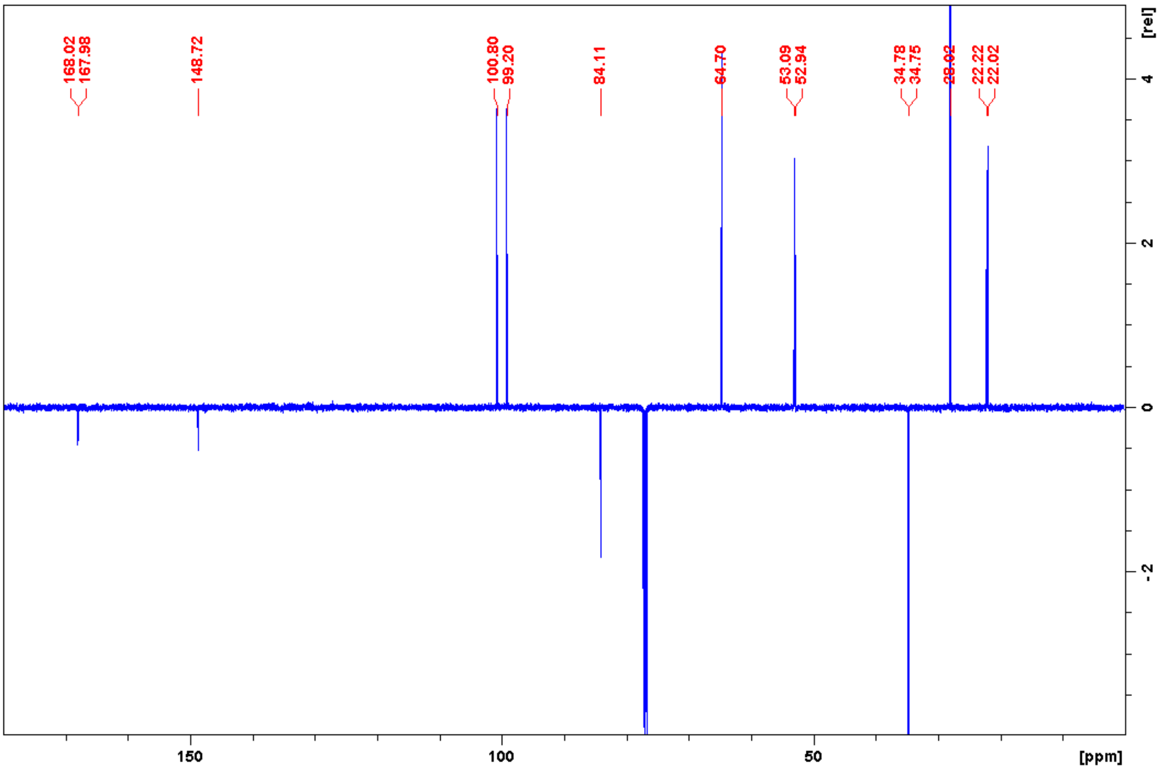

(3*R,3a*R**,5*S**,6*S**,6a*S**,7*R**)-Methyl 6-bromo-2-oxohexahydro-2*H*-3,5-methanocyclopenta[b]furan-7-carboxylate; (*rac*)-17a**

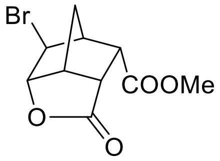

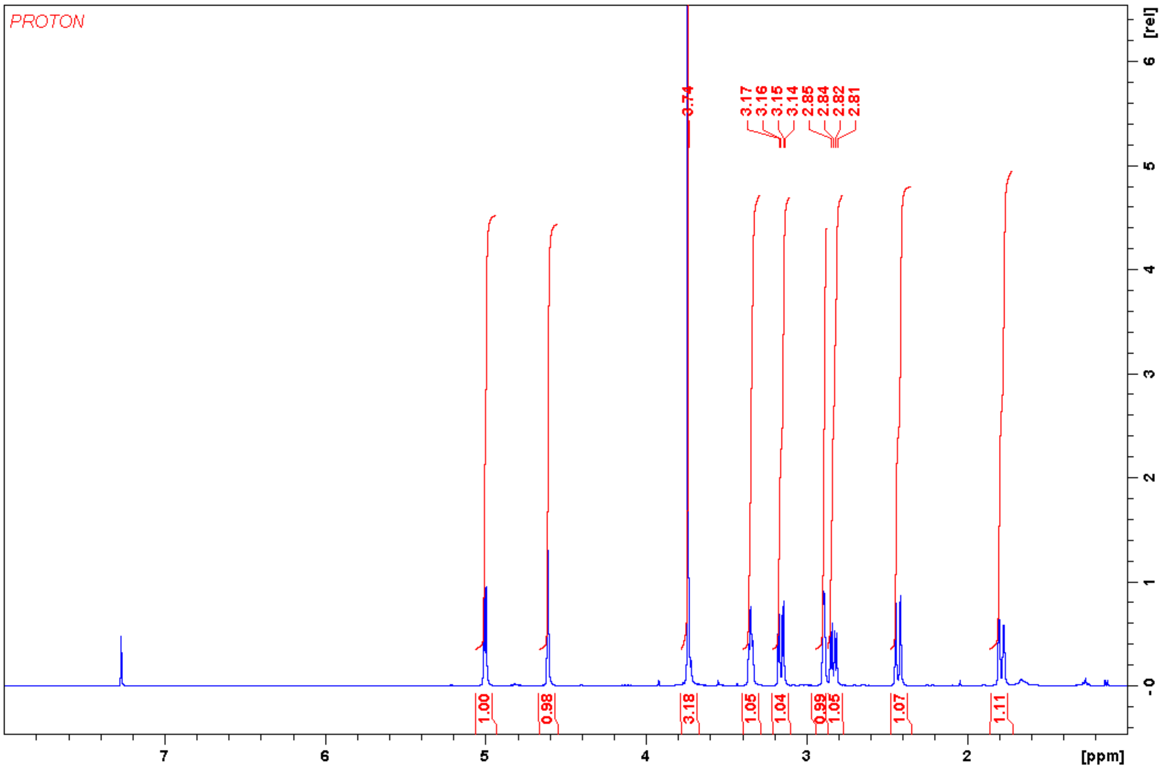

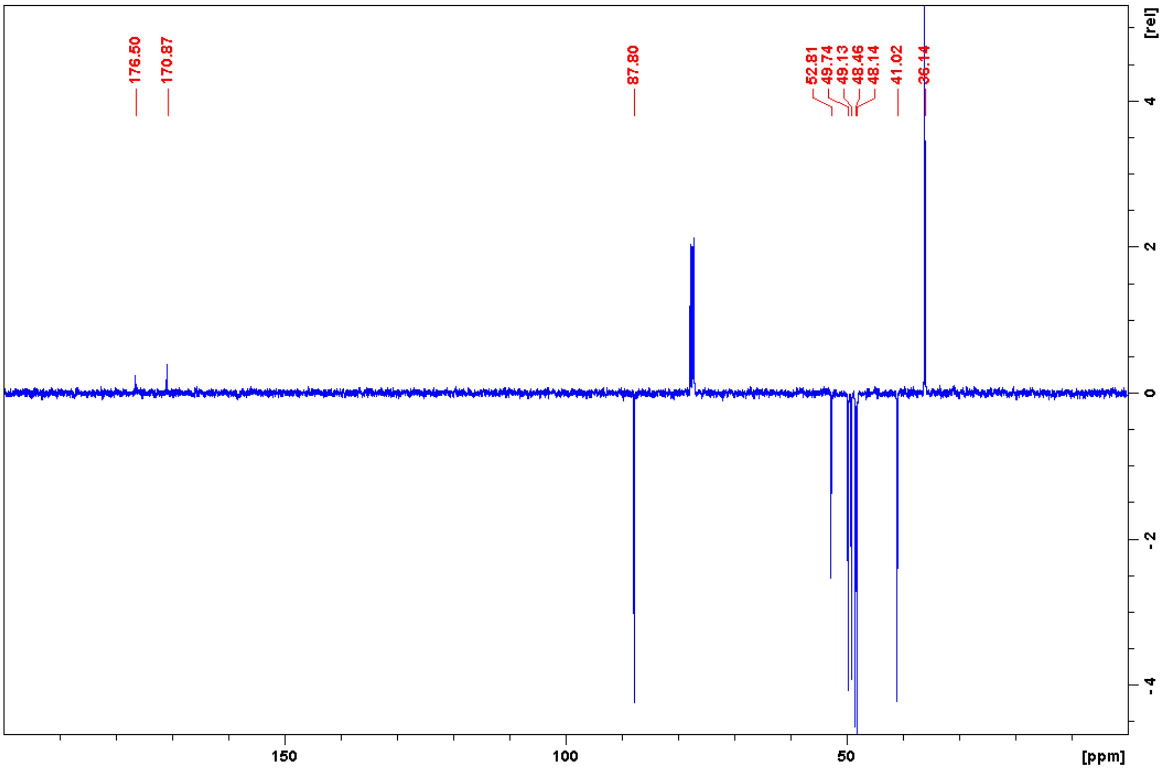

(3*R,3a*R**,5*S**,6*S**,6a*S**,7*R**)-Methyl 6-iodo-2-oxohexahydro-2*H*-3,5-methanocyclopenta[b]furan-7-carboxylate; (*rac*)-17b**

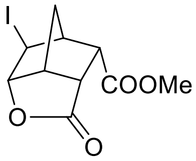

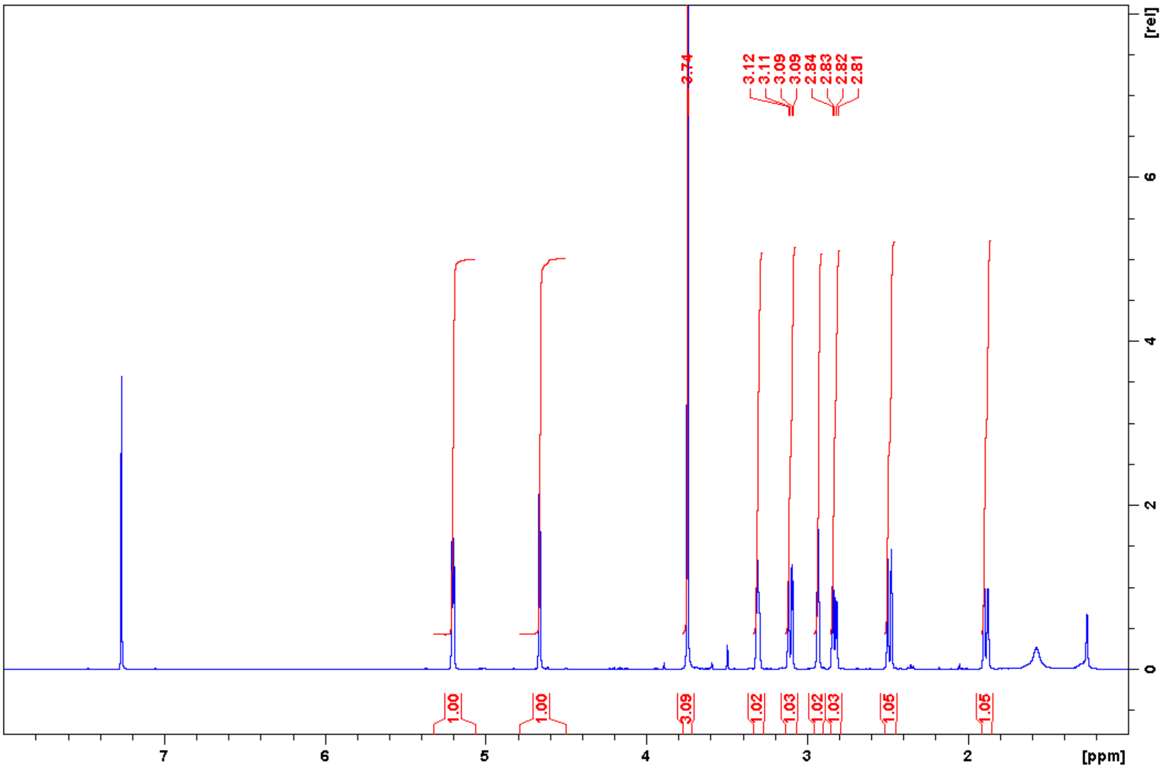

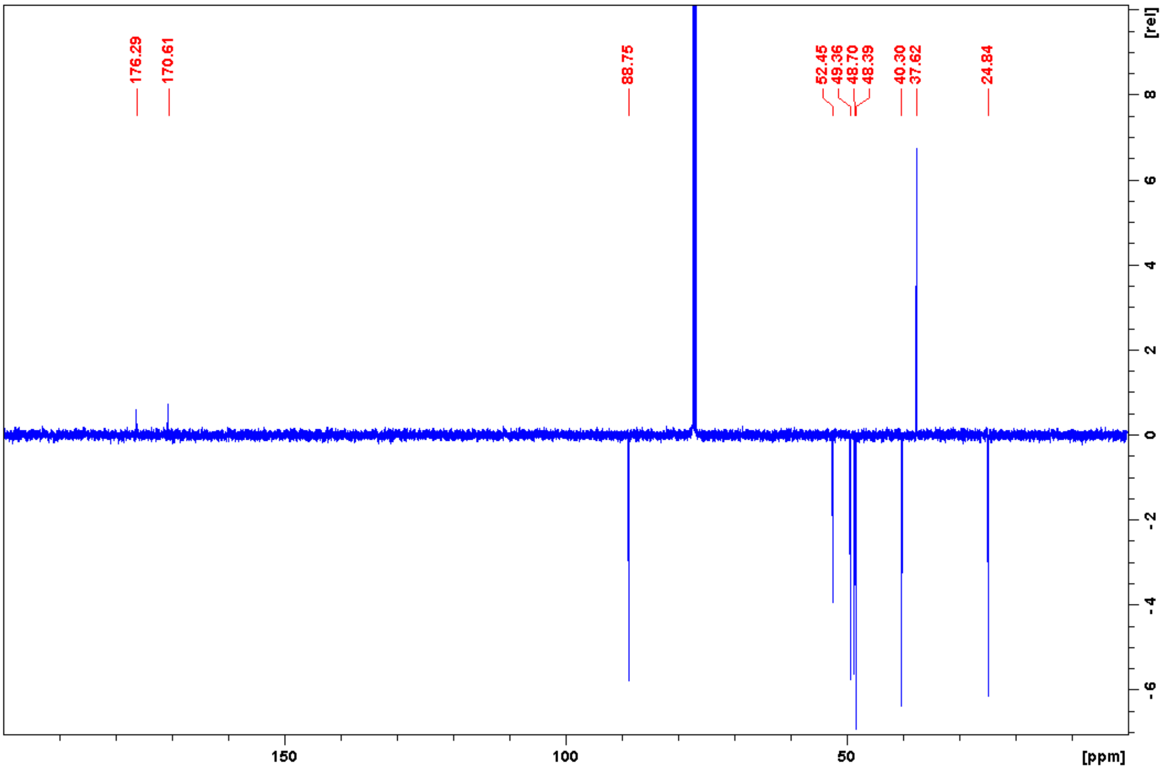

(3a*R,4*R**,5*R**,7*R**,7a*S**,8*R**)-2-Benzyl-8-bromo-5-fluorohexahydro-1*H*-4,7-methanoisoindole-1,3(2*H*)-dione; (*rac*)-20a**

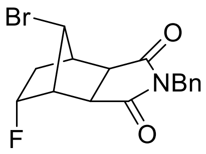

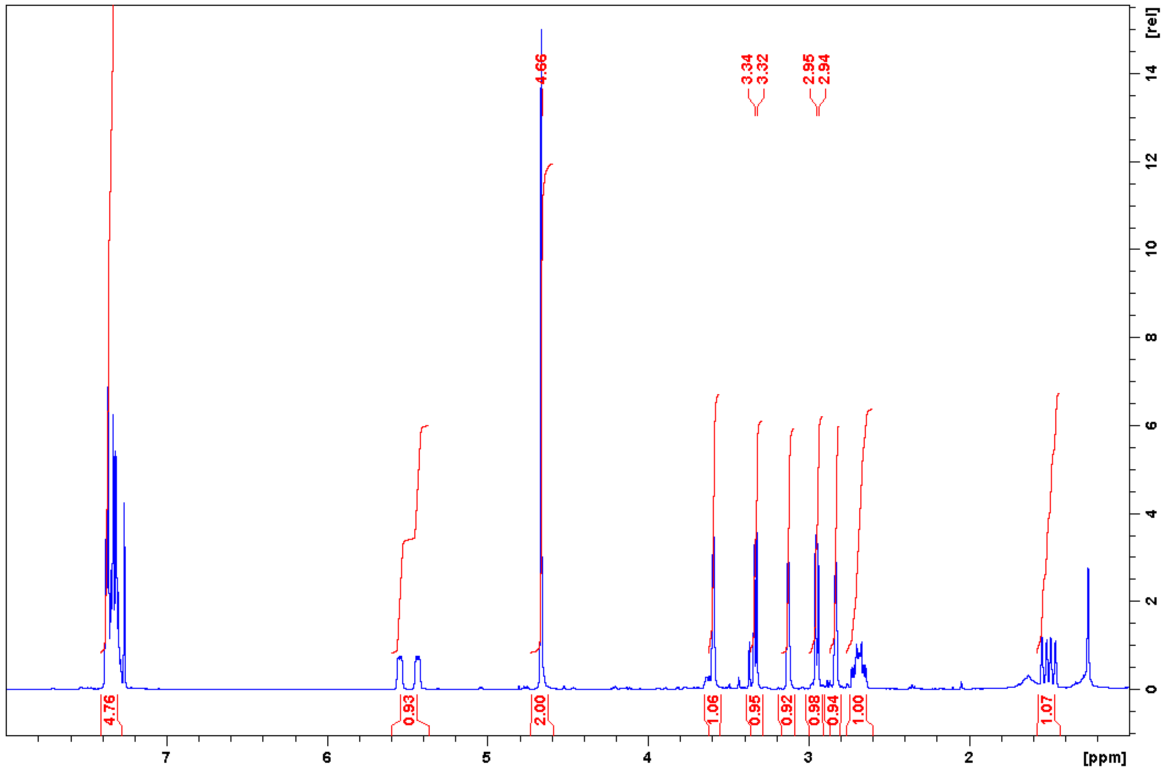

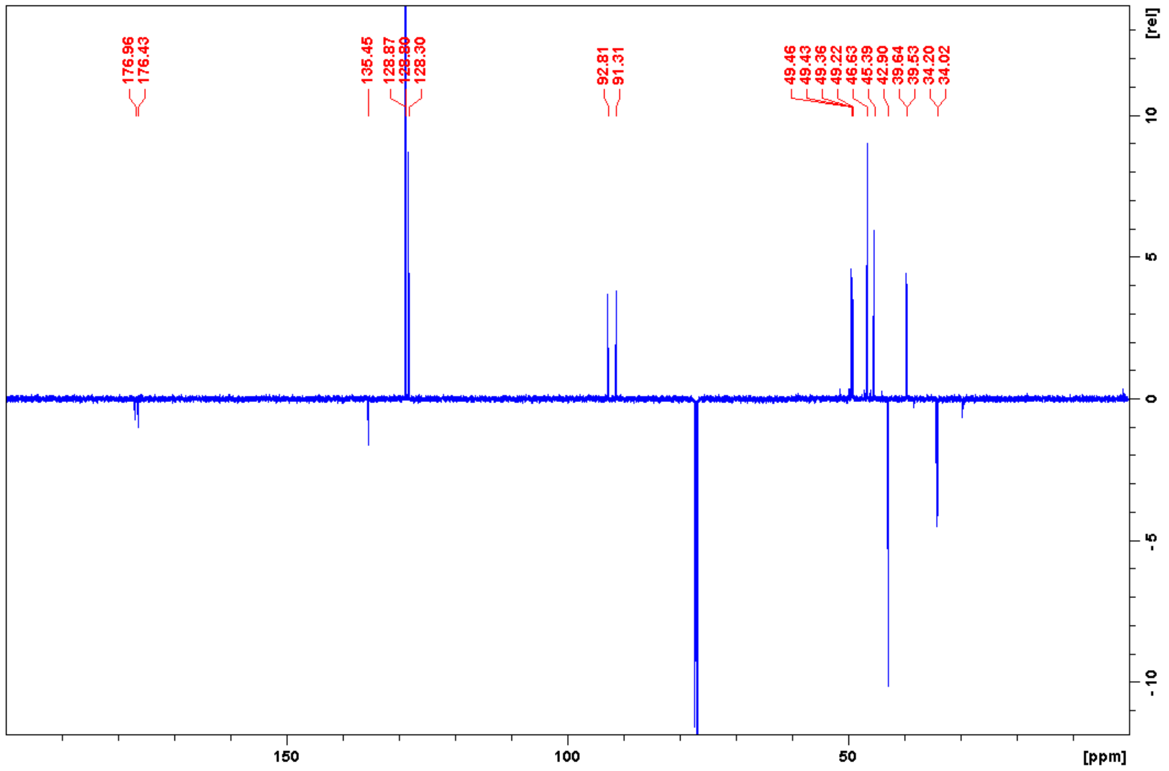

(3a*R,4*R**,5*S**,7*R**,7a*S**,8*R**)-2-Benzyl-8-bromo-5-fluorohexahydro-1*H*-4,7-methanoisoindole-1,3(2*H*)-dione;** (*rac*)-**21a**

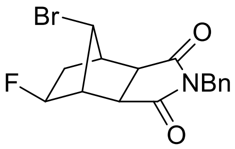

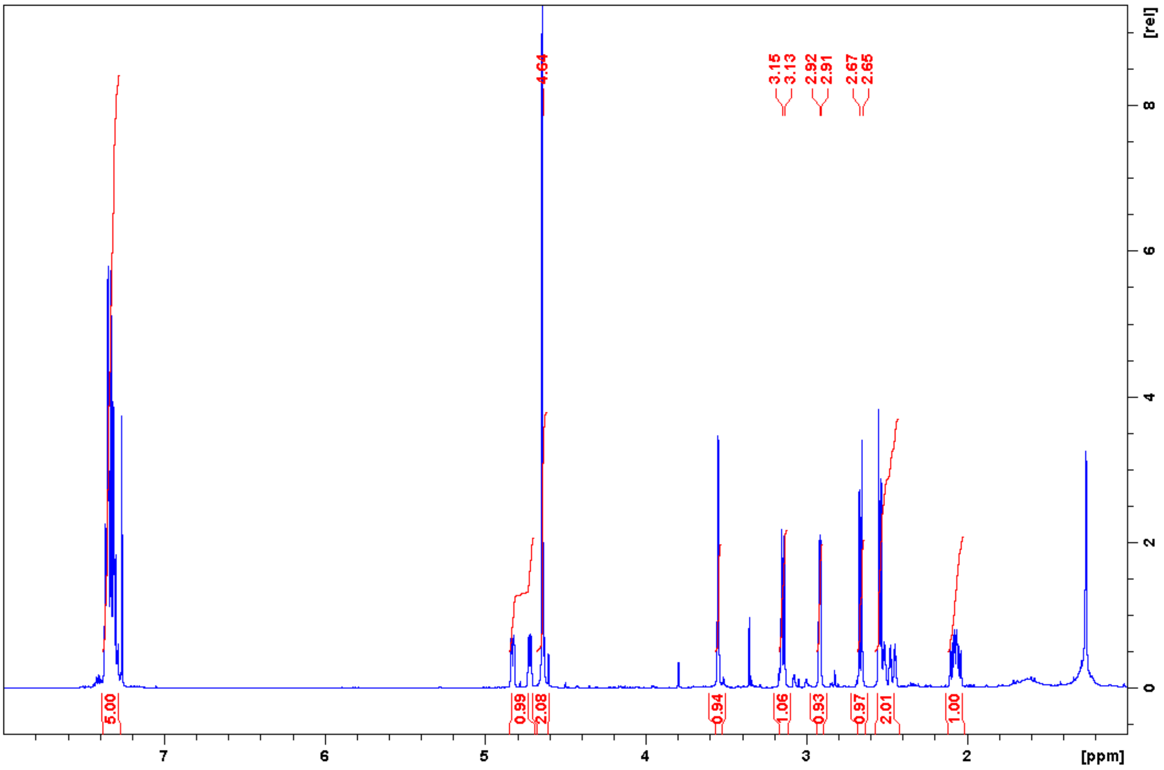

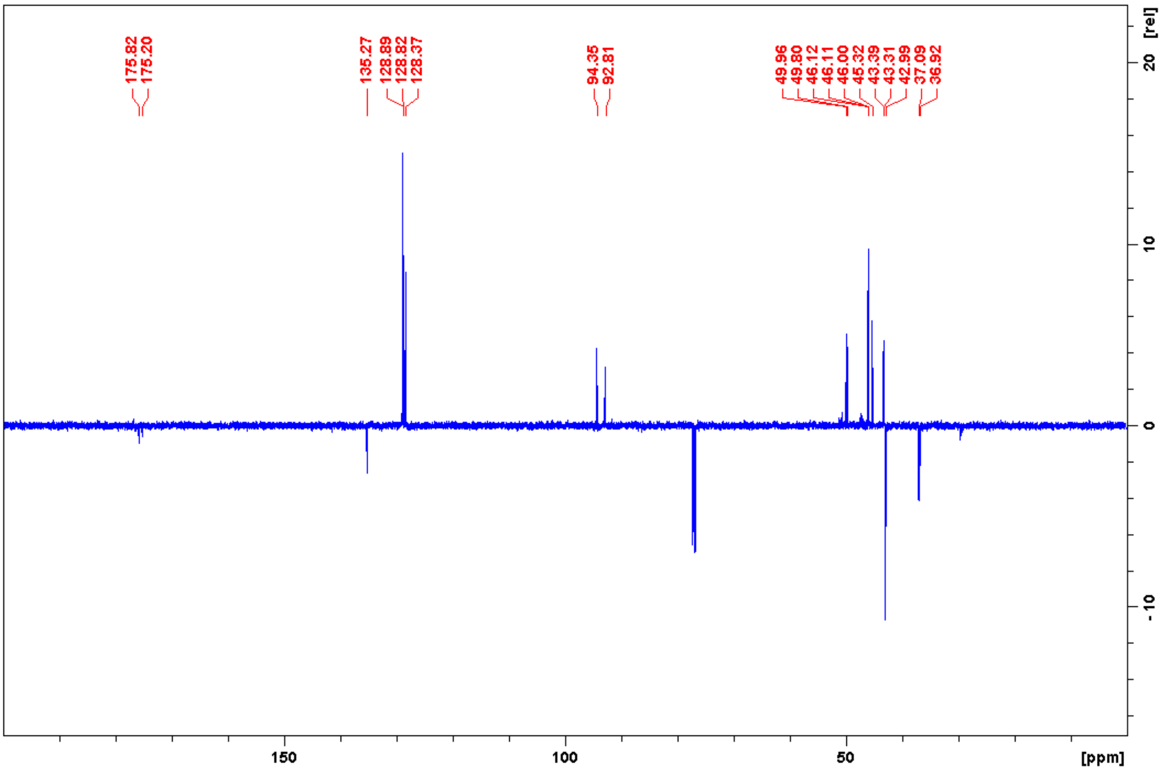

(3a*S,4*R**,5*R**,6*S**,7*S**,7a*R**)-2-Benzyl-5,6-dibromohexahydro-1*H*-4,7-methanoisoindole-1,3(2*H*)-dione; 22**

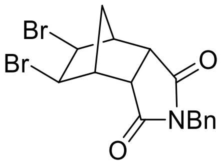

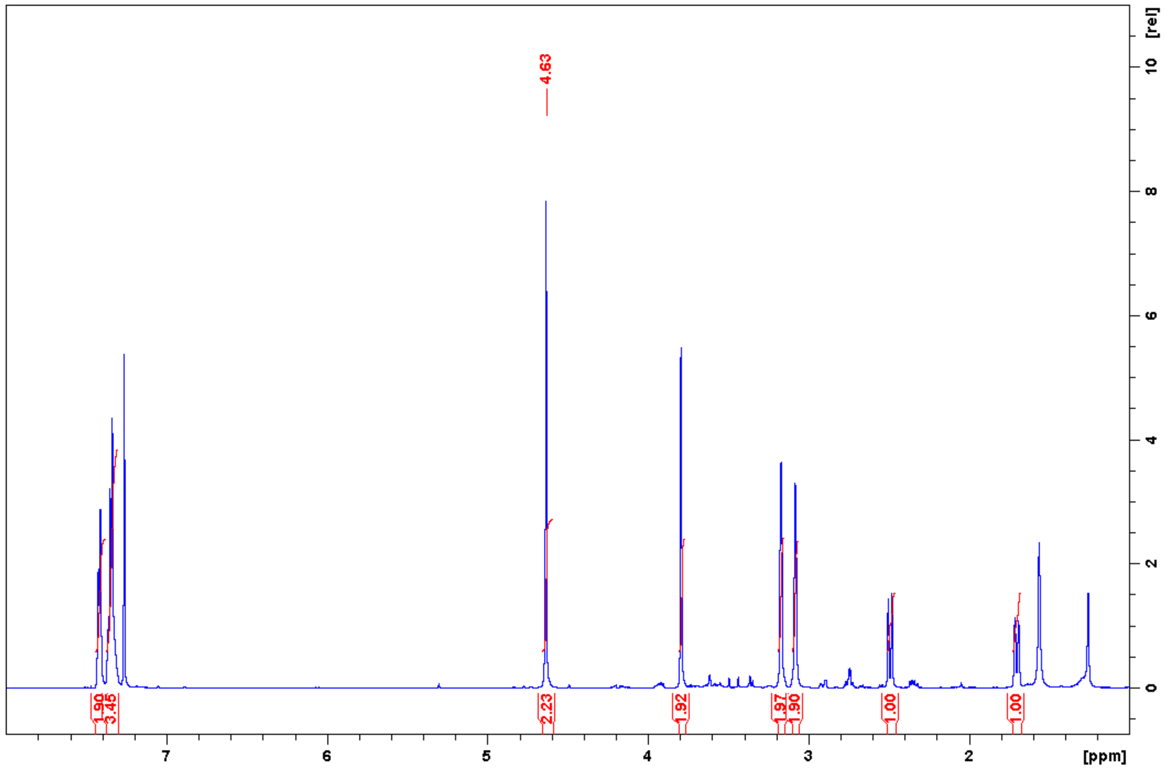

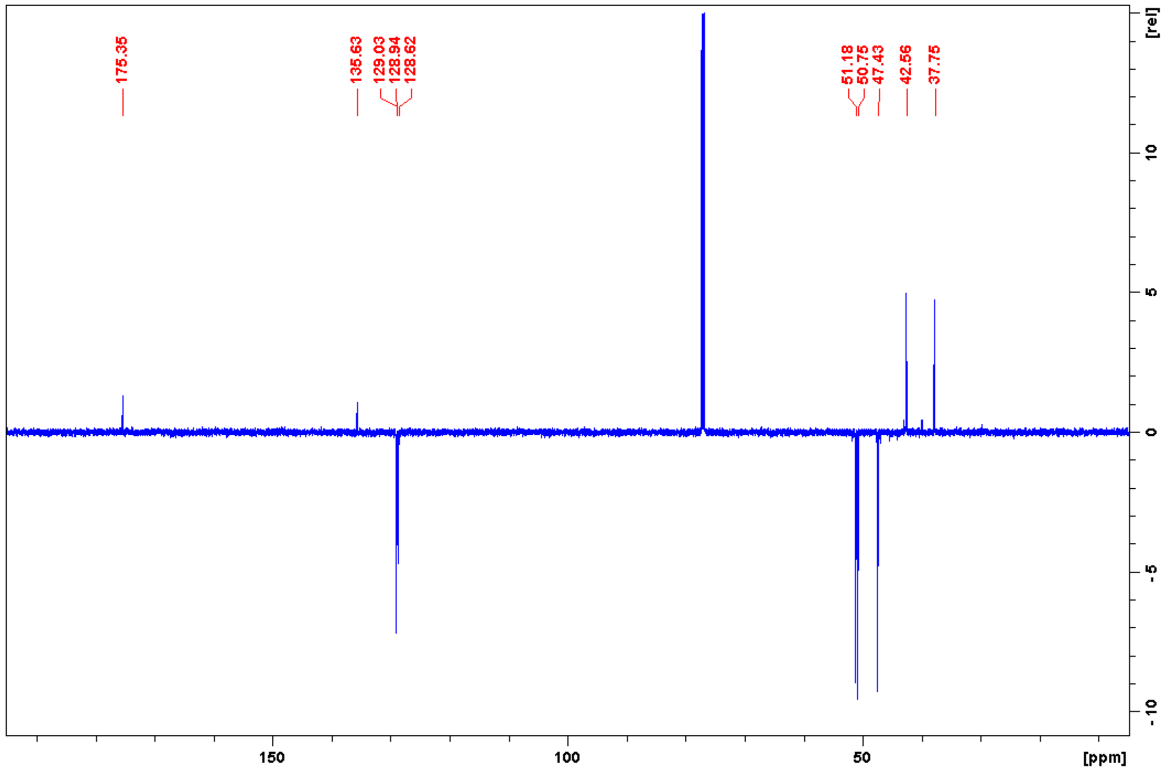

(3a*R,4*R**,5*R**,7*R**,7a*S**,8*R**)-2-Benzyl-5-fluoro-8-iodohexahydro-1*H*-4,7-methanoisoindole-1,3(2*H*)-dione; (*rac*)-20b**

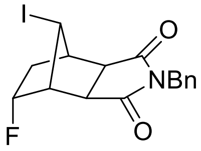

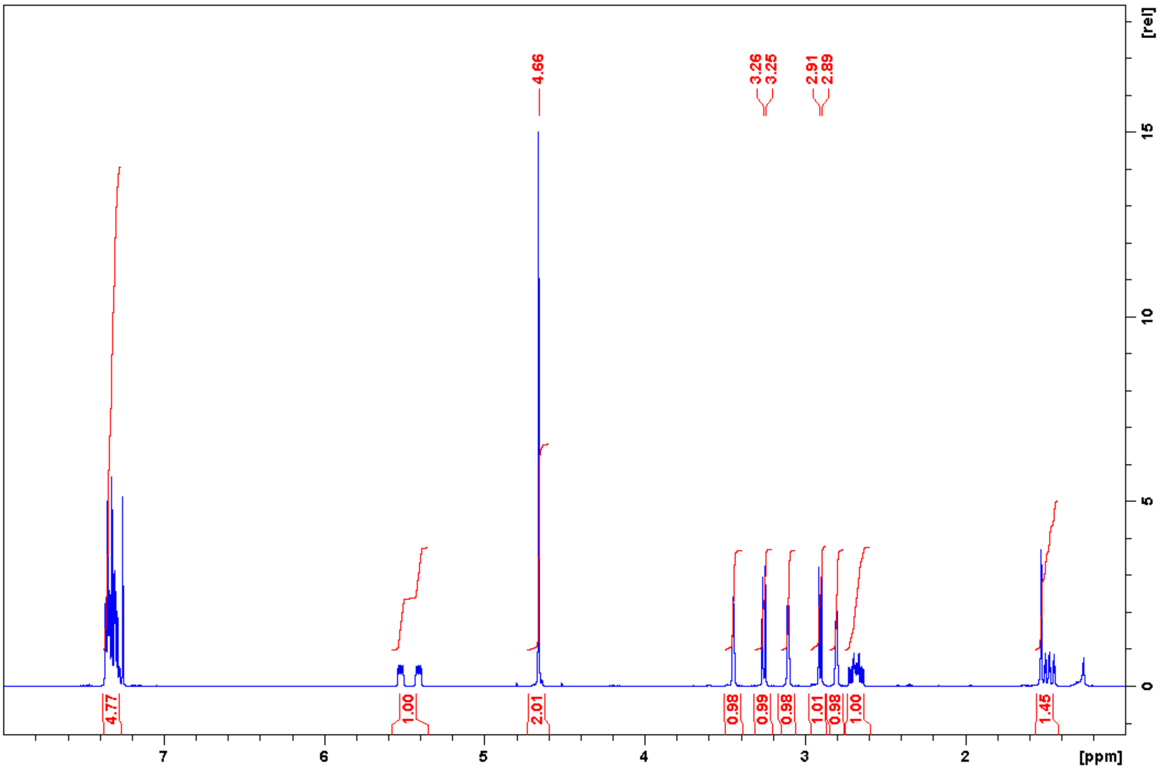

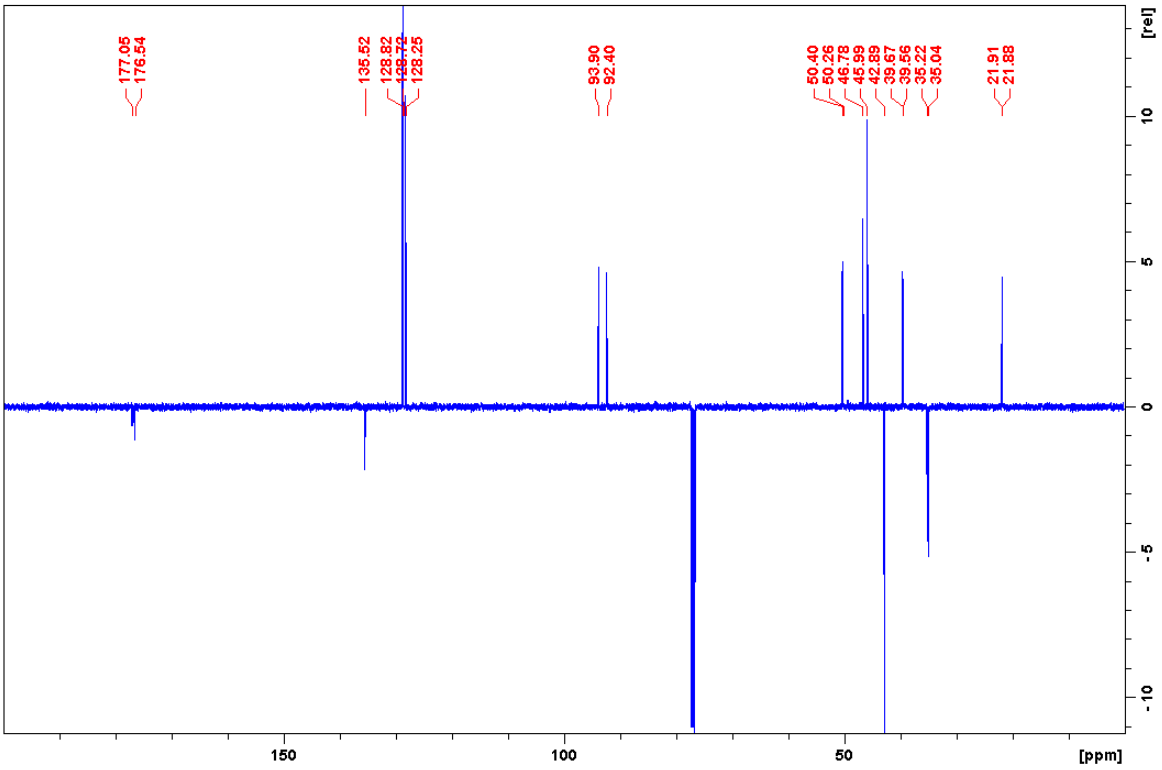

(3a*R,4*R**,5*R**,7*R**,7a*S**,8*R**)-2-Benzyl-8-chloro-5-fluorohexahydro-1*H*-4,7-methanoisoindole-1,3(2*H*)-dione; (*rac*)-20c**

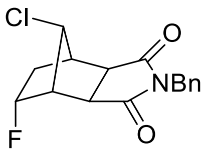

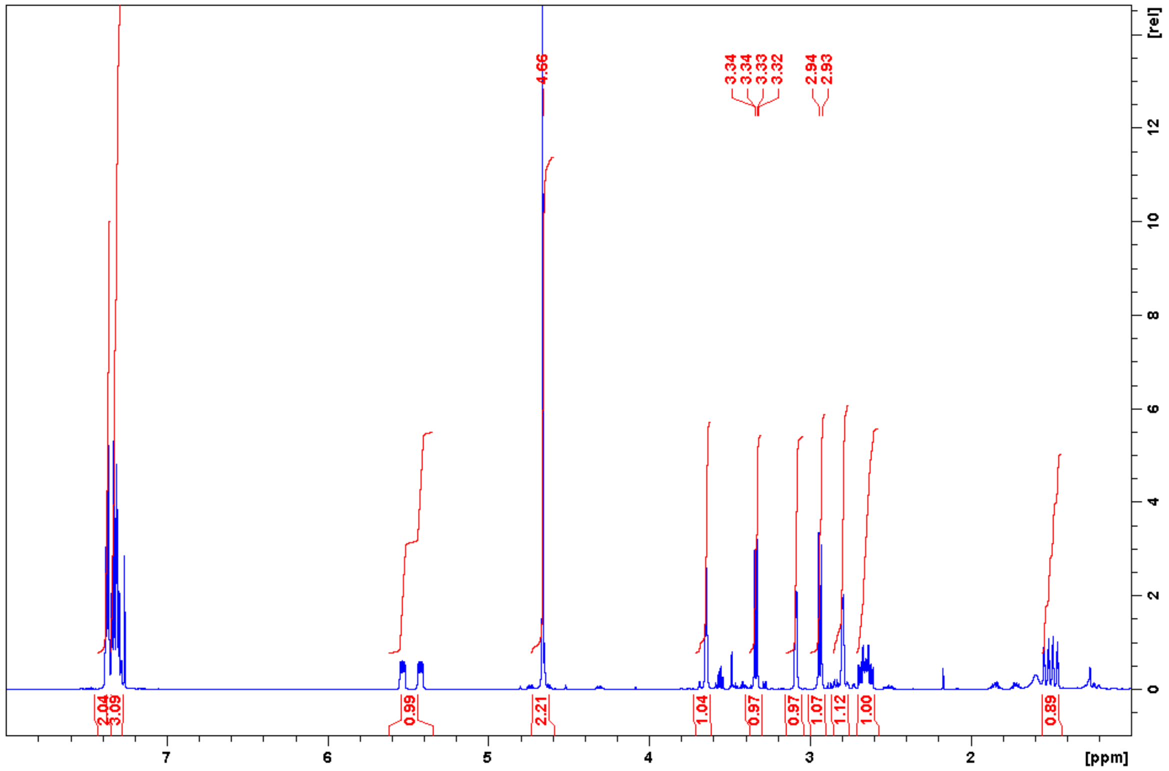

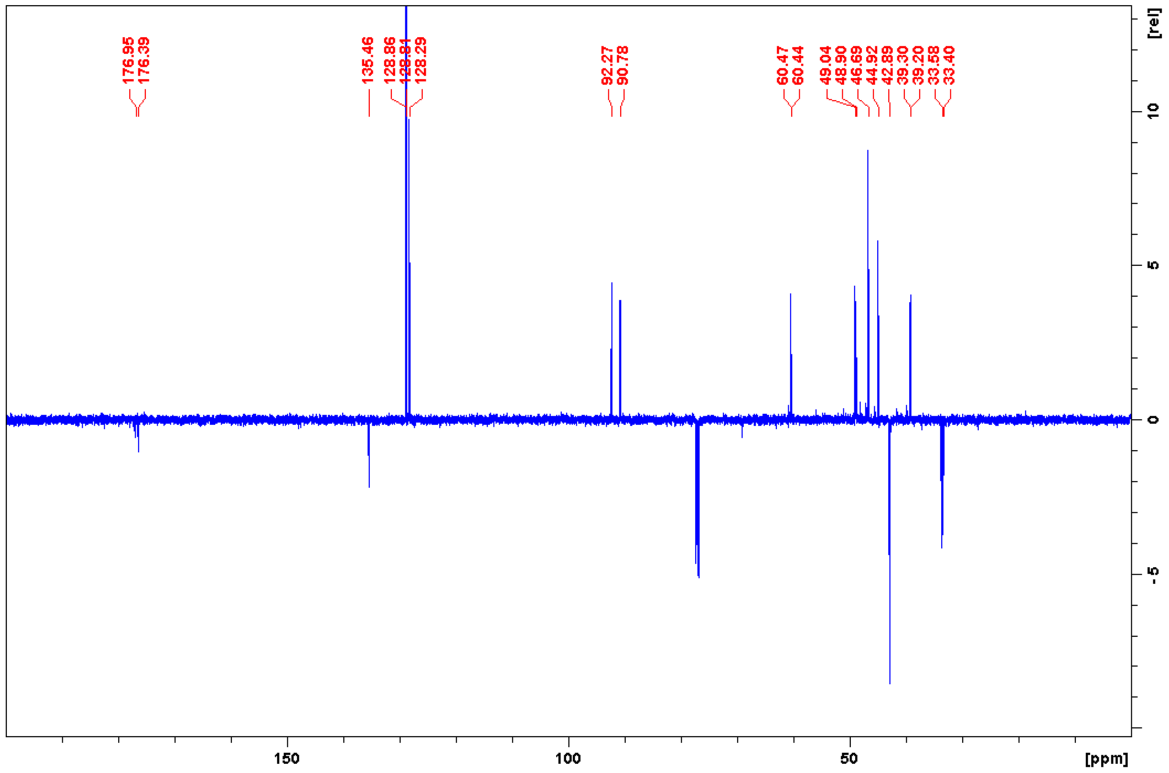

(3a*R,4*R**,5*R**,7*R**,7a*S**,8*R**)-8-Bromo-5-fluoro-2-methylhexahydro-1*H*-4,7-methanoisoindole-1,3(2*H*)-dione; (*rac*)-25a**

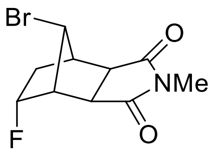

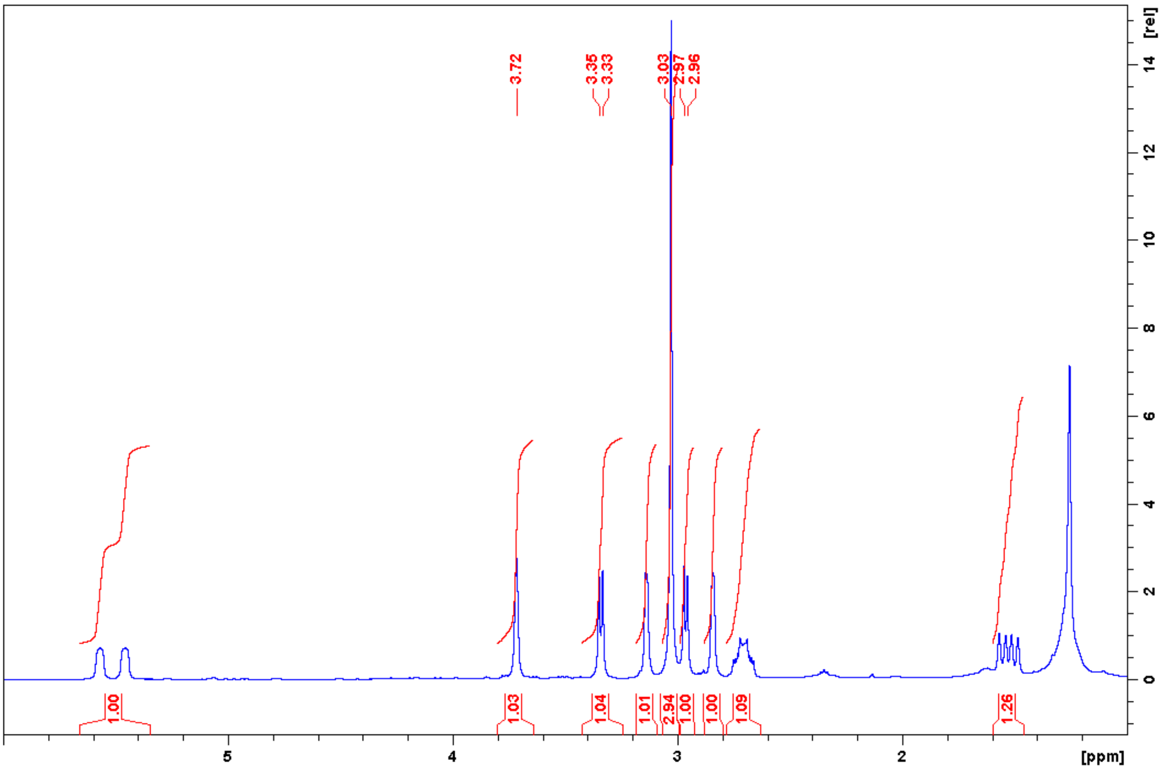

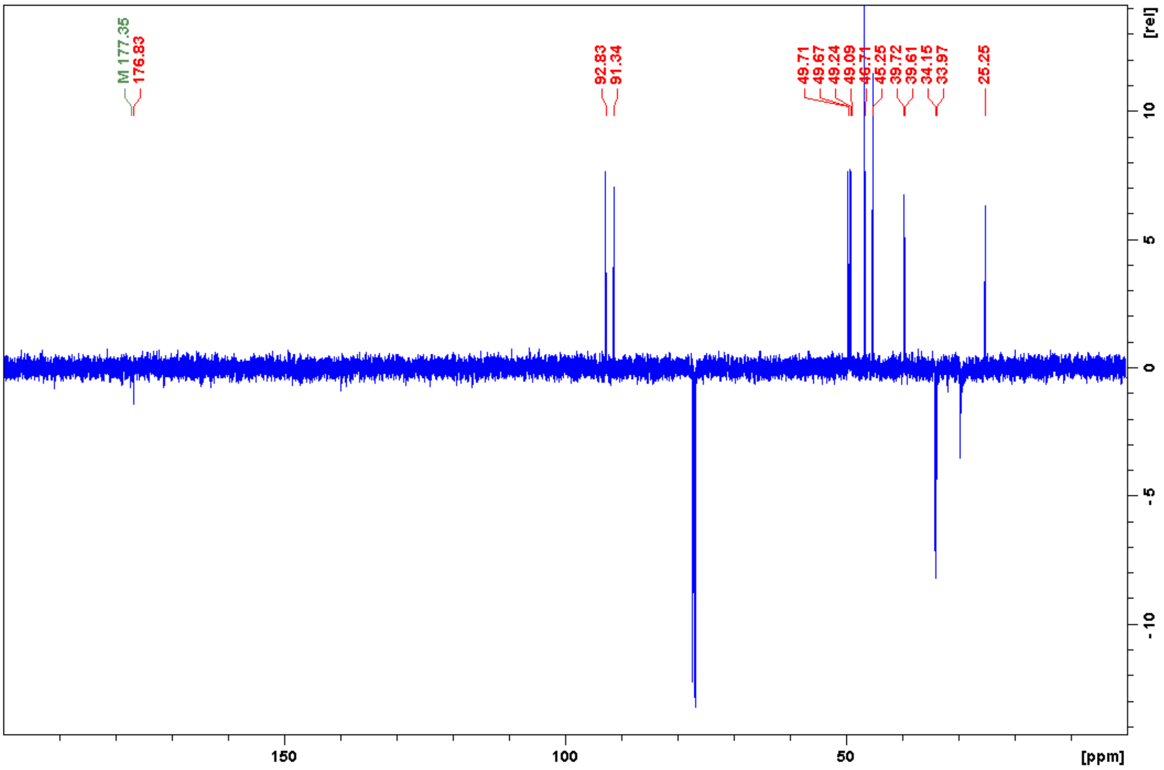

(3a*R,4*R**,5*R**,7*R**,7a*S**,8*R**)-5-Fluoro-8-iodo-2-methylhexahydro-1*H*-4,7-methanoisoindole-1,3(2*H*)-dione; (*rac*)-25b**

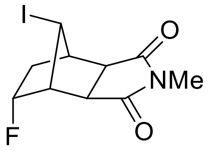

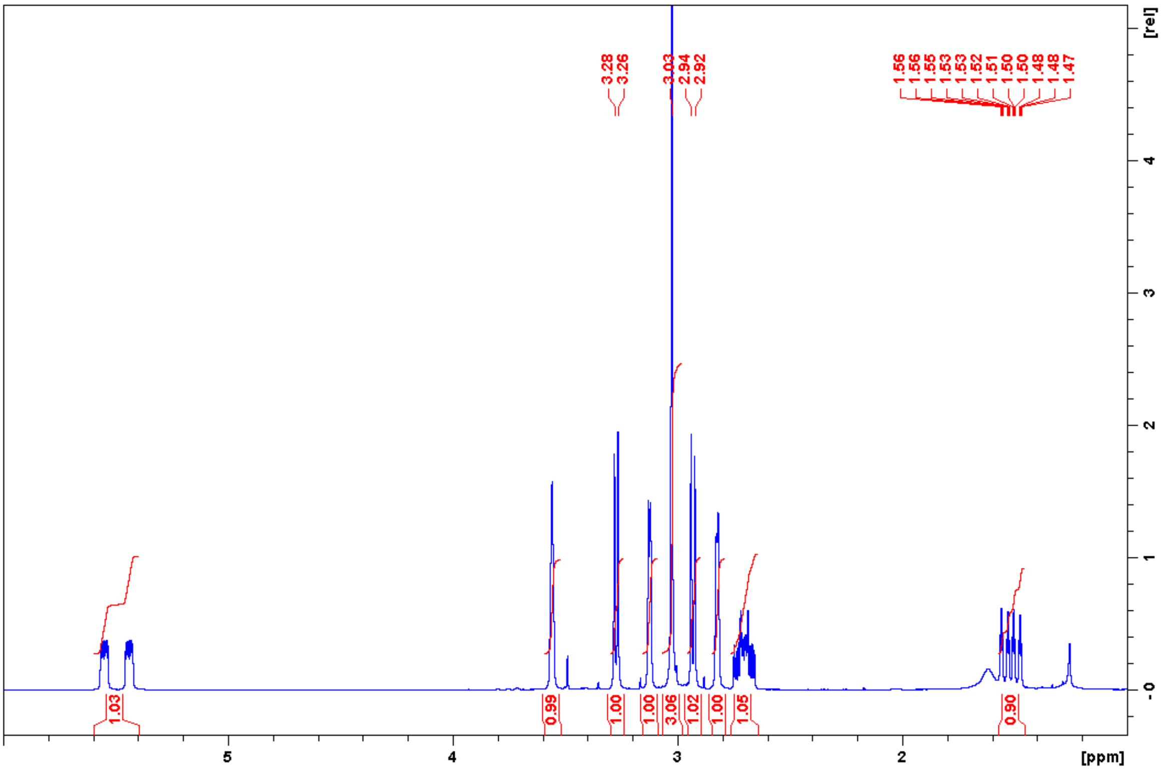

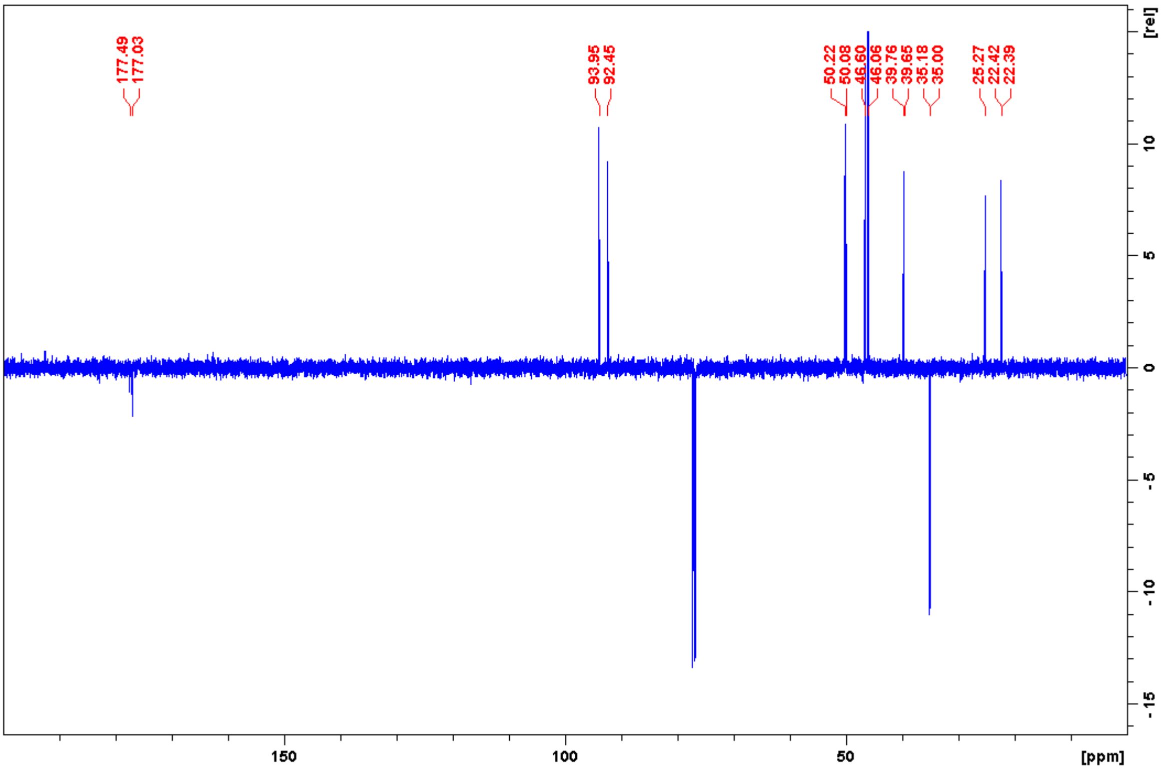

(3a*S,4*R**,5*R**,6*S**,7*S**,7a*R**)-5,6-Dibromo-2-methylhexahydro-1*H*-4,7-methanoisoindole-1,3(2*H*)-dione; 26**

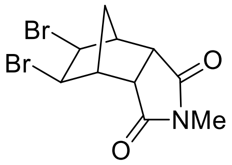

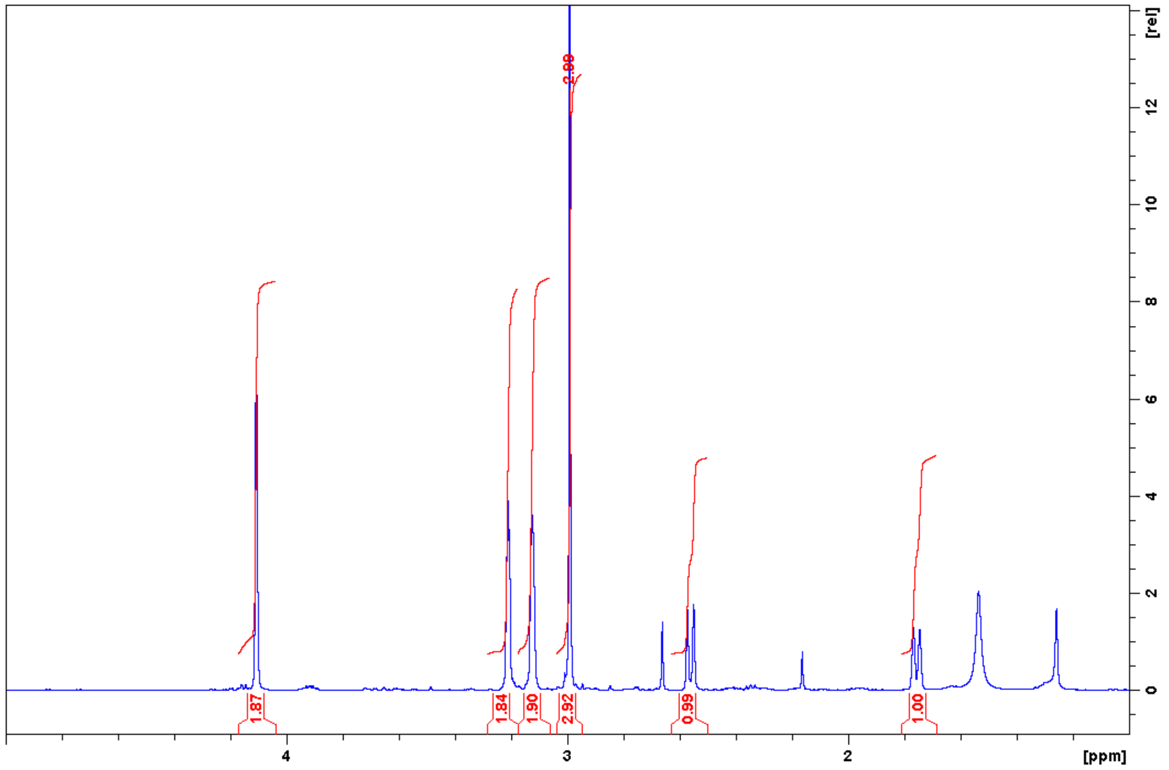

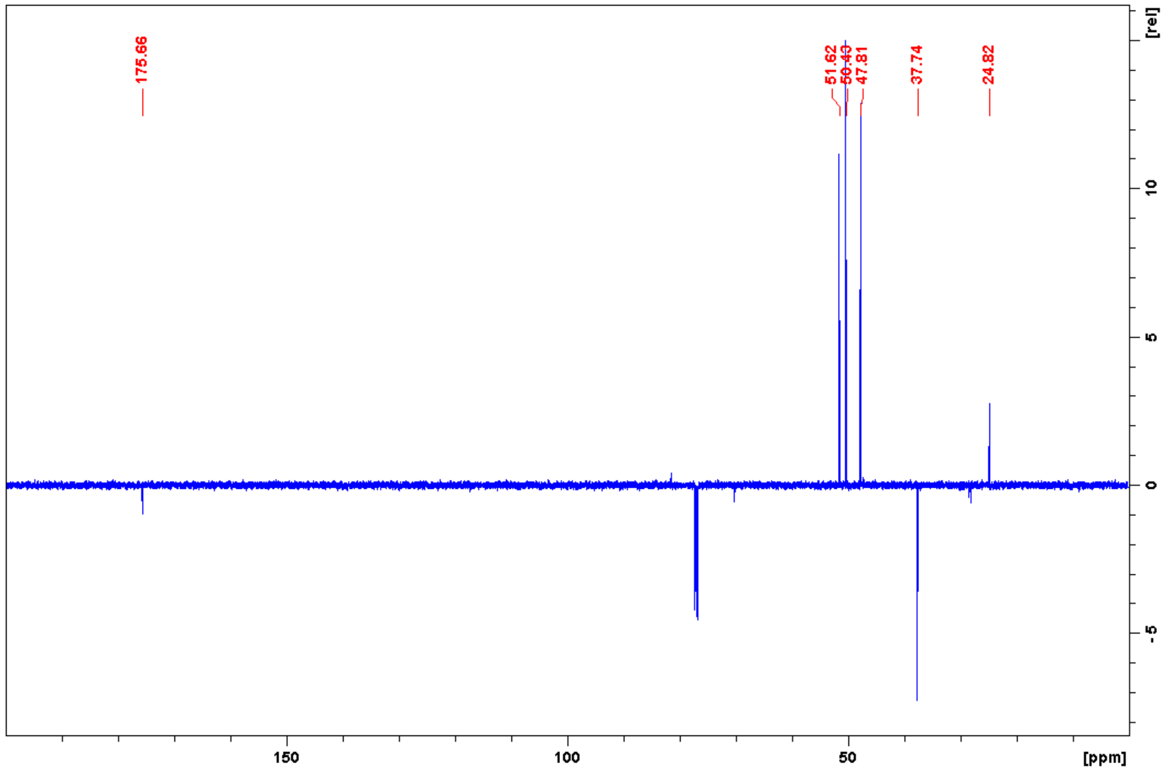

Dimethyl (1*S,2*R**,4*R**,5*R**)-4-fluorobicyclo[3.1.0]hexane-1,2-dicarboxylate; (*rac*)-27**

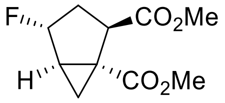

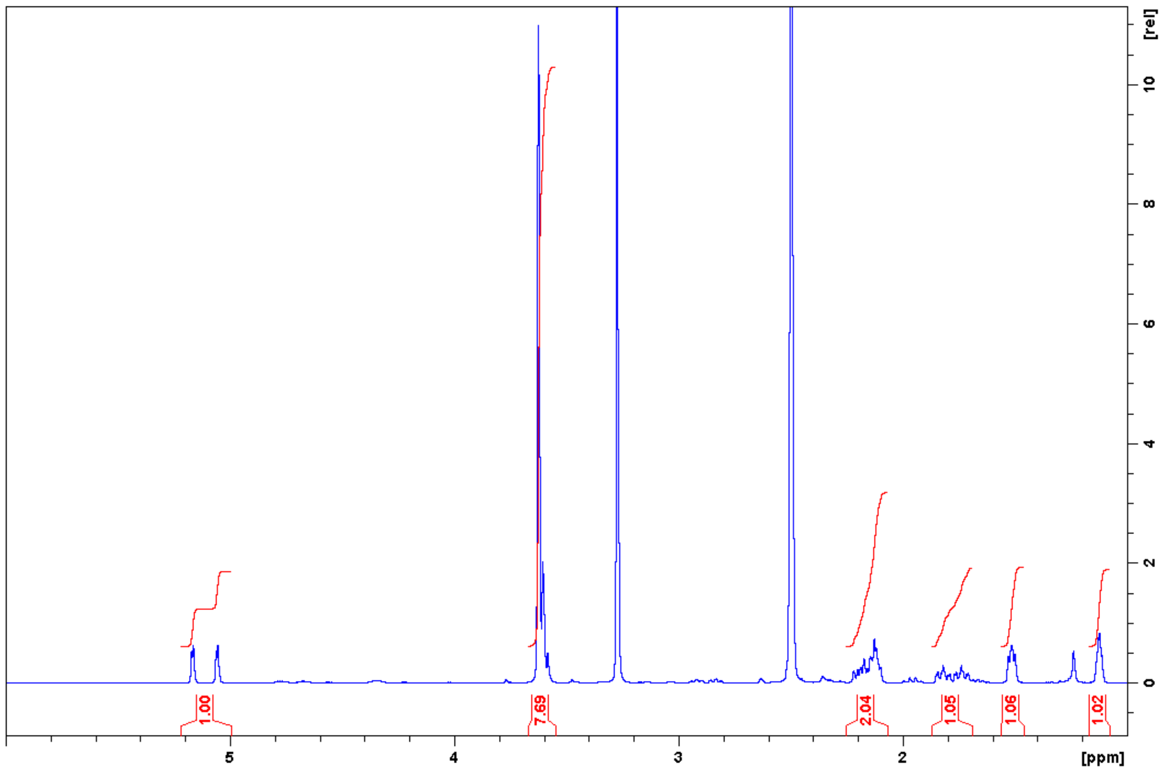

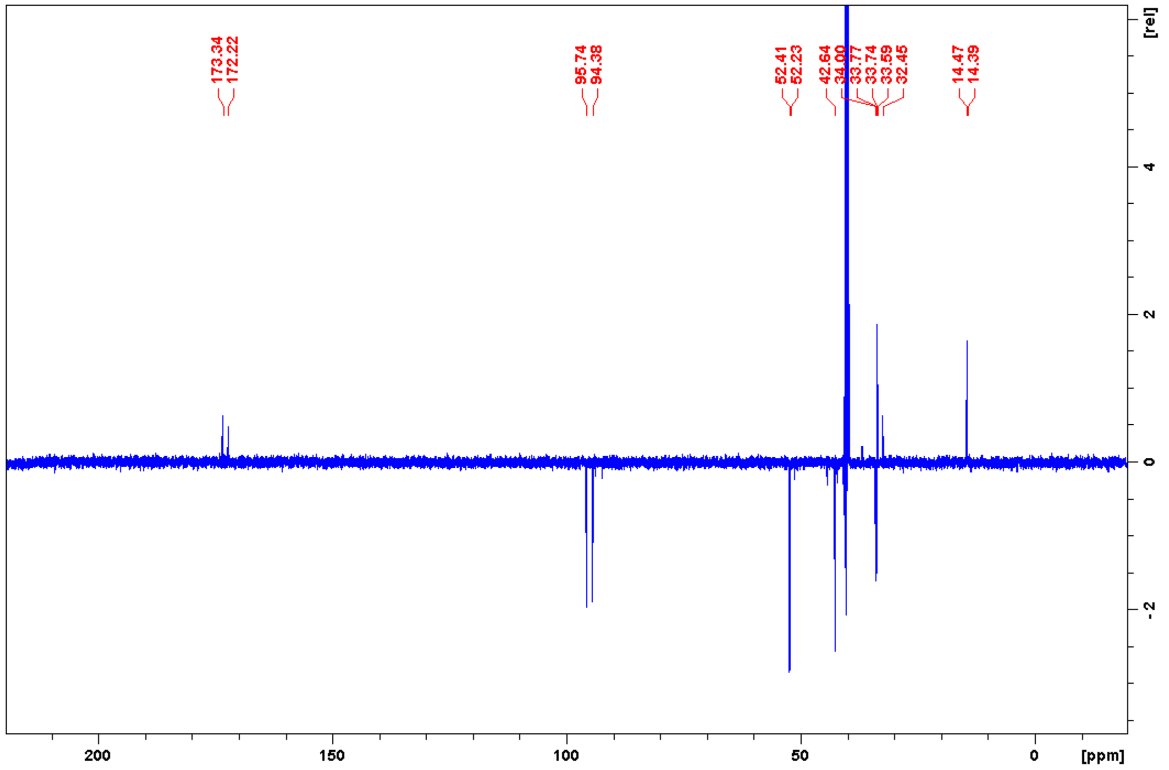

(3a*S,5*R**,5a*R**,6a*S**)-2-Benzyl-5-fluorotetrahydrocyclopropa[1,5]cyclopenta[1,2-c]pyrrole-1,3(2*H*,3a*H*)-dione; (*rac*)-28**

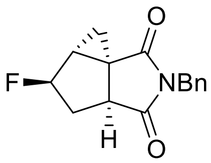

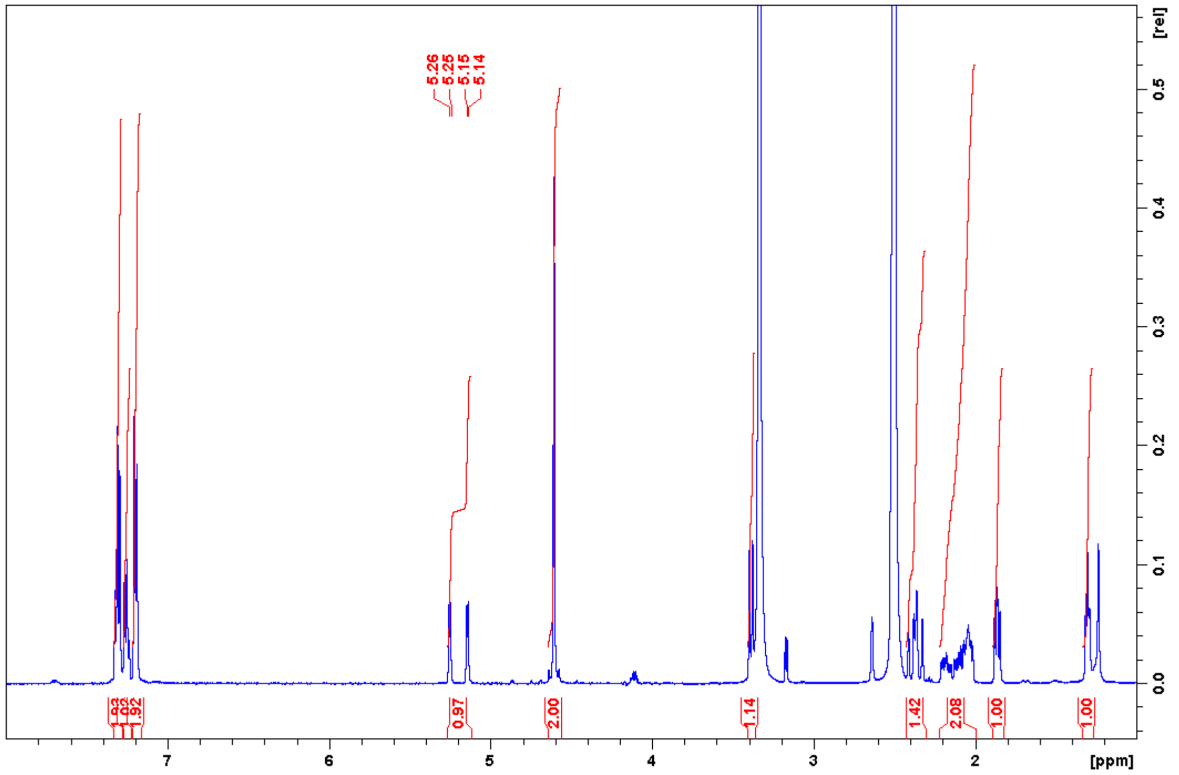

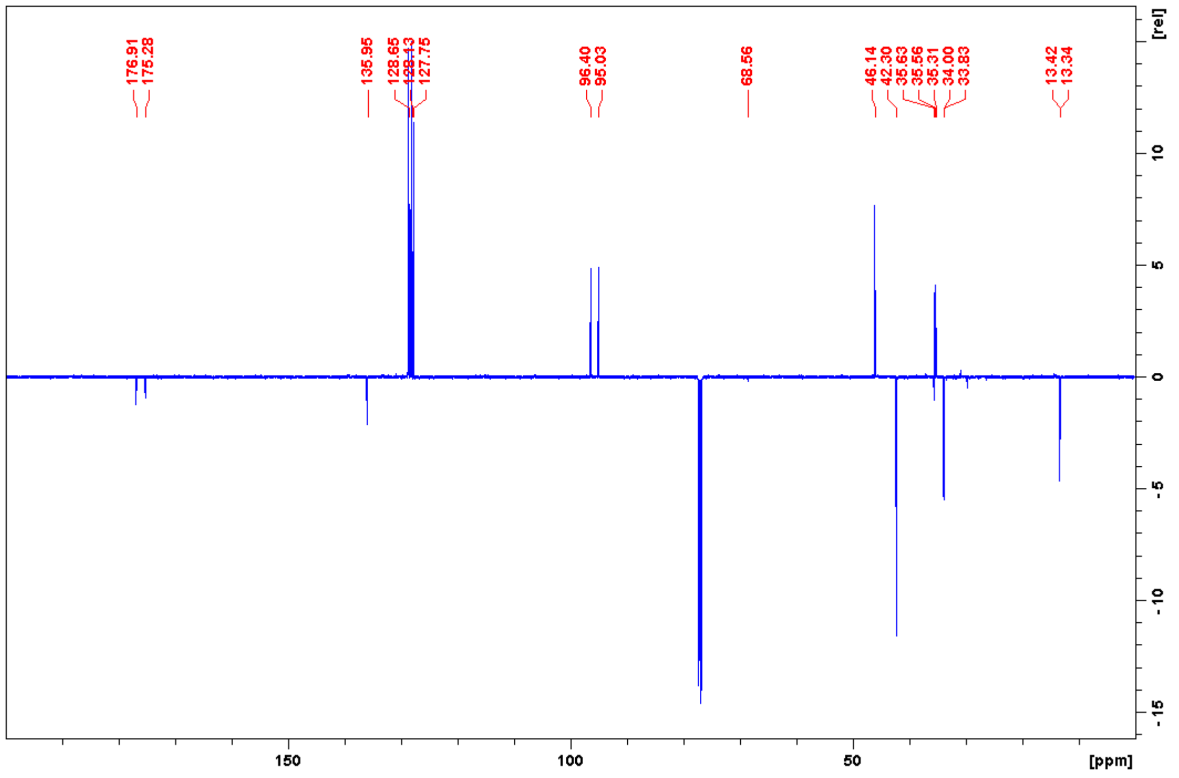

(1*S,2*S**,4*S**,5*S**)-Dimethyl 4-fluoro-5-(phenylselanyl)cyclohexane-1,2-dicarboxylate; (*rac*)-30**

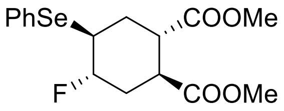

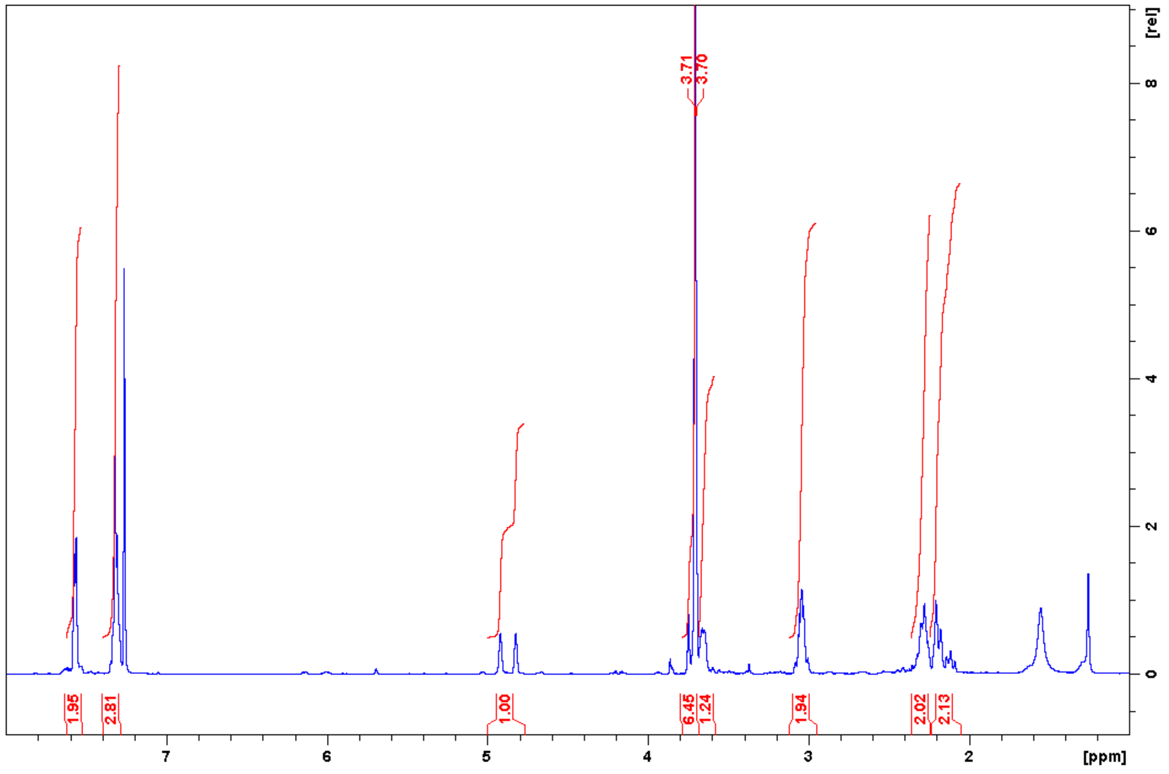

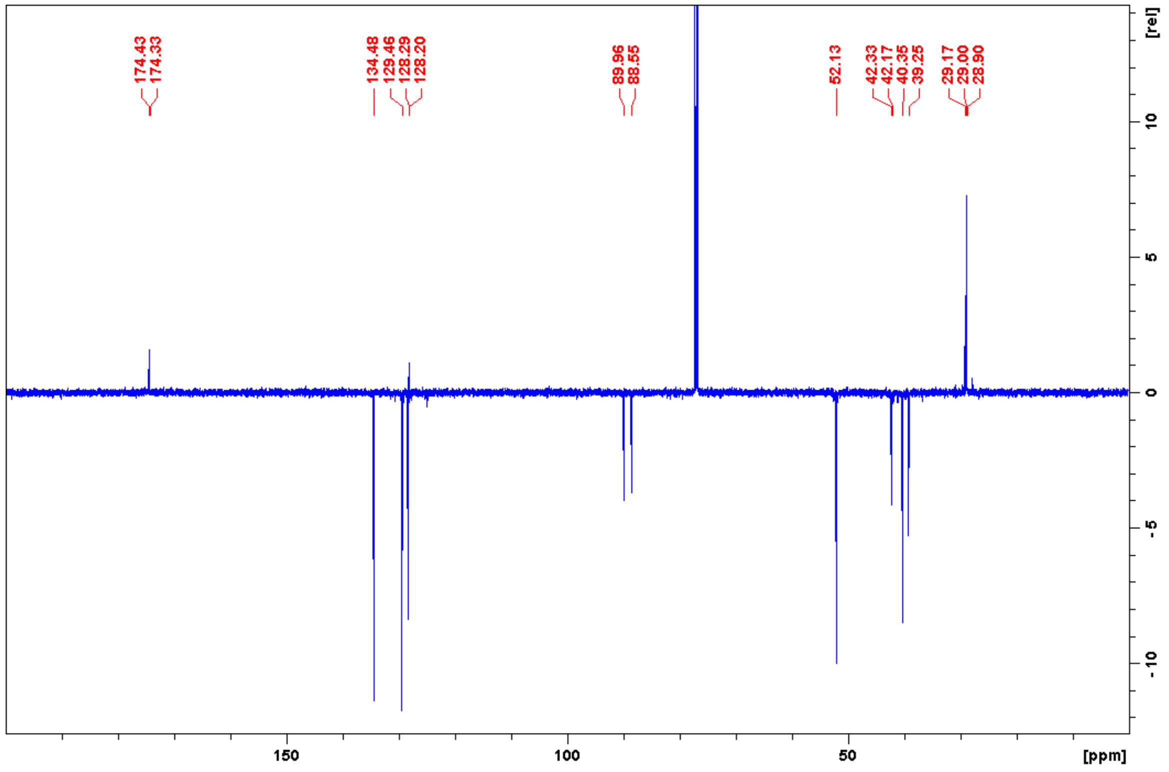

(1*R,2*S**)-Dimethyl 4-fluoro-5-(phenylselanyl)cyclohexane-1,2-dicarboxylate;** (*rac*)-**31**

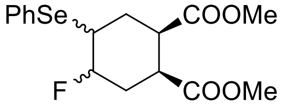

(9:5 mixture of 2 diastereomers)

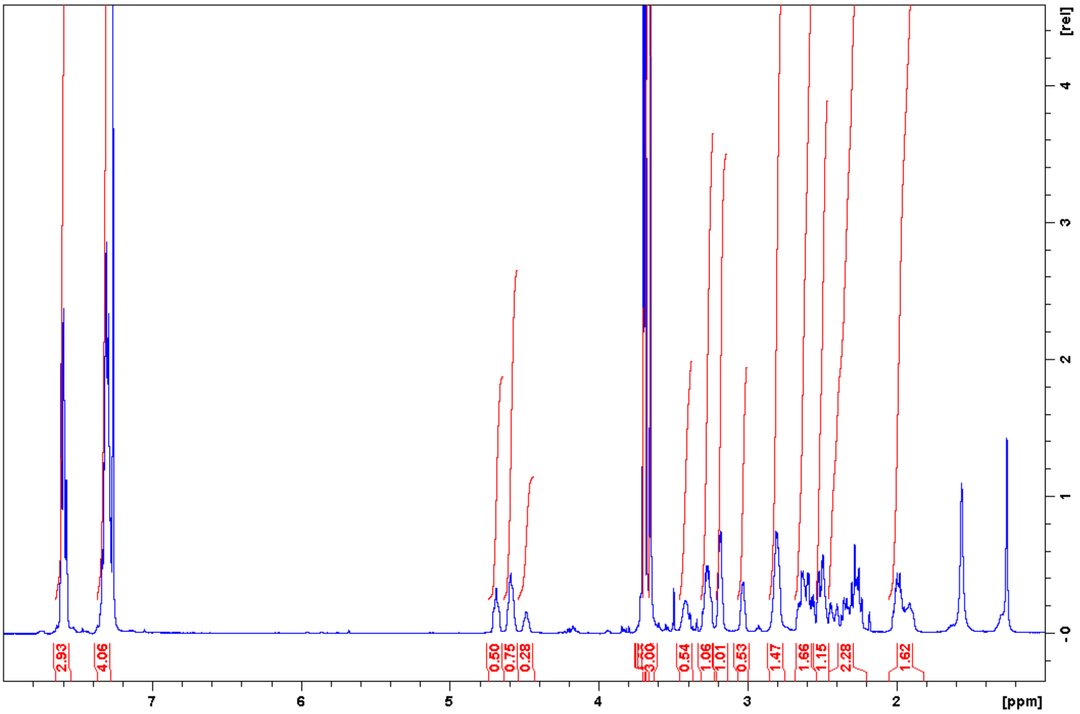

(3*R,3a*R**,5*S**,6*S**,6a*S**,7*R**)-Methyl 2-oxo-6-(phenylselanyl)hexahydro-2*H*-3,5-methanocyclopenta[b]furan-7-carboxylate; (*rac*)-32**

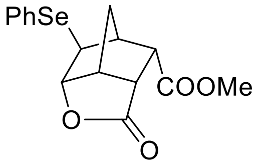

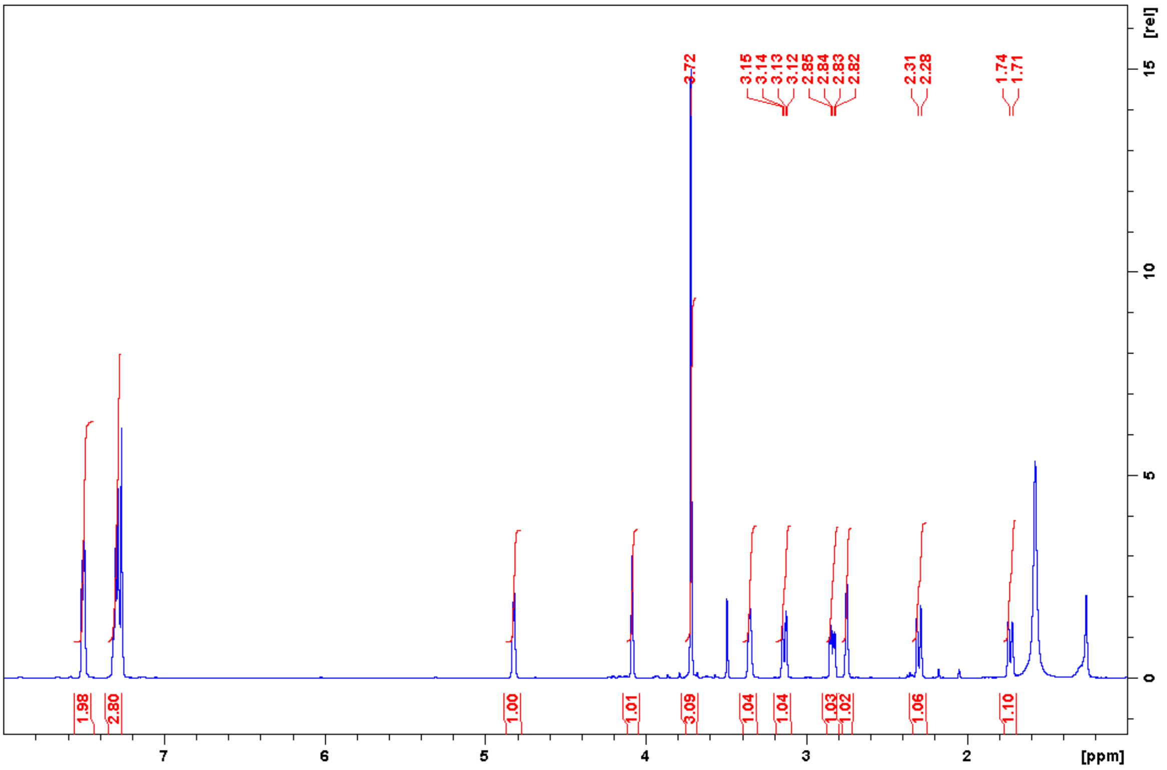

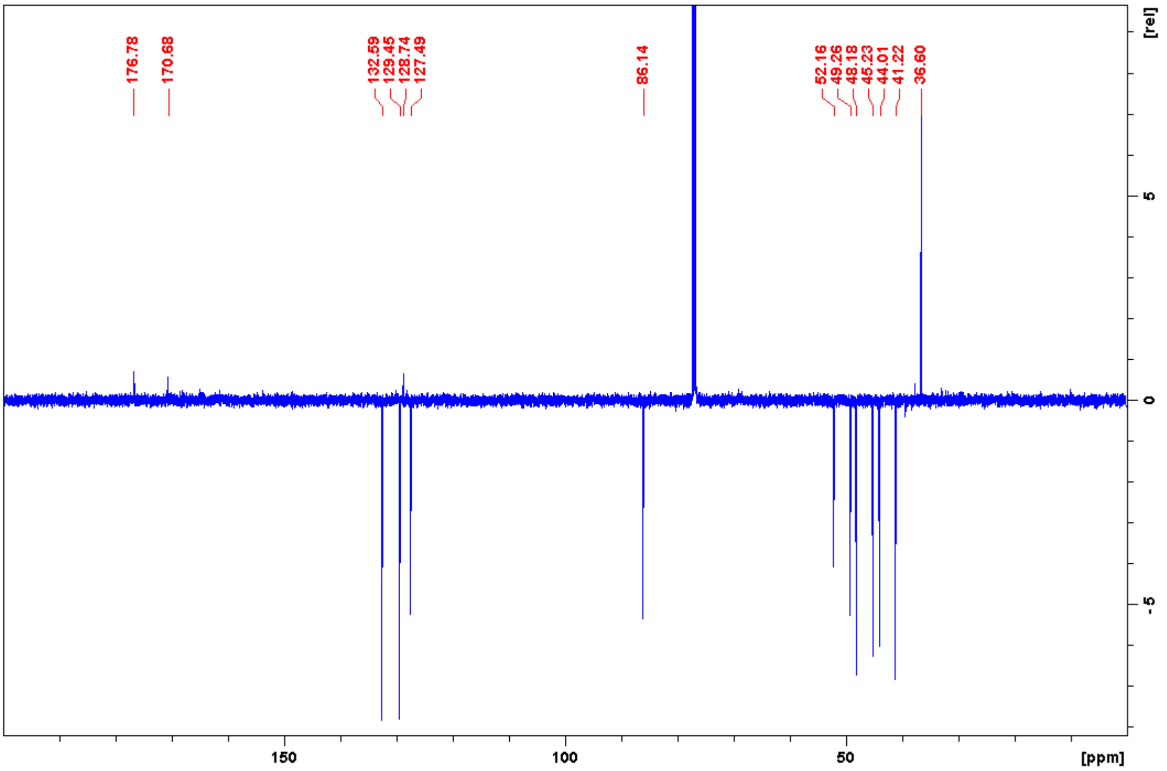

(1*S,2*S**,5*S**)-Dimethyl 5-fluorocyclohex-3-ene-1,2-dicarboxylate; (*rac*)-33**

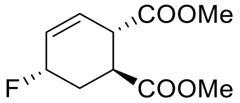

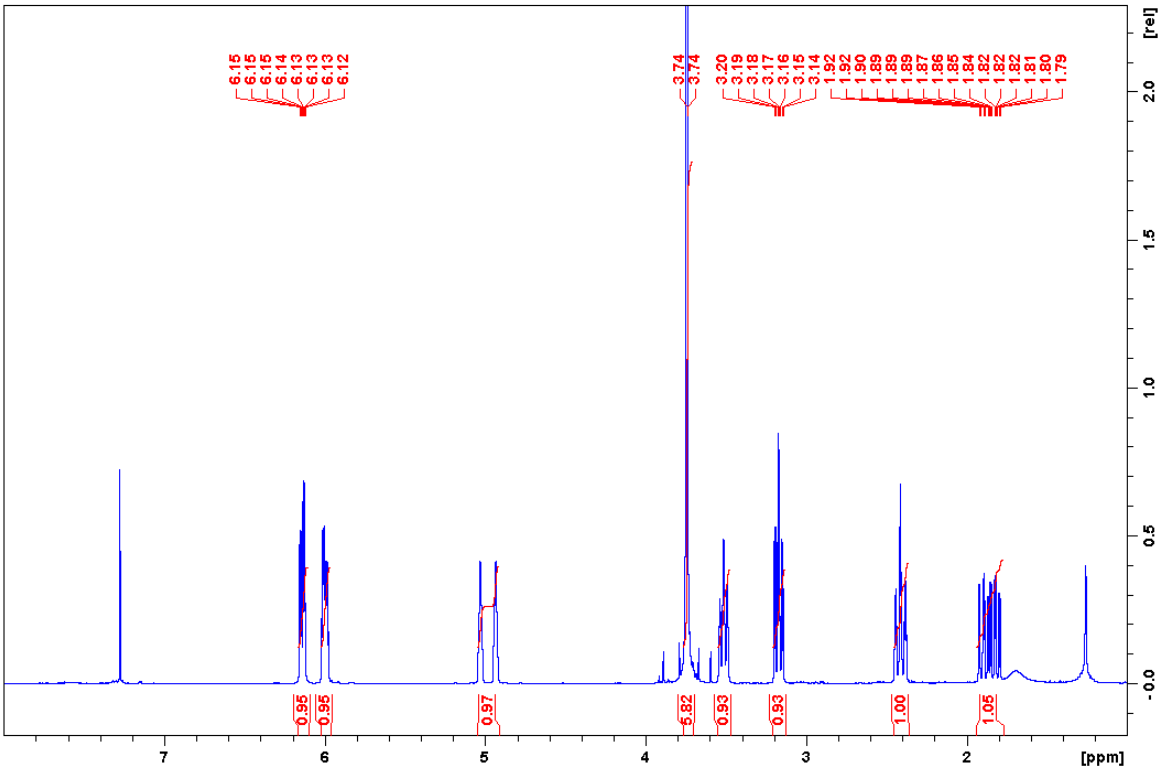

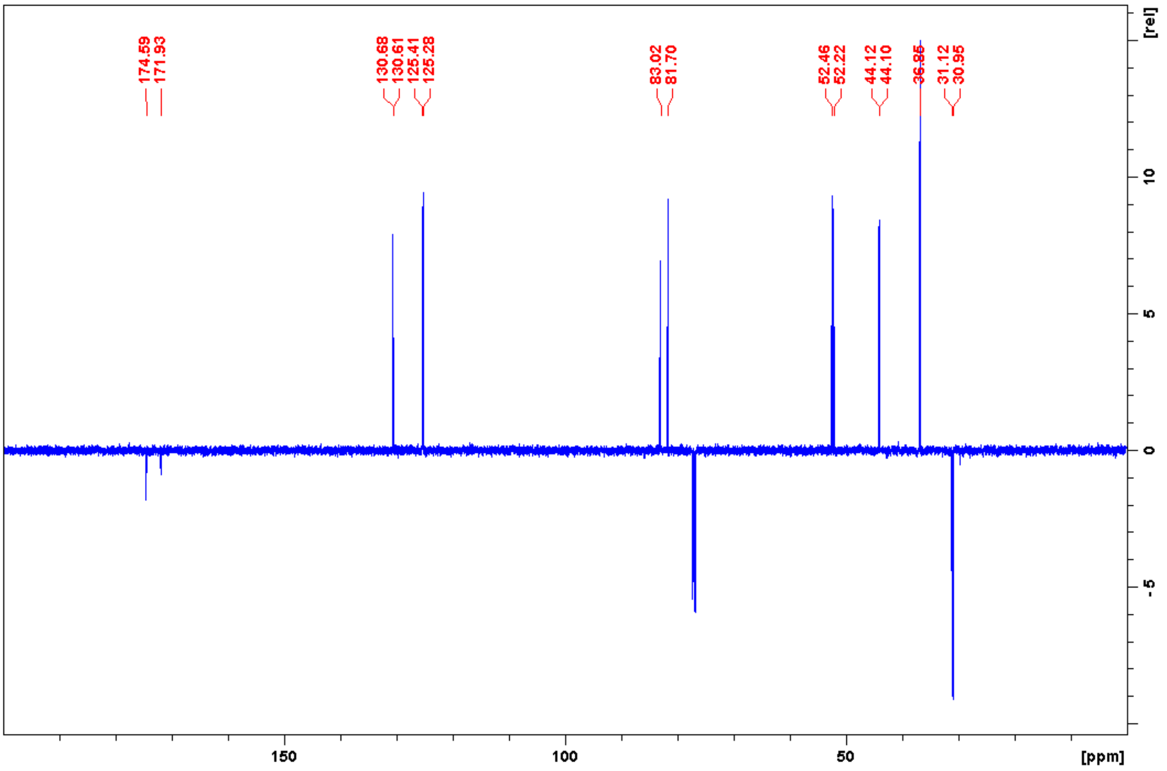

(±)-Dimethyl cyclohexa-2,4-diene-1,2-dicarboxylate; (*rac*)-34

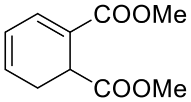

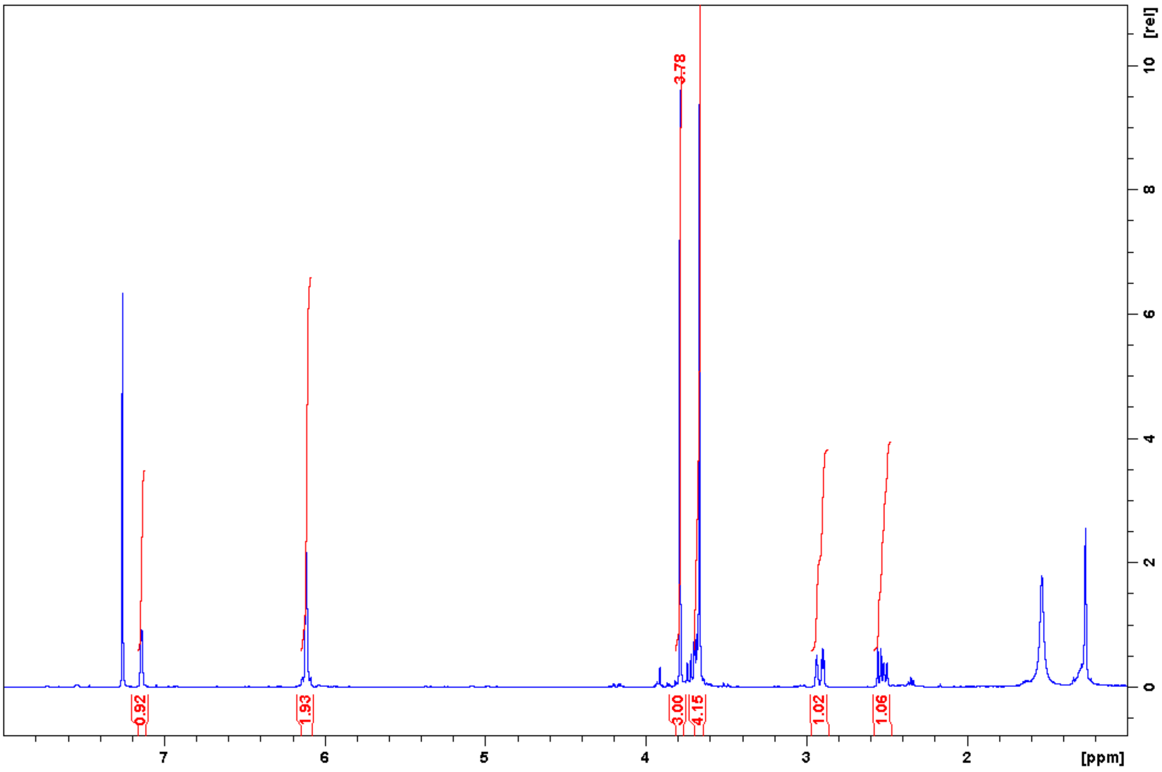

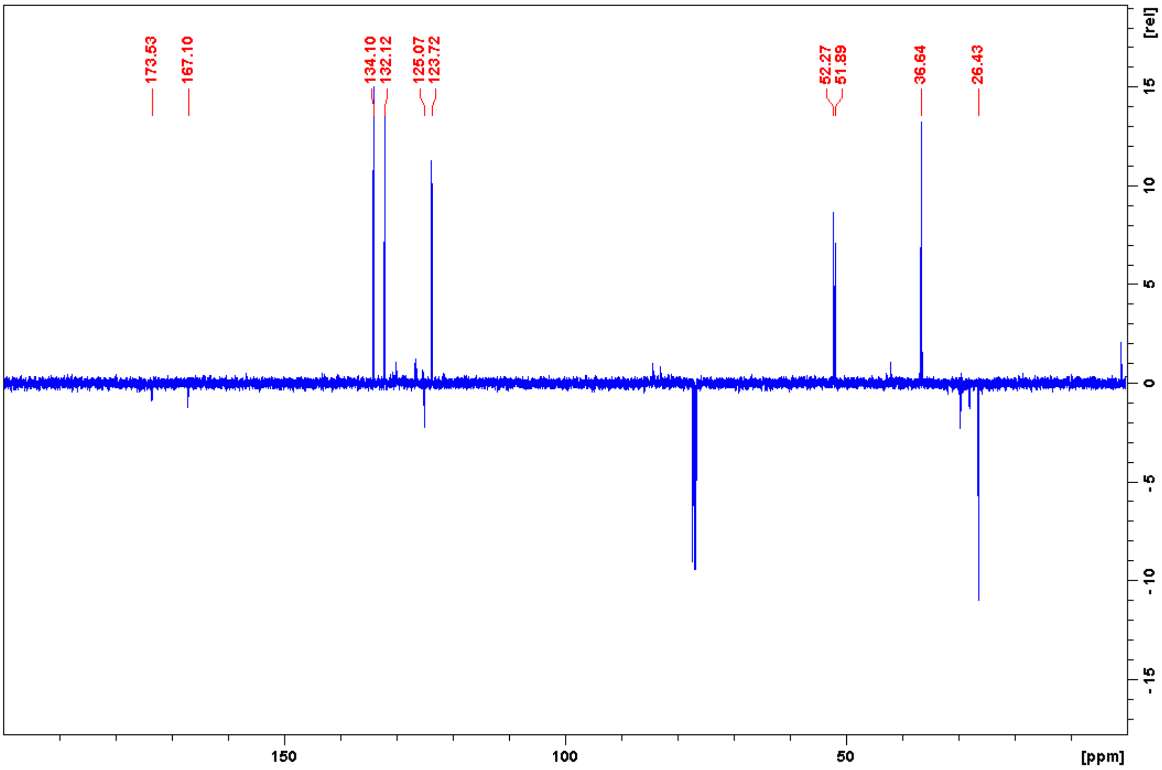

(1S*,2R*,5S*)-Dimethyl 5-fluorocyclohex-3-ene-1,2-dicarboxylate; (rac)-35

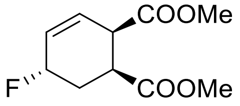

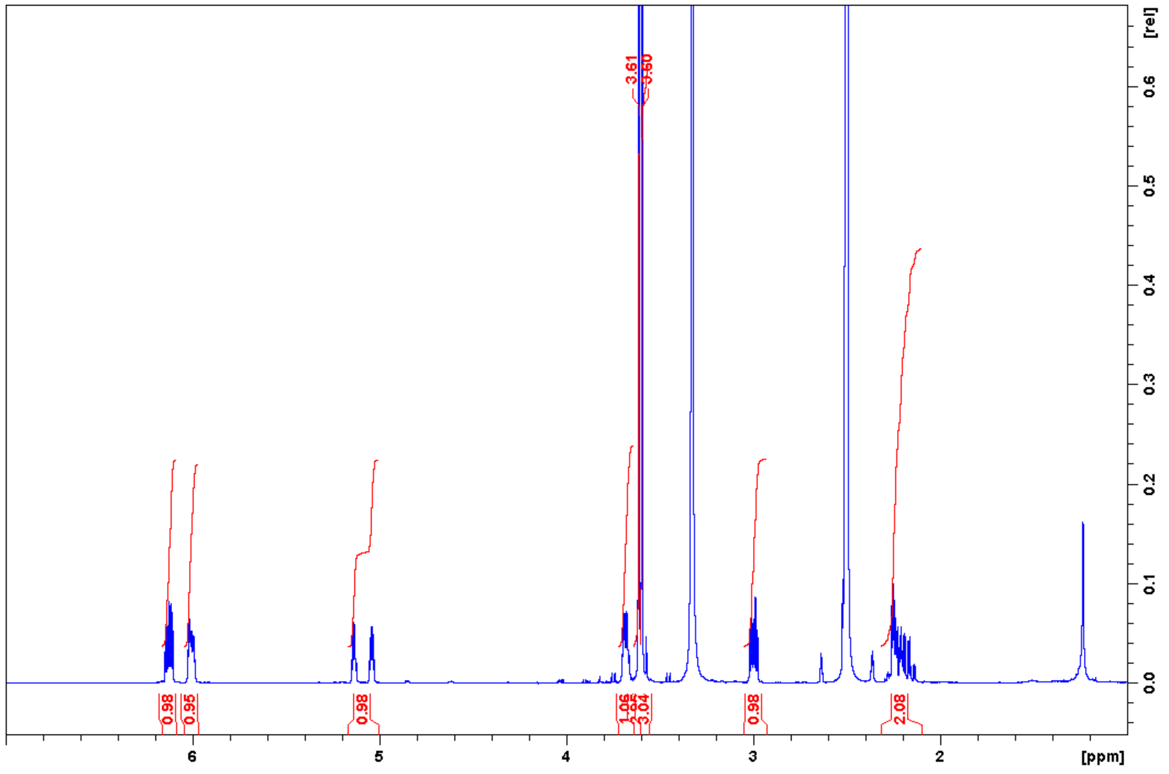

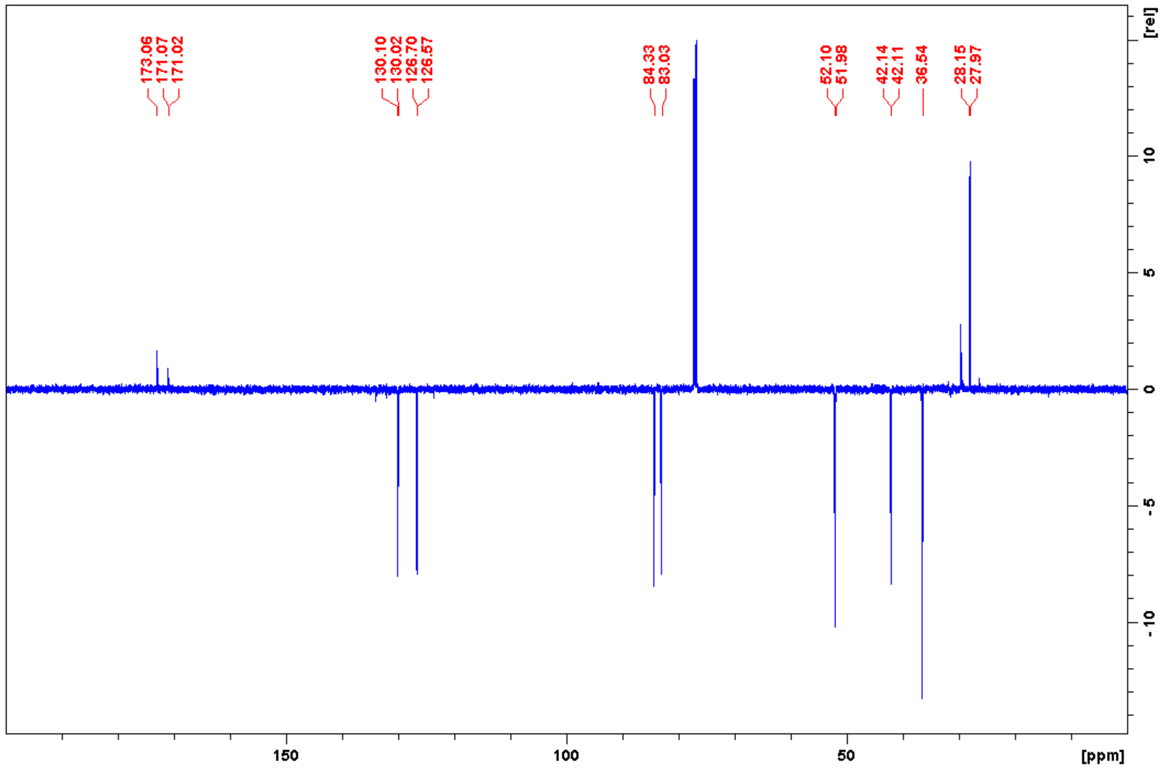

Dimethyl (1*R,2*S**,4*R**)-4-fluorocyclohexane-1,2-dicarboxylate; (*rac*)-36**

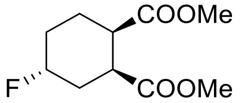

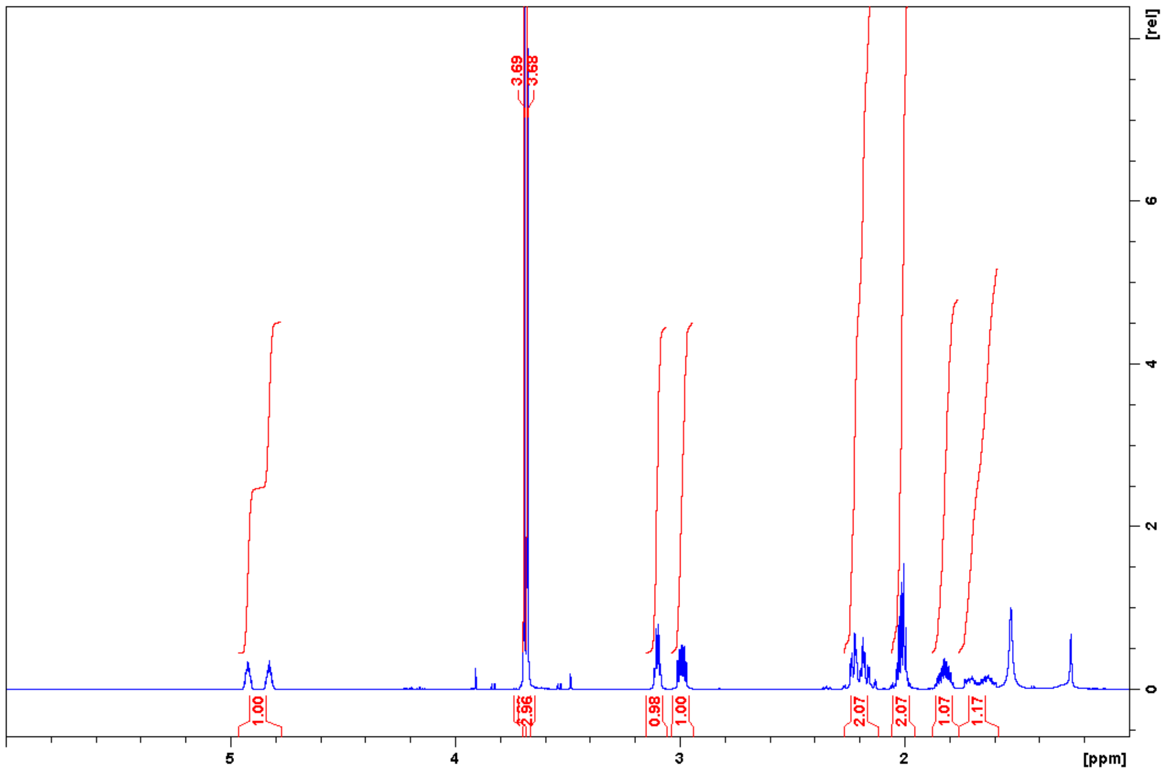

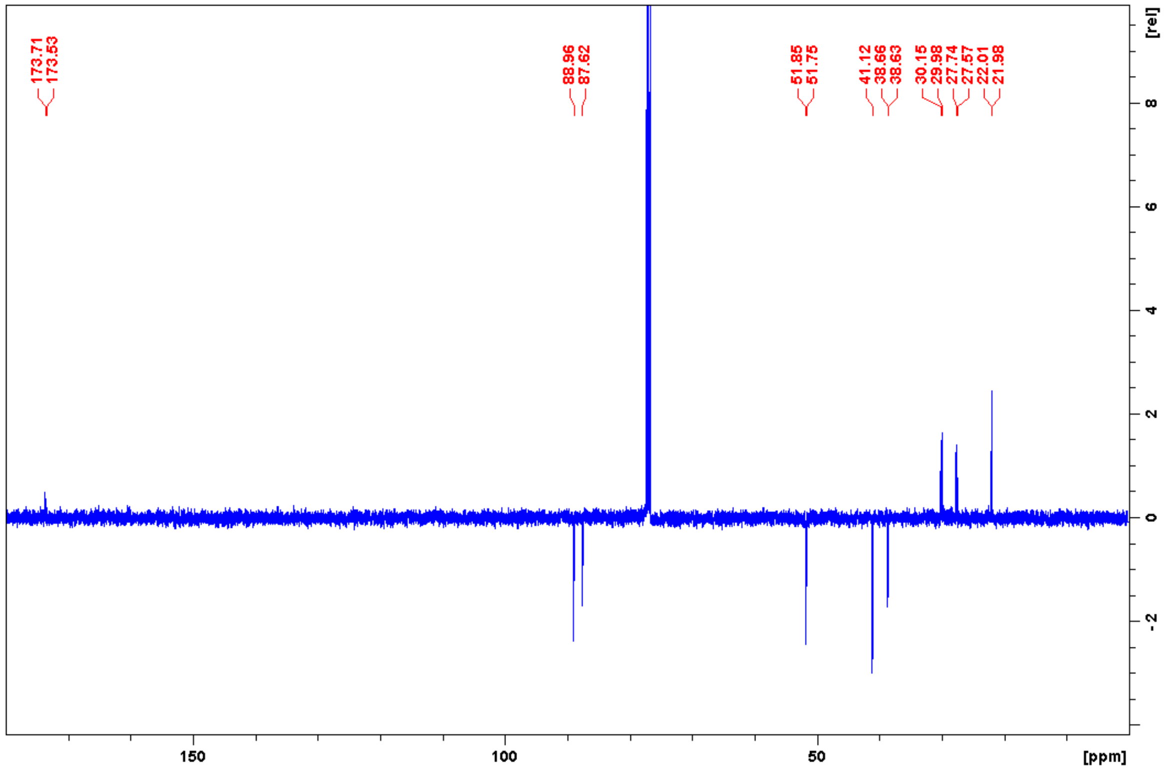

Supplement: File 1 — Characterization and NMR data of the new compounds. [file Beilstein_J_Org_Chem-16-2562-s001.pdf]
